# Sex differences in the regulation and function of cellular immunity in *Drosophila*

**DOI:** 10.1371/journal.pgen.1012151

**Published:** 2026-07-10

**Authors:** Alexandra Dvoskin, Kevin Y. L. Ho, Michael Allara, Nicola Janz, Elizabeth Rideout, Juliet R. Girard, Guy Tanentzapf

**Affiliations:** 1 Department of Cellular and Physiological Sciences, University of British Columbia, Vancouver, British Columbia, Canada; 2 Wyss Institute for Biologically Inspired Engineering, Harvard University, Boston, Massachusetts, United States of America; 3 Department of Biology, University of Massachusetts Boston, Boston, Massachusetts, United States of America; Instituto Leloir, ARGENTINA

## Abstract

Sex differences in development and physiology are prevalent in animals. One physiological system with pronounced differences between the sexes is the immune system: the immune response in humans differs between sexes and results in differential susceptibility of males and females to autoimmune diseases, malignancies, and infectious diseases. However, much remains to be discovered about the mechanisms underlying these sex-based differences in immunity. Here, we use the *Drosophila* hematopoietic organ, the lymph gland, as a model to investigate sex differences in cellular immunity and determine the underlying mechanisms. We find that, in line with their smaller body size, males have smaller lymph glands than females that contain fewer blood progenitors and produce less immune cells. Single cell RNA-seq analysis of the lymph gland showed that they expressed sex determination genes and identified substantial sex-specific differences in gene expression. By manipulating the sexual identity of different cell types in the lymph gland we show that a subset of these sex differences are controlled by organ-intrinsic mechanisms involving the hematopoietic niche. Importantly, we find a differential response between males and females to changes in insulin signaling, an important regulator of the immune response in the niche. Finally, we provide evidence for differences in the cellular immune response following infection between males and females. Overall, our results provide mechanistic insight into how sex differences in immunity are established.

## Author summary

Our paper deals with a fundamental question in biology, how does the sex of an organism influence its anatomy and physiology. In particular, we focus on sex-differences in immunity and stem cell function. We establish the Drosophila larva as a model for analysing sex-based differences in cellular immunity. Cellular immunity in Drosophila is based on the production of multiple types of mature immune cells from blood progenitors and takes place in the fly hematopoietic organ, the larval lymph gland. We find that sex controls the number of various cell types in the lymph gland (progenitors, and specific varieties of mature blood cells). These sex-differences vary by cell type and change depending on whether flies are raised under homeostatic or infection conditions. We provide insight into the mechanisms that mediate these sex differences, identifying a possible role for the hematopoietic niche and insulin signaling. Taken together our work not only serves as an initial characterization of baseline sex-differences in fly hematopoiesis and cellular immunity but also identifies important areas for future exploration.

## Introduction

Sex differences exist across many species, where such differences reflect variations in reproductive strategies, ecological roles, and physiological demands [1,2]. In many animals, males and females differ in terms of overall body size, muscle mass, fat storage, and reproductive tissue development [3–13]. These differences often arise early in development, persist throughout life, and manifest at multiple levels, from the individual cell, to tissues, organs, and entire organisms. Sex differences are genetically encoded and typically involve hormonal signals that control animal development and homeostasis [14]. *Drosophila* has proven to be a powerful model for exploring the genetic underpinning and signaling mechanisms that underlie sex differences [15–18]. In *Drosophila*, sex differences arise early, appearing during embryogenesis, and are pronounced in the adults. *Drosophila* males are typically smaller than females, and exhibit marked differences from females in appearance, behavior, and physiology.

It is well established, both in epidemiological and experimental studies, that there are extensive sex differences in immunity in humans [19,20]. This has important implications in health and disease, as evidenced, for example, by sex differences in the ability to fight certain infections, respond to vaccines, or by the higher prevalence of multiple autoimmune conditions in females. It is speculated that sex differences in immunity are attributed to either genetic or hormonal factors and several mechanistic differences in immunity have been uncovered. On a cellular level, innate detection of pathogens is known to differ between males and females, as sex differences have been found in the induction of toll-like receptors (TLR) and the antiviral type I interferon (IFN) response [21,22]. Moreover, in both humans and rats, females show a higher activity of monocytes, macrophages and dendritic cells [23,24]. Females also tend to mount a more robust adaptive immune response than males by having a higher proportion of active T cells during certain viral infections [25], as well as increased expression, associated with estrogen response elements in their promoter regions, of antiviral and inflammatory genes [26].

Although immunology studies in humans have increasingly included sex as a variable, this has not been the case in *Drosophila*, an important genetic model system for immunity. A recent literature survey found that out of over 1000 papers about the adult immune system in *Drosophila,* almost half did not report sex and only 13% reported results for both males and females separately [27]. In the small number of published immunology studies in *Drosophila* that took sex into account, it has been found that there were differences in immune response and survival following infection, with outcomes being pathogen specific. Specifically, viral infections have been found to result in a higher rate of mortality in males than females, with surviving females exhibiting increased infertility [28]. Bacterial infection studies have shown a complex picture, with sex differences in survival rates following infection in certain strains of bacteria, regardless of whether the bacterial strain in question was gram negative or gram positive [29–33]. For example, females were shown to be more susceptible to infection with *Enterococcus faecalis* but less susceptible to *S. aureus* or to *Lactococcus lactis* infection, all of which are gram positive bacteria [27,31,32]. Some of this variation is accounted for by sex-specific differences in the regulation of both IMD and Toll immune pathways between males and females following infection [30,31]. There is also some indirect evidence of sex differences in the cellular immune response, specifically, hemocytes functioning downstream of the Jun N-terminal kinase (JNK) pathway in repairing tissue damage induced by UV irradiation were only seen in males [34,35].

Work in *Drosophila* on hematopoiesis and immunity has focused extensively on the lymph gland, an organ that is responsible for the cellular immune response during larval stages [36]. The primary lobe of the lymph gland is the main site of hematopoiesis in *Drosophila* larval stages and is typically described as being made up of 3 distinct zones: the Posterior Signaling Center (PSC), a group of a few dozen cells that are thought to have a stem cell niche-like function, the medullary zone (MZ), which houses blood progenitors, and the cortical zone (CZ) which holds differentiated blood cells [37]. Multiple signaling pathways act in the lymph gland to regulate hematopoiesis including the Wingless, Hedgehog, JAK/STAT, BMP and Notch pathways [37–42]. Mutations that perturb these signaling pathways result in a variety of defects in lymph gland homeostasis, ranging from depletion of the progenitor population, overproduction of various mature blood cell lineages, and expansion of the PSC [38–41,43]. Another major signaling pathway known to act in the lymph gland is the insulin/insulin-like growth factor signaling pathway (IIS) [44–46]. In the lymph gland, the IIS pathway has been shown to regulate both the maintenance and differentiation of blood progenitors as well as maintenance of the PSC [47,48]. Moreover, at least two *Drosophila* insulin-like peptides (ILPs), secreted by insulin-producing cells in the brain (*Ilp2*) and the fat body (*Ilp6*), are known to act in the lymph gland to control hematopoiesis [49–52].

Here we analyzed sex differences in the *Drosophila* lymph gland under homeostatic conditions as well as following bacterial infection. We identified sex differences in the overall cell number and within specific cell types of the lymph gland. Analysis of single-cell RNA-Seq data from the lymph gland uncovered extensive sex-differences in gene expression in all cell types of the gland. Further investigation into the mechanisms that mediate sex-differences identified a source of some of the relevant regulatory signals, revealing a role for insulin signaling in this process. Finally, we explored whether and how sex differences extend to the cellular immune response following infection.

## Results

### Lymph glands exhibit sex differences

**Fig 1:**
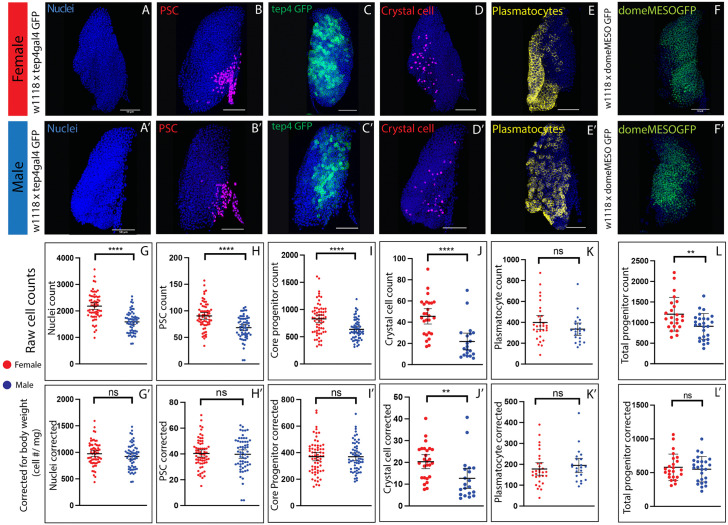
Characterizing sex differences in the lymph gland. (A-E) Representative images of the female *w1118 x tep4 gal4GFP* lymph gland stained with ToPro, as well as Antp (B) to mark for PSC cells, endogenously expressed GFP in tep4 + cells (C) to mark progenitors, Hnt (D) to mark for crystal cells, and P1 (E) to mark for plasmatocytes. **(F)** Representative image of the female *w1118 x domeMESO GFP* lymph gland stained with ToPro. (A’-E’) Representative images of the male *w1118 x tep4 gal4GFP* lymph gland stained with ToPro, as well as Antp (B’) to mark for PSC cells, endogenously expressed GFP in tep4 + cells (C’) to mark progenitors, Hnt (D’) to mark for crystal cells, and P1 (E’) to mark for plasmatocytes. (F’) Representative image of the male *w1118 x domeMESO GFP* lymph gland stained with ToPro. **(G-L)** Raw cell counts plotted for both males and females. **(G)** Raw number of nuclei, used to measure organ size, plotted for males and females (female n = 64, male n = 59, p < 0.0001). **(H)** Raw number of PSC cells (Antp+), plotted for males and females (female n = 64, male n = 59, p < 0.0001). **(I)** Raw number of core progenitors (tep4+), plotted for males and females (female n = 64, male n = 59, p < 0.0001). **(J)** Raw number of crystal cells (Hnt+), plotted for males and females (female n = 25, male n = 19, p < 0.0001). **(K)** Raw number of plasmatocytes (P1+), plotted for males and females (female n = 30, male n = 22, p = 0.1661). **(L)** Raw number of total progenitors (domeMESO+), plotted for males and females (female n = 24, male n = 26, p = 0.0058). (G’-K’) Corrected by dividing each female data point by average phenotype weight (*w1118 x tep4 gal4GFP*=2.24mg, *w1118 x domeMESO* GFP = 2.19mg)and dividing each male data point by average phenotype weight (*w1118 x tep4 gal4GFP*=1.72mg, *w1118 x domeMESO* GFP = 1.65mg). (G’) Corrected nuclei count, used to measure organ size (female n = 64, male n = 59, p = 0.2107). (H’) Corrected number of PSC cells (Antp+) (female n = 64, male n = 59, p = 0.7819). (I’) Corrected number of core progenitors (tep4+) (female n = 64, male n = 59, p = 0.9705). (J’) Corrected number of crystal cells (Hnt+) (female n = 25, male n = 19, p = 0.0056). (K’) Corrected number of plasmatocytes (P1+) (female n = 30, male n = 22, p = 0.4531). (L’) Corrected number total progenitors (domeMESO+) (female = 24, male = 26, p = 0.6048). **** indicates P < 0.0001, *** indicates P < 0.001, ** indicates P < 0.01, * indicates P < 0.05, ns (non-significant) indicates P > 0.05. Error bars are 95% CI.

To investigate whether there were sex differences between lymph glands in male and female larvae, we measured organ size in late 3rd instar larva using automated cell counts with custom image analysis software (see Methods). We found that lymph glands from male larvae contained an average of 1587 ± 404 cells (n = 59), while lymph glands from females contained on average 2183 ± 501 (n = 63), a difference of ~37% in cell number (Fig 1G). Next, we determined the number of cells in the Posterior Signaling Centre (PSC), the stem cell niche of the lymph gland by staining with an antibody that labels the transcription factor Antennapedia (Antp) and performing cell counts (see methods). These counts showed that lymph glands from male larvae contained an average of 68 ± 22 PSC cells (n = 59), while lymph glands from female larvae contained on average 91 ± 24 PSC cells (n = 63), a difference of ~33% (Fig 1H). We noted that these numbers of PSC cells we recorded were higher than the numbers we and other groups previously reported (for example we reported ~45 PSC cells in Ho et al, 2021). This could partly be explained due to differences in methodology, as we used an antibody staining against Antp to do our PSC counts (S1 Fig), while others used different methods. However, methodology alone did not account of for all of these differences and may represent genotype specific variation. Next, we labelled progenitors in the lymph gland in two ways, using either *Tep4gal4; UAS-GFP* to mark core progenitors or *domeMESO GFP* to mark the total population of progenitors [51]. Cell counts of either the total or core progenitor populations showed that lymph glands from male larvae contained an average of 636 ± 190 core progenitors (n = 59) and 909 ± 297 (n = 26) total progenitors, while lymph glands from female larvae contained on average 830 ± 282 core progenitors (n = 63) 1205 ± 421 (n = 24) total progenitors, a difference of ~33% in both populations (Fig 1I and 1L).

A key required control for interpreting these data is that the drivers themselves are expressed at similar levels between males and females. To study this question, we analysed the efficiency of two lymph gland drivers, *Tep4gal4* and collier-gal4, in males and females. Both drivers were used to express GFP and, using imageJ, we measured intensity across a variety of images with the same imaging settings (laser power, gain and offset). We did this by going through slices of images, finding full cells that were not overlapped with other cells, and measuring expression intensity (see materials and methods). Our results (S2 Fig) show no significant difference between male and female mean intensity for collier-gal4 (p = 0.0740), or for *Tep4gal4* (p = 0.5192).

To determine the number of mature differentiated plasmatocytes and crystal cells we stained the lymph gland with antibodies against the markers P1 and Hindsight (Hnt), respectively, followed by whole lymph gland cell counts (see methods). These cell counts showed that in lymph glands from male larvae there were on average 22 ± 17 crystal cells (n = 20) and 335 ± 134 plasmatocytes (n = 22), respectively. In comparison, in lymph glands from female larvae there were on average of 46 ± 18 crystal cells (n = 28) and 409 ± 172 plasmatocytes (n = 29), a difference of ~210% in crystal cell numbers and ~22% in plasmatocyte numbers between the sexes (Fig 1J-1K).

Because *Drosophila* male larvae are smaller in size than females, we asked if the sex differences we observed between lymph glands from male and female larvae were consistent with the overall size differences between males and females. To account for known sex differences in body size we normalized lymph gland cell counts to body size (see methods, Fig 1G’-1L’). This analysis showed that sex differences in most parameters were eliminated when normalized for body size. Furthermore, normalizing the number of PSC cells, core and total progenitors or plasmatocytes to lymph gland size, measured as the total number of nuclei, which is common practice done in the field, also eliminated sex-based differences (S3 Fig). Importantly, the female bias in crystal cell number was maintained after normalizing for body size. Crystal cell numbers are known to be affected by the lymph gland size, and for this reason, it is common to report the overall percentage crystal cell in the lymph gland rather than raw counts*.* We therefore asked if normalizing for the total number of cells within the lymph gland differed from normalizing to body size. Our data, shown in S4 Fig, showed that similar conclusions could be drawn by normalizing crystal cell numbers to either body size or the total numbers of cells in the lymph gland. Taken together, our data shows striking sex differences in both overall organ size as well as the size of specific cell populations in the larval lymph gland. While these differences generally align with expected organ size based on body size, crystal cells show a uniquely large population size increase in females, that exceeds what would be expected based on overall trends in body size.

### RNA sequencing shows differential gene expression between sexes in the lymph gland

In our previous single cell RNA sequencing analysis of lymph glands, we excluded several sex-specific genes prior to graph-based clustering or visualization. Reanalysis of this data including sex-specific genes shows that sex has a significant effect on the UMAP visualization of lymph gland cells. Specifically, we see two distinct hemispheres in the UMAP, one of which is distinguished by high expression of the male-specific long non-coding RNAs (lncRNAs) *lncRNA:roX1* and *lncRNA:roX2* (S1 Movie). Since we added the same number of male and female larvae to each sample, and roughly half of the cells express *roX1* and *roX2*, this suggests that sex is what distinguishes the two hemispheres of the UMAP. Some of the graph-based cell clusters are physically split across the distinct hemispheres in the three-dimensional UMAP (S1 Movie). The affected clusters correspond to lymph gland progenitors (MZ), intermediate cells (IZ and proPL), and plasmatocytes (PL; S1 Movie).

**Fig 2:**
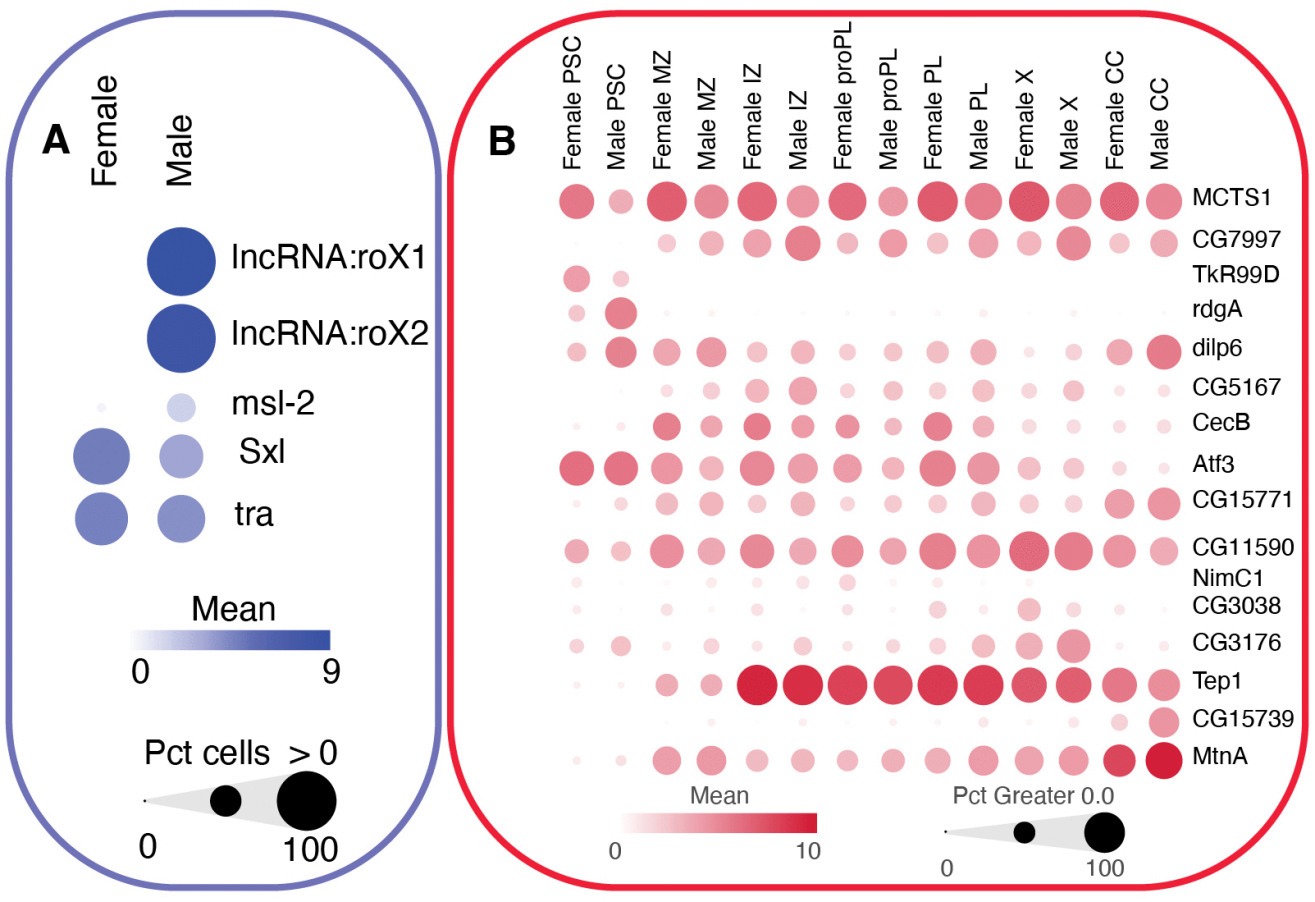
Single-cell RNA sequencing of lymph gland cells. **(A-B)** Bubble plots showing the mean expression (Mean) of each gene (color intensity) and percentage (Pct) of those cells which express that gene (size). **(A)** Expression of known sex-specific genes in the scRNA-data that have been disaggregated by sex. *lncRNA:roX1*, *lncRNA:roX2*, and *msl-2* are enriched in male cells while *Sxl* and *tra* are enriched in female cells. **(B)** Selected genes that were found to be enriched in male or female cells in specific lymph gland zones. PSC, posterior signaling center; MZ, medullary zone; IZ, intermediate zone; proPL, proplasmatocyte; PL, plasmatocyte; X, mitotic cluster; CC, crystal cell.

We used the expression levels of both *roX1* and *roX2* to disaggregated cells by sex*.* Cells with high *roX1* and *roX2* expression (8865 in total, ~ 42% of cells) were categorized as male, while cells with low *roX1* and *roX2* expression (10242 in total, ~ 48% of cells) were categorized as female. Cells which showed high expression of one *roX* RNA but not both (2050 in total, ~ 10% of cells) were excluded from further analysis as their sex was ambiguous. When we compared gene expression across the sexes, we observed differential expression of several genes involved in sex determination. For example, we saw that *msl-2* was enriched in male cells, but *Sxl* and *tra* were enriched in female cells (Fig 2A and S1 Table). These data suggest that the sex determination genes are expressed in lymph gland cells.

We identified differentially expressed genes (DEGs) which were significantly enriched in either males or females using ANOVA analysis (genes that were greater than or equal to the fold change threshold of 1.5 and less than or equal to the false discovery rate threshold of 0.0001 were defined as significantly enriched; S1 File). We then compared gene expression of male and female cells in each graph-based cluster including the PSC, MZ, IZ, proPL, X, PL, and CC. This allowed us to determine sex-specific differences within each cell type. While some sex-specific DEGs are differentially expressed across all populations of male or female lymph gland cells, many are only differentially expressed in male or female cells in one or more specific zones (S1 File). For example, female PSC cells are uniquely enriched in *TkR99D*, which encodes a receptor for tachykinin-like neuropeptides, while male PSC cells are specifically enriched for *rdgA*, a gene encoding a diacylglycerol kinase involved in phospholipase C signaling (Fig 2B). To identify potential pathways or processes involved in each cluster by sex, we performed gene set enrichment analysis on the sex-specific DEGs we identified in each cluster (S1 File). For example, we found that female MZ, IZ, proPL, and PL cells were enriched in genes involved in the humoral immune response downstream of the Toll and Imd signaling pathways (*CecB*; Fig 2B and S1 File). Overall, many of the sex-specific DEGs we identified have human orthologs and some have been previously implicated in blood development, disease, or immune function (S1 File). These sex-specific genes are of interest for future study and may provide insight into the mechanisms that underlie sex differences in hematopoiesis or immunity.

### We find no evidence that Sex differences in the lymph gland are mediated by the CNS

Sex differences between tissues can arise due to cell-autonomous or non-cell-autonomous mechanisms. For example, it has been shown that systemic signals from tissues such as the fat body, the muscles, or the central nervous system (CNS) can influence cell and body growth in the fly [49–51]. To test whether sex differences in the lymph gland were due to a systemic signal originating from the CNS or fat body, we employed a strategy where we feminized these tissues in an otherwise genetically male fly and asked how this impacted overall organ size as well the size of individual cell populations in the lymph gland. To change the sexual identity of different tissues we expressed sex determination gene *transformer* (*tra*) in males [15,16,53–60]. Normally, a functional Tra protein is only produced in females (Tra^F^), where it specifies most aspects of sexual differentiation and development [61–63]. When Tra^F^ is expressed in males, it is sufficient to induce the development of female-specific traits [64]. It has been previously shown that expression of the Tra^F^ transgene alters sex-specific gene expression. For example, Hudry et al [16] demonstrated that expression of the Tra^F^ transgene in intestinal progenitors in an otherwise *tra* null female was sufficient to restore female-specific isoform expression of the *tra* gene. Moreover, there is an extensive body of literature documenting how targeted expression of the Tra^F^ transgene alters sex-specific traits on the single cell level in terms of metabolism, gene expression, physiology, and appearance [16,65–67].

To control for differences in the lymph gland due to variation in the genetic background, we analysed flies heterozygous for the Tra^F^ construct (Tra^F^/+) and flies heterozygous for the Gal4 driver (Gal4/+) in addition to the experimental group of flies having both the Gal4 and Tra^F^ transgene (Gal4/Tra^F^). We then compared males and females from each of the two control groups as well as the experimental group. This meant we were comparing 6 different genotypes to each other, which required us to use a two-way ANOVA, post-hoc Tukey’s test (see methods) to ask if any differences we observed were statistically significant. This test analyzes the significance of changes between groups and provides a “sex:genotype interaction constant” to determine whether the data supported the existence of sex differences between males and females under the experimental treatment.

**Fig 3:**
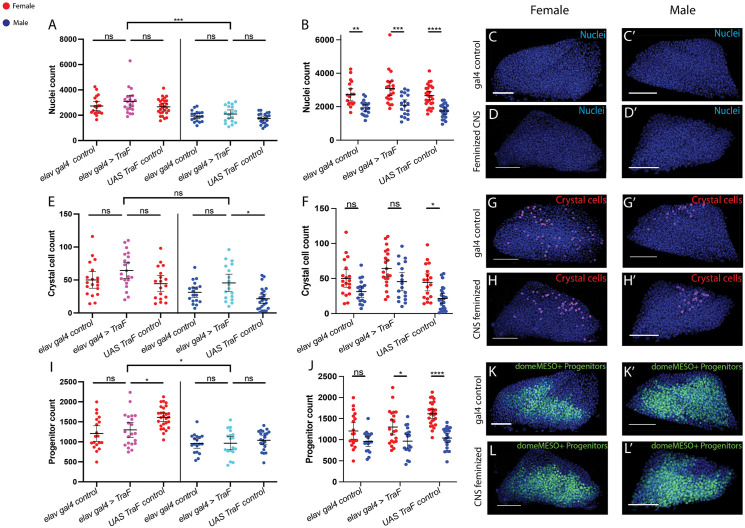
Feminizing the CNS. **(A-B)** Raw cell counts for total number of nuclei, stained with ToPro. Quantification using a two-way ANOVA, post-hoc Tukey’s test for: *elav-Gal4* control females (n = 19), *elav-Gal4 > Tra*^*F*^ females (n = 21), *UAS Tra*^*F*^ control females (n = 27), *elav-Gal4* control males (n = 18), *elav-Gal4 > Tra*^*F*^ males (n = 18), *UAS Tra*^*F*^ control males (n = 21). The genotype:sex interaction constant was not significant (p = 0.8059). **(C)** Representative image of *elav-Gal4* control female stained with ToPro. (C’) Representative image of *elav-Gal4* control male stained with ToPro. (D,D’) Representative image of *elav-Gal4;domeMESO GFP > UAS Tra*^*F*^ female (D) and male (D’) stained with ToPro. **(E-F)** Raw cell counts for total number of crystal cells, stained with Hnt. Quantification using a two-way ANOVA, post-hoc Tukey’s test for: *elav-Gal4* control females(n = 19), *elav-Gal4 > Tra*^*F*^ females(n = 21), *UAS Tra*^*F*^ control females(n = 18), *elav-Gal4* control males(n = 18), *elav-Gal4 > Tra*^*F*^ males(n = 18), *UAS Tra*^*F*^ control males(n = 24) The genotype:sex interaction constant was not significant (p = 0.8966). (G, G’) Representative image of *elav-Gal4* control female (G) and male (G’) stained with Hnt. (H, H’) Representative image of *elav-Gal4;domeMESO GFP > UAS Tra*^*F*^ female (H) and male (H’) stained with Hnt. **(I-J)** Raw cell counts for total number of progenitors, expressing GFP in domeMESO+ cells. Quantification using a two-way ANOVA, post-hoc Tukey’s test, for: *elav-Gal4* control females (n = 19), *elav-Gal4 > Tra*^*F*^ females(n = 21), *UAS Tra*^*F*^ control females(n = 27), *elav-Gal4* control males (n = 18), *elav-Gal4 > Tra*^*F*^ males (n = 18), *UAS Tra*^*F*^ control males (n = 21) The genotype:sex interaction constant was not significant (p = 0.0542). (K, K’) Representative image of *elav-Gal4* control female (K) and male (K’), expressing GFP in domeMESO+ cells. (L, L’) Representative image of *elav-Gal4;domeMESO GFP > UAS Tra*^*F*^ female (L) and male (L’), expressing GFP in domeMESO+ cells. **** indicates P < 0.0001, *** indicates P < 0.001, ** indicates P < 0.01, * indicates P < 0.05, ns (non-significant) indicates P > 0.05. Error bars indicate 95% CI.

When we feminized post-mitotic neurons using *elav*-Gal4, there was no significant effect of genetic background on overall lymph gland size, as males or females of both Tra^F^ and *elav*-Gal4 control flies had a similar number of cells in their lymph gland compared to each other or the experimental group (Fig 3A-3B). Moreover, using these controls suggested that CNS feminization had no significant impact on lymph gland size. Indeed, analysing the sex:genotype interaction constant (Table 1) showed that sex differences in the total number of cells in male and female lymph glands were not altered by feminization of the CNS. Similarly, sex differences in the crystal cells or progenitor numbers did not appear to change upon feminization of the CNS, a conclusion that was supported by analysis of the sex:genotype interaction constant (Fig 3E-3H and 3I-3L, respectively; Table 1). To confirm the findings observed when elav-Gal4 we repeated this analysis with another neuronal driver with a more restricted pattern of distribution, C2-Gal4 [68] and found similar results to those obtained with *elav-gal4* (S5 Fig)*.* Taken as a whole, since the effects we saw upon feminization of the CNS were not statistically significant we failed to find evidence that sex differences in the lymph gland were mediated by the CNS.

**Table 1:** Interaction constants for feminization experiments. Sex:Genotype interaction constants derived from two-way ANOVA, post-hoc Tukey’s test performed on data from Figs 3-6 (see methods).

	Nuclei	Crystal cells	Progenitors
Feminized CNS (Fig 3)	0.8059	0.8966	0.0542
Feminized FB (Fig 4)	0.7379	0.7910	0.0045*
Feminized Progenitors (Fig 5)	0.0379*	0.8740	0.0069*
Feminized Niche (Fig 6)	0.0139*	0.0022*	0.0529

* denotes a significant interaction constant (p < 0.05).

### We find no evidence that sex differences in the lymph gland are mediated by the fat body

Given that systemic signals from the fat body can also influence cell and body growth [60,69], we asked whether the sexual identity of this key organ played a role in mediating sex differences in the lymph gland. To this end, we feminized the fat body in genetically male flies using the Tra^F^ transgene and studied how this impacted individual cell population or overall organ size in the lymph gland. As we did for the CNS feminization experiments, we compared experimental groups of flies having both the *R4*-Gal4 and Tra^F^ transgene (*R4*-Gal4/Tra^F^) to control flies heterozygous for either the Tra^F^ construct (Tra^F^/+) or the Gal4 driver (*R4*-Gal4/+). We then performed statistical analysis by calculating the sex:genotype interaction constant (Table 1) to determine if any sex differences in the total number of cells, crystal cells, or progenitors in male and female lymph glands were altered by feminization of the fat body in males.

**Fig 4:**
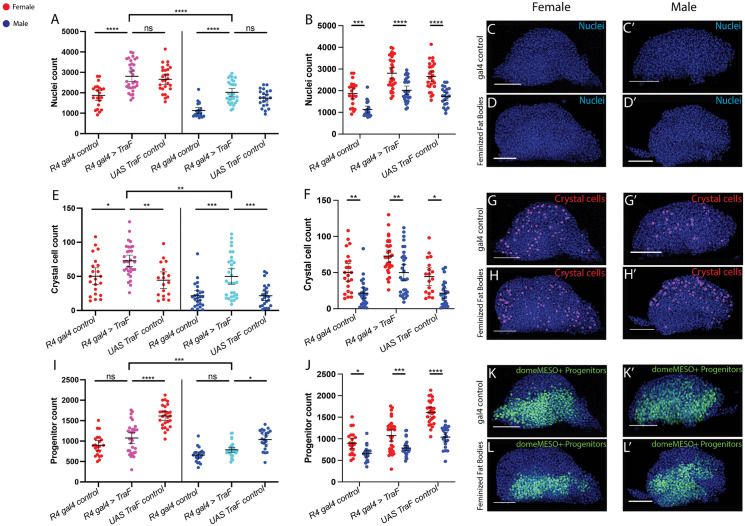
Feminizing the fat bodies. **(A-B)** Raw cell counts for total number of nuclei, stained with ToPro. Quantification using a two-way ANOVA, post-hoc Tukey’s test, for: *R4-Gal4* female control (n = 22), *R4-Gal4* feminized females (n = 32), *UAS Tra*^*F*^ control females (n = 27), *R4-Gal4* control males (n = 21),*R4-Gal4* feminized males (n = 31), *UAS Tra*^*F*^ control males (n = 21). The genotype:sex interaction constant was not significant (p = 0.7379). (C, C’) Representative image of *R4-Gal4* control female (C) and male (C’) stained with Topro. **(D)** Representative image of *R4-Gal4;domeMESO GFP < UAS Tra*^*F*^ feminized female stained with ToPro. (D’) Representative image of *R4-Gal4;domeMESO GFP < UAS Tra*^*F*^ feminized male stained with ToPro. **(E-F)** Raw cell counts for total number of crystal cells, stained with Hnt. Quantification using a two-way ANOVA, post-hoc Tukey’s test, for: *R4-Gal4* control (n = 22), *R4-Gal4* feminized females (n = 32), *UAS Tra*^*F*^ control (n = 18), *R4-Gal4* control males (n = 25), *R4-Gal4* feminized males (n = 31), *UAS Tra*^*F*^ control males (n = 24). The genotype:sex interaction constant was no significant (p = 0.7910). (G, G’) Representative image of *R4-Gal4* control female (G) and male (G’) stained with Hnt. **(H)** Representative image of *R4-Gal4;domeMESO GFP x UAS Tra*^*F*^ feminized female stained with Hnt. (H’) Representative image of *R4-Gal4;domeMESO GFP x UAS Tra*^*F*^ feminized male stained with Hnt. **(I-J)** Raw cell counts for total number of progenitors, expressing GFP in domeMESO+ cells. Quantification using a two-way ANOVA, post-hoc Tukey’s test, for: *R4-Gal4* control females (n = 22), *R4-Gal4* feminized females (n = 32), *UAS Tra*^*F*^ control females(n = 27), *R4-Gal4* control males (n = 21), *R4-Gal4* feminized males (n = 31), *UAS Tra*^*F*^ control males(n = 21). The genotype:sex interaction constant was significant (p = 0.0045). (K, K’) Representative image of *R4-Gal4* control female (K) and male (K’), expressing GFP in domeMESO+ cells. **(L)** Representative image of *R4-Gal4;domeMESO GFP x UAS Tra*^*F*^ feminized female, expressing GFP in domeMESO+ cells. (L’) Representative image of *R4-Gal4;domeMESO GFP x UAS Tra*^*F*^ feminized male, expressing GFP in domeMESO+ cells.**** indicates P < 0.0001, *** indicates P < 0.001, ** indicates P < 0.01, * indicates P < 0.05, ns (non-significant) indicates P > 0.05. Error bars indicate 95% CI.

Our control experiments showed that in the genetic background of *R4*-Gal4, the driver we employed for expression in the fat body, the overall size of the lymph gland was smaller than other backgrounds (Fig 4A). However, since this effect was seen in both males and females it did not impact our interpretation. In particular, we found that feminization of the fat body had no significant impact on size differences between male and female lymph glands (Fig 4A-4D and Table 1). In contrast to what was observed for overall organ size, crystal cell numbers were higher in lymph glands of the experimental group compared to Tra^F^ and *R4*-Gal4 controls for both males and females. Nonetheless, despite this increase, we found no significant impact on sex differences in crystal cell numbers upon fat body feminization, as the increase was observed in both males and females (Fig 4E-4H and Table 1). When progenitor numbers were analysed, we observed a statistically significant increase in their number in the background of the Tra^F^ transgene (Fig 4I). Nonetheless, there was no clear impact on sex differences in progenitor numbers (Fig 4I-4L and Table 1). Taken as a whole, our data and statistical analysis supported the interpretation that sex differences in overall size, progenitor number, and crystal cell numbers were not mediated by the fat body.

### We find no evidence that sex differences in the lymph gland are mediated by the core progenitors

Since we did not uncover compelling evidence for a role of systemic signals from the CNS or the fat body in mediating sex differences in the lymph gland we shifted our focus to lymph gland specific mechanisms. We asked whether the identity of the blood progenitors themselves controlled sex differences. To test this hypothesis, we feminized the core blood progenitors in genetically male flies using the Tra^F^ transgene and studied how this impacted overall size as well as the size of individual cell populations in the lymph gland. As before, we compared experimental group flies to flies heterozygous for either the TraF construct (TraF/+) or the Gal4 driver (tep4-Gal4/+) and performed statistical analysis by determining the sex:genotype interaction constant (Table 1). While we noted that the magnitude of sex differences in lymph gland parameters in the *tep4*-Gal4 control was not as large as in other strains, this was not unexpected given known effects of genetic background on sex difference in size-related traits [67]. Because we still observed a strong female bias in these parameters in tep4-Gal4, we went ahead and performed our analysis as with other strains.

**Fig 5:**
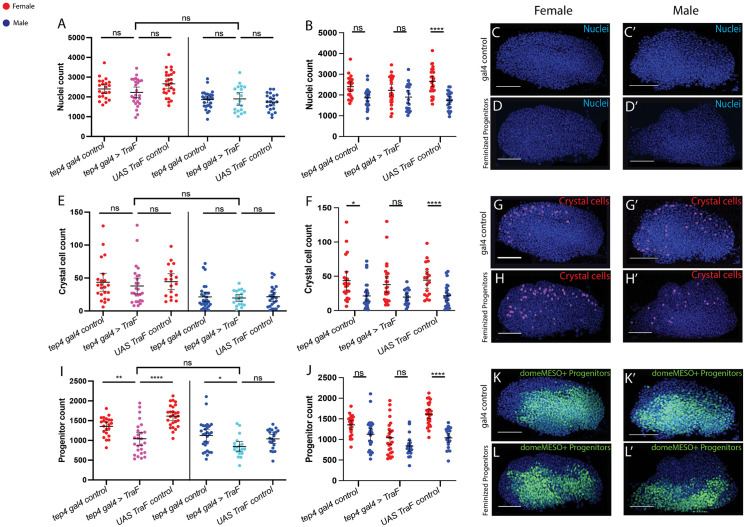
Feminizing the progenitors. **(A-B)** Raw cell counts for total number of nuclei, stained with ToPro. Quantification using a two-way ANOVA, post-hoc Tukey’s test, for: *tep4-Gal4* control females (n = 22), *tep4-Gal4* feminized females (n = 26), *UAS Tra*^*F*^ control females (n = 27), *tep4-Gal4* control males (n = 28), *tep4-Gal4* feminized males (n = 19), *UAS Tra*^*F*^ control males (n = 21). The genotype:sex interaction constant was significant (p = 0.0379). (C, C’) Representative image of *tep4-Gal4* control female (C) and male (C’) stained with Topro. (D, D’) Representative image of *tep4-Gal4;domeMESO GFP < UAS Tra*^*F*^ feminized female (D) and male (D’) stained with ToPro. **(E-F)** Raw cell counts for total number of crystal cells, stained with Hnt. Quantification using a two-way ANOVA, post-hoc Tukey’s test, for: *tep4-Gal4* control females (n = 22), *tep4-Gal4* feminized females (n = 26), *UAS Tra*^*F*^ control females (n = 18), *tep4-Gal4* control males (n = 28), *tep4-Gal4* feminized males (n = 19), *UAS Tra*^*F*^ control males (n = 24). The genotype:sex interaction constant was not significant (p = 0.8740). **(G)** Representative image of *tep4-Gal4* control female stained with Hnt. (G’) Representative image of *tep4gal4* control male stained with Hnt. **(H)** Representative image of *tep4-Gal4;domeMESO GFP x UAS Tra*^*F*^ feminized female stained with Hnt. (H’) Representative image of *tep4-Gal4;domeMESO GFP x UAS Tra*^*F*^ feminized male stained with Hnt. **(I-J)** Raw cell counts for total number of progenitors, expressing GFP in domeMESO+ cells. Quantification using a two-way ANOVA, post-hoc Tukey’s test, for: *tep4-Gal4* control females (n = 22), *tep4-Gal4* feminized females (n = 26), *UAS Tra*^*F*^ control females (n = 27), t*ep4-Gal4* control males (n = 28), *tep4-Gal4* feminized males (n = 19), *UAS Tra*^*F*^ control males (n = 21). The genotype:sex interaction constant was significant (p = 0.0069) **(K)** Representative image of *tep4-Gal4* control female, expressing GFP in domeMESO+ cells. (K’) Representative image of *tep4-Gal4* control male, expressing GFP in domeMESO+ cells. **(L)** Representative image of *tep4-Gal4;domeMESO GFP x UAS Tra*^*F*^ feminized female, expressing GFP in domeMESO+ cells. (L’) Representative image of *tep4-Gal4;domeMESO GFP x UAS Tra*^*F*^ feminized male, expressing GFP in domeMESO+ cells. **** indicates P < 0.0001, *** indicates P < 0.001, ** indicates P < 0.01, * indicates P < 0.05, ns (non-significant) indicates P > 0.05. Error bars indicate 95% CI.

Our analysis revealed significant values for sex:genotype interaction constant for the overall lymph size as well as for progenitor numbers but not for crystal cell numbers (Fig 5A-5D and 5E-5H, respectively; Table 1). However, a closer look revealed that changing the sexual identity of core blood progenitors in genetically male flies did not in fact significantly impact lymph gland size (Fig 5A). Specifically, we observe no differences from controls in terms of lymph gland size in either feminized males, or females overexpressing Tra^F^. This meant we could not draw any relevant conclusions from these experiments. In the case of the core progenitors, the change in sex differences was the result of a decrease in total progenitor cell counts in females with core progenitor-specific Tra^F^ expression, while there was no significant impact on progenitor numbers in feminized males (Fig 5I-5L and Table 1). Since the elimination of sex differences resulted from changes in the number of progenitors in females, rather than from feminization of male progenitors, the results of this experiment are difficult to interpret conclusively. To confirm these findings, we repeated this set of experiments using a more general progenitor driver, *domeMESO gal4*. Unlike the *tep4-*gal4 driver, which is restricted to core progenitors, the *domeMESO gal4* construct allowed us to feminize the entire progenitor pool in males. The results of this analysis (S6 Fig) were very similar to, and confirmed the results obtained with the *tep4gal4* driver. Taken as a whole, our data does not conclusively support or disprove an interpretation where feminizing lymph gland core progenitors impacts lymph gland size, crystal number or total progenitor numbers.

### Some sex differences in the lymph gland are mediated by the PSC

**Fig 6:**
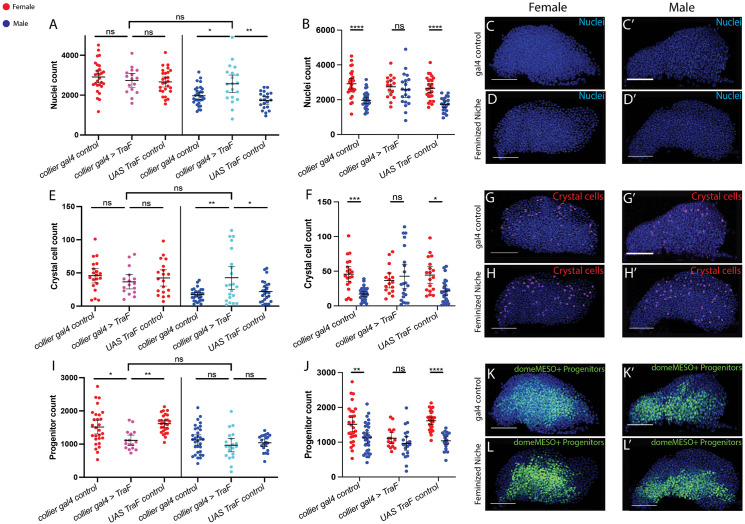
Feminizing the PSC. **(A-B)** Raw cell counts for total number of nuclei, stained with ToPro. Quantification using a two-way ANOVA, post-hoc Tukey’s test, for: *collier-Gal4* control females (n = 28), *collier-Gal4* feminized females (n = 16), *UAS Tra*^*F*^ control females (n = 27), *collier-Gal4* control males(n = 31), *collier-Gal4* feminized males(n = 20), *UAS Tra*^*F*^ control males (n = 21). The genotype:sex interaction constant was significant (p = 0.0139). (C, C’) Representative image of *collier-Gal4* control female (C) and male (C’) stained with Topro. **(D)** Representative image of *collier-Gal4;domeMESO GFP < UAS Tra*^*F*^ feminized female stained with ToPro. (D’) Representative image of *collier-Gal4;domeMESO GFP < UAS Tra*^*F*^ feminized male stained with ToPro. **(E-F)** Raw cell counts for total number of crystal cells, stained with Hnt. Quantification using a two-way ANOVA, post-hoc Tukey’s test, for: *collier-Gal4* control females (n = 20), *collier-Gal4* feminized females (n = 16), *UAS Tra*^*F*^ control females (n = 18), *collier-Gal4* control males (n = 29), *collier-Gal4* feminized males (n = 21), *UAS Tra*^*F*^ control males (n = 24). The genotype:sex interaction constant was significant (p = 0.0022). (G, G’) Representative image of *collier-Gal4* control female (G) and male (G’) stained with Hnt. **(H)** Representative image of *collier-Gal4;domeMESO GFP x UAS Tra*^*F*^ feminized female stained with Hnt. (H’) Representative image of *collier-Gal4;domeMESO GFP x UAS Tra*^*F*^ feminized male stained with Hnt. **(I-J)** Raw cell counts for total number of progenitors, expressing GFP in domeMESO+ cells. Quantification using a two-way ANOVA, post-hoc Tukey’s test, for: *collier-Gal4* control females (n = 28), *collier-Gal4* feminized females (n = 16), *UAS Tra*^*F*^ control females (n = 27), *collier-Gal4* control males (n = 31), *collier-Gal4* feminized males (n = 20), *UAS Tra*^*F*^ control males (n = 21) and *collier-Gal4* feminized males (p = 0.9910). The genotype:sex interaction constant was not significant (p = 0.0529). (K, K’) Representative image of *collier-Gal4* control female (K) and male (K’), expressing GFP in domeMESO+ cells. **(L)** Representative image of *collier-Gal4;domeMESO GFP x UAS Tra*^*F*^ feminized female, expressing GFP in domeMESO+ cells. (L’) Representative image of *collier-Gal4;domeMESO GFP x UAS Tra*^*F*^ feminized male, expressing GFP in domeMESO+ cells.**** indicates P < 0.0001, *** indicates P < 0.001, ** indicates P < 0.01, * indicates P < 0.05, ns (non-significant) indicates P > 0.05. Error bar indicates 95% CI.

The PSC has an established role in orchestrating the behaviour of different cell types in the lymph gland and is therefore a candidate for mediating sex differences [39–41,70]. Intriguingly, feminization of the PSC in males, using *collier*-Gal4, induced an increase in the number of cells in the lymph gland which led, for the most part, to the elimination of sex differences in size between females and PSC-feminized males (Fig 6A-6D and Table 1). A similar effect was seen for crystal cell numbers, which were higher in PSC-feminized males and resembled control females (Fig 6E-6H and Table 1). Thus, the sex difference in both overall lymph gland size and crystal cell number was eliminated due to a male-specific increase in these parameters with Tra^F^ expression (sex:genotype interaction p = 0.0139 and 0.0022, respectively; two-way ANOVA with post-hoc Tukey’s test).

In contrast to overall size or crystal cell numbers, there was a significant decrease in progenitor cell counts in females with no effect in males (Fig 6I-6L). This effect was similar between the sexes as the sex:genotype interaction was not significant (p = 0.0529; two-way ANOVA). Nonetheless, our data and statistical analysis did support the interpretation that sex differences in overall lymph gland size and crystal cell numbers were mediated, at least in part, by the PSC.

### Sex differences in crystal cell number are mediated by insulin signaling in the PSC

**Fig 7:**
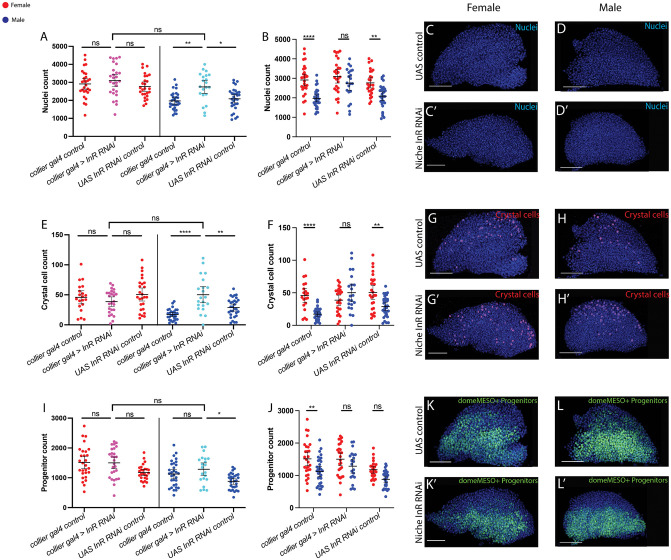
Knocking down insulin signaling in the PSC. **(A-B)** Raw cell counts for total number of nuclei, stained with ToPro. Quantification using a two-way ANOVA, post-hoc Tukey’s test, for: *collier-Gal4* control females (n = 28), *collier-Gal4>InR RNAi* females (n = 27), *UAS InR RNAi* females (n = 26), *collier-Gal4* control males (n = 31), *collier-Gal4>InR RNAi* males (n = 21), *UAS InR RNAi* control males (n = 30). The sex:genotype interaction constant was not significant (p = 0.0931). **(C)** Representative image of *+> UAS InR RNAi* control females stained with ToPro. (C’) Representative image of *collier-Gal4 > UAS InR RNAi* female stained with ToPro. **(D)** Representative image of *+> UAS InR RNAi* control male stained with ToPro, (D’) Representative image of *collier-Gal4 > UAS InR RNAi* male stained with ToPro. **(E-F)** Raw cell counts for total number of crystal cells, stained with Hnt. A two-way ANOVA, post-hoc Tukey’s test, for: *collier-Gal4* control females (n = 20), *collier-Gal4>InR RNAi* females (n = 25), *UAS InR RNAi* females (n = 26), *collier-Gal4* control males (n = 30), *collier-Gal4>InR RNAi* males (n = 21), *UAS InR RNAi* control males (n = 30). The sex:genotype interaction constant was significant (p < 0.0001). **(G)** Representative image of *+> UAS InR RNAi* control females stained with Hnt. (G’) Representative image of *collier-Gal4 > UAS InR RNAi* female stained with Hnt. **(H)** Representative image of *+> UAS InR RNAi* control male stained with Hnt (H’) Representative image of *collier-Gal4 > UAS InR RNAi* male stained with Hnt. **(I-J)** Raw cell counts for total number of progenitors, expressing GFP in domeMESO+ cells. A two-way ANOVA, post-hoc Tukey’s test, for: *collier-Gal4* control females (n = 28), *collier-Gal4>InR RNAi* females (n = 27), *UAS InR RNAi* females (n = 26), *collier-Gal4* control males (n = 31), *collier-Gal4>InR RNAi* males (n = 21), *UAS InR RNAi* control males (n = 30). The genotype:sex interaction constant was not significant (p = 0.5419). **(K)** Representative image of *+> UAS InR RNAi* control females expressing GFP in domeMESO+ cells. (K’) Representative image of *collier-Gal4 > UAS InR RNAi* female expressing GFP in domeMESO+ cells. **(L)** Representative image of *+> UAS InR RNAi* control male expressing GFP in domeMESO+ cells. (L’) Representative image of *collier gal4 > UAS InR RNAi* male expressing GFP in domeMESO+ cells.**** indicates P < 0.0001, *** indicates P < 0.001, ** indicates P < 0.01, * indicates P < 0.05, ns (non-significant) indicates P > 0.05. Error bar indicates 95% CI.

**Fig 8:**
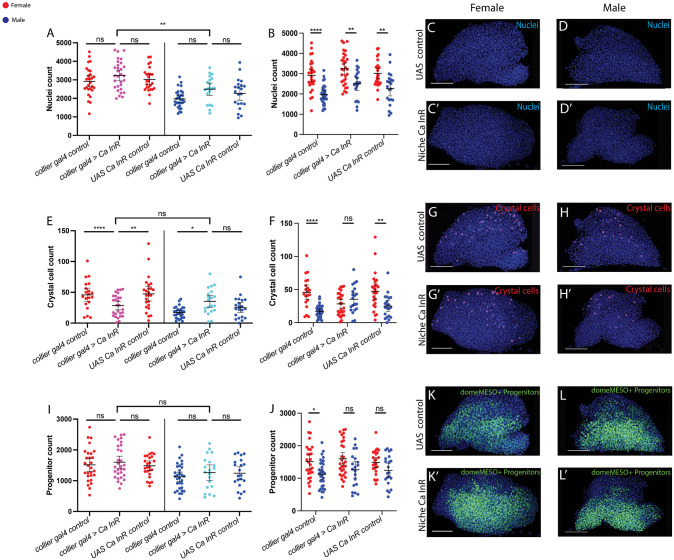
Constitutively activating insulin in the PSC. (A-B) Raw cell counts for total number of nuclei, stained with ToPro. Quantification using a two-way ANOVA, post-hoc Tukey’s test, for: *collier-Gal4* control females (n = 28), *collier-Gal4 > Ca InR* females (n = 30), *UAS Ca InR* control females (n = 25), *collier-Gal4* control males (n = 31), *collier-Gal4 > Ca InR* males (n = 20), *UAS Ca InR* control males (n = 21). The genotype:sex interaction constant was no significant (p = 0.6852). (C) Representative image of +> *UAS Ca InR* control females stained with ToPro. (C’) Representative image of *collier-Gal4 > UAS Ca InR* female stained with ToPro. (D) Representative image of +> *UAS Ca InR* control male stained with ToPro, (D’) Representative image of *collier-Gal4 > UAS Ca InR* male stained with ToPro. (E-F) Raw cell counts for total number of crystal cells, stained with Hnt. Quantification using a two-way ANOVA, post-hoc Tukey’s test, for: *collier-Gal4* control females (n = 20), *collier-Gal4 > Ca InR* females (n = 27), *UAS Ca InR* females (n = 25), *collier-Gal4* control males (n = 30), *collier-Gal4 > Ca InR* males (n = 20), *UAS Ca InR* control males (n = 21). The genotype:sex interaction constant was significant (p < 0.0001). (G) Representative image of +> *UAS Ca InR* control females stained with Hnt. (G’) Representative image of *collier-Gal4 > UAS Ca InR* female stained with Hnt. (H) Representative image of +> *UAS Ca InR* control male stained with Hnt (H’) Representative image of *collier-Gal4 > UAS Ca InR* male stained with Hnt. (I-J) Raw cell counts for total number of progenitors, expressing GFP in domeMESO+ cells. Quantification using a two-way ANOVA, post-hoc Tukey’s test, for: *collier-Gal4* control females (n = 28), *collier-Gal4 > Ca InR* females (n = 30), *UAS Ca InR* control females (n = 25), *collier-Gal4* control males (n = 31), *collier-Gal4 > Ca InR* males (n = 20), *UAS Ca InR* control males (n = 21). The genotype:sex interaction constant was not significant (p = 0.7455). (K) Representative image of +> *UAS Ca InR* control females expressing GFP in domeMESO+ cells. (K’) Representative image of *collier-Gal4 > UAS Ca InR* female expressing GFP in domeMESO+ cells. (L) Representative image of +> *UAS Ca InR* control male expressing GFP in domeMESO+ cells. (L’) Representative image of *collier-Gal4 > UAS Ca InR* male expressing GFP in domeMESO+ cells.**** indicates P < 0.0001, *** indicates P < 0.001, ** indicates P < 0.01, * indicates P < 0.05, ns (non-significant) indicates P > 0.05. Error bar indicates 95% CI.

Insulin signaling is known to be an important mediator of sex differences in size and tissue growth [71–74]. Insulin signaling is also known to be active in the PSC in the lymph gland to regulate hematopoiesis [50,51]. Based on these observations we hypothesized that insulin signaling, in the PSC, was important in mediating sex differences in the lymph gland. For these experiments we used *collier*-Gal4 to express either an RNAi to knock down the insulin receptor (Fig 7) or a constitutively active version of the insulin receptor (Fig 8). Both of the lines we used have been used repeatedly in the past and their effectiveness at modifying InR activity is well established [75].

We found that PSC-specific reduction in insulin signaling by expression of InR-RNAi led to an increase in the number of cells in male lymph glands, but not female lymph glands (Fig 7A-7D). However, the magnitude of the effect was not statistically significant between the sexes (sex:genotype p = 0.0931; two-way ANOVA; Table 2). In comparison, crystal cell numbers exhibited a more dramatic effect, as their number was greater in experimental group males in which InR was knocked down in the PSC than in females of comparable genotype (Fig 7E-7H). Importantly, we observed a significant sex:genotype interaction and the magnitude of the effect was greater in males than in females, which eliminated the sex difference in this trait (sex:genotype p < 0.0001, Table 2). Analysis of progenitor numbers following InR knockdown in the PSC did not provide statistically significant support for changes in sex differences in this trait (Fig 7I-7L) (sex:genotype p = 0.5789; Table 2).

**Table 2:** Interaction constants for insulin modulation. Sex:Genotype interaction constants derived from two-way ANOVA, post-hoc Tukey’s test performed on data from Figs 7-8 (see methods).

	Nuclei	Crystal cells	Progenitors
InR RNAi (Fig 7)	0.0931	<0.0001*	0.5789
Ca InR (Fig 8)	0.6852	<0.0001*	0.7455

* denotes a significant interaction constant (p < 0.05).

Analysis of PSC-specific insulin pathway activation via expression of Ca-InR revealed a similar trend. PSC-specific expression of Ca-InR did not greatly alter the number of cells in either male or female lymph glands and consequently there was no statistically significant impact on sex differences in organ size between females and males (Fig 8A-8D) (sex:genotype p = 0.6852; Table 2). In contrast, crystal cell numbers were decreased by PSC specific activation of insulin signaling in females, which resulted in a statistically significant impact leading to an elimination of sex differences (Fig 8E-8H) (sex:genotype p < 0.0001; Table 2). Similar to overall lymph gland size, progenitor numbers were not impacted by PSC specific insulin pathway activation in either males or females and consequently there did not appear to be a significant reduction in sex differences (Fig 8I-8L) (sex:genotype p = 0.7455; Table 2).

Based on the two sets of experiments, utilizing downregulation or hyperactivation of the insulin receptor in the PSC we do not find conclusive support for a function of the insulin pathway in the PSC in mediating sex differences in overall lymph gland size or progenitor numbers. However, there was robust statistical support for the finding that the insulin pathway in the PSC mediates sex differences in crystal cell number. We note that since we did not assess InR expression levels upon Tra^F^ overexpression in the PSC it is possible that pathways other than insulin may contribute to sex differences in crystal cell number. In this regard it is encouraging to that there is a similar fold-change in crystal cell number (~1.75) upon either Tra^F^ overexpression or upon InR knockdown in the PSC which argues that the Insulin pathway is a major contributor to sex differences. Nonetheless, further evidence confirming a change in signaling downstream of InR-RNAi and Ca-InR expression would be needed to substantiate this conclusion further.

### Sex-differences are observed in the cellular immune response following infection

**Fig 9:**
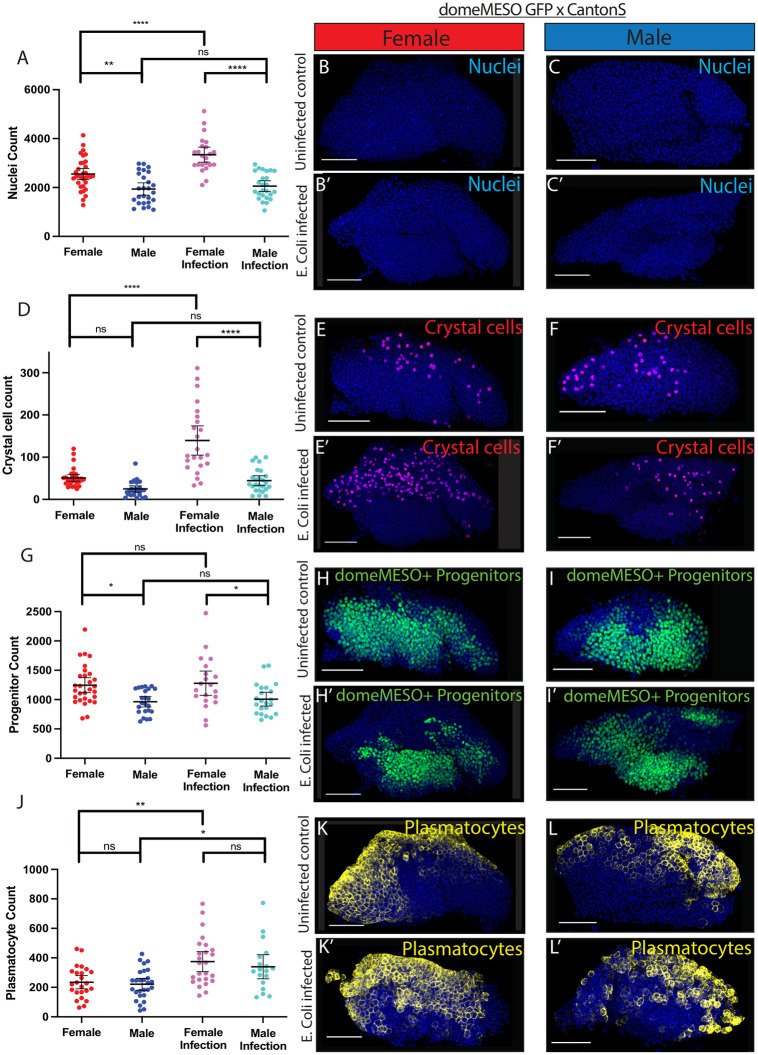
Infection with *E. Coli.* (A) Raw cell counts for total number of nuclei, stained with ToPro, in *domeMESO GFP x CantonS* flies both uninfected (female n = 33, male n = 26) and infected with *E.coli* bacteria (female n = 23, male n = 25). Sex:treatment interaction constant was significant (p = 0.0076). (B, B’) Representative image of an uninfected (B) and infected (B’) female lymph gland stained with ToPro. (C, C’) Representative image of uninfected (C) and infected (C’) male lymph gland stained with ToPro. (D) Raw cell counts for total number of crystal cells in *domeMESO GFP x CantonS* flies both uninfected (female n = 33, male n = 26) and infected with *E.coli* bacteria (female n = 23, male n = 25). Sex:treatment interaction constant was significant (p < 0.0001). (E, E’) Representative image of an uninfected (E) and infected (E’) female lymph gland stained with Hnt. (F, F’) Representative image of an uninfected (F) and infected (F’) male lymph gland stained with Hnt. (G) Raw cell counts for total number of progenitors, expressing GFP in domeMESO+ cells, in *domeMESO GFP x CantonS* flies both uninfected (female n = 29, male n = 23) and infected with *E.coli* bacteria (female n = 20, male n = 22). Sex:treatment interaction constant was not significant (p = 0.9293). (H, H’) Representative image of an uninfected (H) and infected (H’) female lymph gland expressing GFP in domeMESO+ cells. (I, I’) Representative image of an uninfected male (I) and infected (I’) lymph gland expressing GFP in domeMESO+ cells. (J) Raw cell counts for total number of plasmatocytes, stained with P1, in *domeMESO GFP x CantonS* flies both uninfected (female n = 24, male n = 27) and infected with *E.coli* bacteria (female n = 25, male n = 18). Sex:treatment interaction constant was not significant (p = 0.7160). (K, K’) Representative image of an uninfected (K) and infected (K’) female lymph gland stained with P1. (L, L’) Representative image of an uninfected (L) and infected (L’) male lymph gland stained with P1. All quantification done using a two-way ANOVA, post-hoc Tukey’s test. **** indicates P< < 0.0001, *** indicates P< < 0.001, ** indicates P < 0.01, * indicates P < 0.05, ns (non-significant) indicates P > 0.05. Error bar indicates 95% CI.

**Fig 10:**
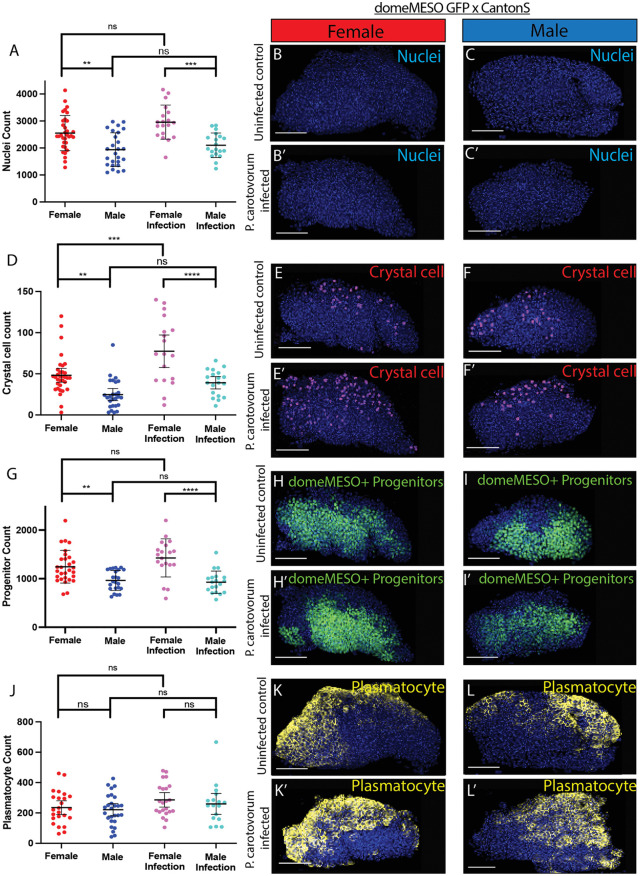
Infection with *P.carotovorum.* **(A)** Raw cell counts for total number of nuclei, stained with ToPro, in *domeMESO GFP x CantonS* flies both uninfected (female n = 33, male n = 26) and infected with *P. carotovorum* bacteria (female n = 19, male n = 19). Sex:treatment interaction constant was not significant (p = 0.3380) (B, B’) Representative image of an uninfected (B) and infected (B’) female lymph gland stained with ToPro. **(C)** Representative image of uninfected (C) and infected (C’) male lymph gland stained with ToPro. **(D)** Raw cell counts for total number of crystal cells, stained with Hnt, in *domeMESO GFP x CantonS* flies both uninfected (female n = 33, male n = 26) and infected with *P.carotovorum* bacteria (female n = 19, male n = 19). Sex:treatment interaction constant was not significant (p = 0.1627). (E, E’) Representative image of an uninfected (E) and infected (E’) female lymph gland stained with Hnt. (F, F’) Representative image of an uninfected (F) and infected (F’) male lymph gland stained with Hnt. **(G)**Raw cell counts for total number of progenitors, expressing GFP in domeMESO+ cells, in *domeMESO GFP x CantonS* flies both uninfected (female n = 29, male n = 23) and infected with *P. carotovorum* bacteria (female n = 19, male n = 19). Sex:treatment interaction constant was not significant (p = 0.0981). (H, H’) Representative image of an uninfected (H) and infected (H’) female lymph gland expressing GFP in domeMESO+ cells. (I, I’) Representative image of an uninfected (I) and infected (I’) male lymph gland expressing GFP in domeMESO+ cells. **(J)** Raw cell counts for total number of plasmatocytes, stained with P1, in *domeMESO GFP x CantonS* flies both uninfected (female n = 24, male n = 27) and infected with *P. carotovorum* bacteria (female n = 22, male n = 17). Sex:treatment interaction constant was significant (p = 0.7884). **(K)** Representative image of an uninfected female lymph gland stained with P1. (K’) Representative image of an infected female lymph gland stained with P1. **(L)** Representative image of an uninfected male lymph gland stained with P1. (L’) Representative image of an infected male lymph gland stained with P1. All quantification done using a one-way ANOVA, post-hoc Tukey’s test. **** indicates P < 0.0001, *** indicates P < 0.001, ** indicates P < 0.01, * indicates P < 0.05, ns (non-significant) indicates P > 0.05. Error bar indicates 95% CI.

Previous studies in adult *Drosophila* identified sex-specific differences in the immune response to infection that led to variation in survival rates. Intriguingly, these differences appeared to vary substantially based on the strain of bacteria used for infection [31]. We analysed the cellular immune response in males and females of wild-type (Canton-S) exposed to two different strains of bacteria that are known to activate the immune response in flies, *Escherichia coli (E. coli)* and *Pectobacterium carotovorum* (*P. carotovorum*; previously known as *Erwinia carotovora carotovora*, or *Ecc15)* [73,75](see methods). These strains were chosen because they have been shown to act in a sex-specific manner to control behaviour in adult flies [31,76–78]. We applied a feeding-based infection protocol, based on previous findings in Sawala et al [79] showing that feeding amount was proportional to body size in males and females meaning their relative food consumption per weight was similar. Infection by feeding with *E.coli* showed a sex-specific increase in organ size and crystal cell count in females, but not in males (Fig 9A-9C and 9D-9F and Table 3). In comparison, neither sex showed an increase in progenitor count (Fig 9G-9I and Table 3), while both sexes showed an increase in plasmatocyte count upon infection (Fig 9J-9L and Table 3). Infection with P. carotovorum resulted in a significant increase in crystal cell numbers in females but not in males (Fig 10D-10F and Table 3). We found our data were not normally-distributed and consequently used non-parametric tests to determine whether there was a sex:treatment interaction for the effect of Ecc infection on crystal cell number. Despite the fact that there is an apparent female-biased effect, our non-parametric test still did not detect a significant sex:treatment interaction. Specifically, in two types of non-parametric two way ANOVA, the Schreier-Ray-Hare test and an Aligned Rank Transform test, we obtained P values consistent with an insignificant interaction (p = 0.1491 and p = 0.599, respectively). Because the sex:treatment interaction was not significant, potentially due to a variation in the number of female crystal cells, we cannot conclude that there was a sex-biased effect of the infection on crystal cell number. Further experiments will therefore be important in resolving this question. In addition, overall organ size, progenitor numbers, and plasmatocyte numbers were not impacted by infection in either males or females (Fig 10A-10C, 10G-10I, and 10J-10L and Table 3). Taken together these data show that for *E. coli,* organ size and crystal cell production following infection was differentially regulated between male and female fly larvae. Moreover, analysis of infection with *P. carotovorum* showed that the magnitude or even the existence of sex-differences in the response to infection may vary depending on the bacterial strain used.

**Table 3:** Interaction constants for infection. Sex:treatment interaction constants derived from two-way ANOVA, post-hoc Tukey’s test performed on data from Figs 9-10 (see methods).

	Nuclei	Crystal cells	Progenitors	Plasmatocytes
*E. coli*	0.0076*	<0.0001*	0.9293	0.7160
P. carotovorum	0.3380	0.1627	0.0981	0.7884

* denotes a significant interaction constant (p < 0.05).

## Discussion

Our work establishes the *Drosophila* larva as a model for analysing sex-based differences in cellular immunity. In this regard we build upon an increasing body of evidence that multiple aspects of innate immunity in adult *Drosophila* exhibit striking sex-based variation [29–33,77–80]. We find that sex impacts the various cell types found in the lymph gland to different extents under both homeostatic or infection conditions, and that there are distinct mechanisms that control how sex differences are established in the various cell types. This supports a more nuanced and complex role for sex in regulating hematopoiesis beyond a simple generalized increased tissue size and cell number [74,81]. This idea is further supported by single-cell RNA-Seq that uncovered pervasive and wide-ranging sex-differences at the level of gene expression across cell types in the lymph gland. Moreover, we found that the PSC, and more specifically insulin signaling from the PSC, mediated some sex differences but that there are other mechanisms in place since we were not able to mechanistically account for all the variance between males and females. Finally, in line with previous findings of sex-differences in the ability to survive infection in adults [27,29–33] we find sex differences in the production of cellular immune response components in larva that was elicited by bacterial infection. Moreover, and consistent with previous findings [27,29–33], sex differences in the cellular immune response differed depending on the bacterial strain used for infection, indicating a complex relationship between sex, immunity, and infection. Taken together our work not only functions as an initial characterization of baseline sex-differences in fly hematopoiesis and in cellular immunity, but also identifies important areas for future exploration.

Our analysis of lymph gland single-cell RNA-Seq (scRNA-Seq) data shows significant differences in gene expression across multiple male and female cell types. These differences are not solely confined to genes involved in sex determination and X chromosome dosage compensation. We also observed sex-based expression differences in genes that have been implicated in hematopoiesis and blood cell function. Therefore, we hypothesize that some of the genes we found to be enriched in male or female cell types may be involved in generating the sex-specific hematopoietic phenotypes observed in lymph glands in this study. For example, we found that manipulating insulin signaling in the PSC in males, but not females, leads to changes in crystal cell differentiation. Intriguingly, crystal cells exhibit a more pronounced level of sex-differences compared to other cell types in the lymph glands, beyond what can be simply accounted for by body size. With that added context it is possible that the sex-based differences in crystal cell numbers represent a unique difference in developmental outcome rather than a difference that is purely based on body size. These data also align well with the scRNA-Seq data, which shows that the insulin-like peptide dILP-6/Ilp6 is specifically enriched in male PSC cells and male crystal cells (Fig 2B). Future study is required to test if enrichment of dILP-6/Ilp6 expression in male PSC cells is an underlying mechanism contributing to this phenotype. Moreover, gene expression differences in our scRNA-Seq data suggest interesting hypotheses about the potential causes underlying the different infection phenotypes observed in male and female lymph glands. For instance, we observed enrichment in the Toll and Imd signaling pathways in female progenitors, intermediate progenitors, and plasmatocytes compared to their male counterparts. These cell types can undergo cell division and serve as precursors for crystal cell differentiation [82,83–86] Further study is needed to understand whether this sex-specific difference in gene expression in these cell types is required for the increase in crystal cells and lymph gland size observed in females after infection.

Although the existence of sex differences in innate immunity in *Drosophila* adults is well established [27,29,31], it is not yet standard practice to consider sex as a variable in studies that focus on the larval lymph gland. Based on our findings, we believe there is a substantial argument that in the future it should become a standard practice to consider sex as a key variable for all phenotypic characterization of the lymph gland. Although the common practice in the field of normalizing the reported cell counts to lymph gland size would, in general, correct for sex-differences in size, it might not do so in all instances. Moreover, previous genetic experiments that allowed manipulations in only one sex, for example due to the availability of certain drivers only on the X-chromosome, may need to be revisited and assayed for both sexes. We therefore suggest that data should be reported separately for both sexes. Although some aspects of larval immunity, such as plasmatocyte numbers, may not vary greatly between males and females under normal conditions, differences might become apparent under specific experimental conditions. Our study did not aim to exhaustively characterize all sex-based differences in the lymph gland, which we suspect vary considerably based on the genetic background and experimental conditions. Rather our aim was to establish that sex differences are pervasive in the lymph gland and to provide some insight into the mechanisms that are responsible for these differences. We predict that as more researchers in the field separately report data for males and females for various lymph gland phenotypes, the number of documented sex differences will greatly expand. Moreover, the emergence of sex-differences, and exploring the possibility that differences already exist in embryonically derived hemocytes should prove an exciting area for future research.

Our analysis argues for an important role for the *Drosophila* lymph gland niche, the PSC, in at least a subset of sex differences. We did not find conclusive evidence for a role in sex differences for systemic signals that originate from the CNS and the fat body, or from local signals derived from the progenitors. A key limitation of these studies is the lack of completely specific drivers that only, with 100% specificity, drive expression in the tissue they are targeting (such as the CNS or Fat). Moreover, the drivers we use to express in different cell-populations in the lymph gland can be expressed outside of the lymph gland. For example, collier-Gal4 is expressed in the PSC of the lymph gland, but is also expressed in the CNS, wing disc pouch, and sporadically in the lymph gland posterior lobes [87].Coupled with experimental noise and the influence of genetic background this can make interpretation challenging. We therefore sought to be as rigorous as possible in how we interpreted the results and set a high threshold to accept an effect as real. In particular, the thresholds that needed to be met to deem a result as meaningful were ensuring both the multiple comparisons across genotypes as well as the sex:genotype interaction was statistically significant. This cautious approach means that with the availability of better tools in the future we may find more tissues or cell types in the lymph gland are involved in mediating sex-differences. Nonetheless, our results already suggest that juxtacrine signals or perhaps cell autonomous signals play an important mechanistic role in sex differences in hematopoiesis. However, this does not imply the absence of other known types of sex differences that have been demonstrated to exist in flies and that we did not assay for, such as differences in physiology or metabolism [81]. Instead, our work assayed cell numbers, a parameter which is most relevant for immune function. As the repertoire of known sex differences in hematopoiesis grows, which we predict will be the case once separating data by sex becomes the norm in the field, it will be valuable to revisit the role of systemic signals as these are likely to play a part in at least some of these additional processes. Moreover, finding the mechanisms that mediate sex differences in progenitor numbers, which at present remain unknown, will be an important goal of future work.

In adult *Drosophila*, there are intriguing sex-specific behavioral responses to the presence of bacteria. For example, female flies are less likely to lay eggs in the presence of high levels of bacteria, seen as a protective mechanism designed to avoid egg laying in an environment inhospitable to them [75]. In the larval stage it is less clear what functions sex differences may have. It could be that their role is to set up future differences in the adult, and/or it could be that there is emphasis on preventing infection that could become chronic and harm egg production. The possibility of sex differences in larva impacting the adult immune response is supported by noting that a large portion of adult hemocytes derive from the larval lymph gland [88]. The potential importance of preventing infection in females during larval stages is highlighted by studies showing that infection in adult female flies impacts egg production and/or fecundity [89,90].

Taken as a whole, our work highlights the complex interactions between sex, genotype, and the environment in controlling *Drosophila* hematopoiesis and immunity. Our phenotypic analysis shows substantial variation across genotypes and environmental conditions in the proportion of different cell populations present in the lymph gland. In some experiments this did not allow us to obtain conclusive findings. However, this variability could represent important individual differences in life history, growth conditions, and environmental conditions between larvae. By accounting for sex as a factor that impacts lymph gland phenotype, it should be possible to reduce variation introduced by mixing data from males and females. This consideration will improve reproducibility and make future analysis more precise and meaningful.

## Materials and methods

### Fly stocks and genetics

All *Drosophila* crosses were kept at 25°C and sustained in vials containing a standard cornmeal fly food (recipe from Bloomington *Drosophila* Stock Center). All lymph gland progenitors were labelled using fluorescent markers *tep4-gal4; UAS-GFP or domeMESO GFP*. Control larvae in Fig 1 are categorized as *w*^*1118*^ flies crossed to *tep4-gal4; UAS GFP*. Wildtype larvae in Figs 8-9 are categorized as CantonS flies crossed to *domeMESO GFP*.

Using the Gal4/UAS system [89], various tissues were feminized by overexpressing sex determination gene *transformer (*tra), a master regulator of female sexual identity and development [61–64,81]. The drivers used were *elav-gal4* (CNS) [91], *R4-gal4* (fat body) [92], *tep4-gal4* (lymph gland progenitors) [93], and *collier-gal4* (PSC cells) [94]. These drivers were crossed to *domeMESO GFP* for controls, and *UAS Tra*^*F*^*/CyoGFP;domeMESO GFP/TM6* for feminization experiments. A UAS control was also used by crossing *UAS Tra*^*F*^*/CyoGFP; domeMESO GFP/TM6* to *domeMESO GFP*.

To control for the possible genetic effects of the domeMesoGFP transgene we confirmed its inclusion had no impact on sex differences in the lymph gland (S7 Fig).

In order to constitutively activate or knockdown insulin receptors in the lymph gland, *Ca-InR* (RRID: BDSC_8250) and *InR RNAi* (RRID: BDSC_51518) were crossed to *domeMESO GFP* as a control, and crossed to PSC cell driver *collier-gal4*.

### Larval lymph gland dissections

Wandering third instar larval lymph glands were dissected in ice-cold 1X Phosphate Buffer Saline (PBS), and then fixed in 4% paraformaldehyde (PFA) for 15 minutes. Samples were then washed in 0.1% PTX (1X PBS with 0.1% Triton X [Thermofisher Scientific, BP 151100]) twice for 5 minutes each, then blocked with 16% Neat Goat Serum (NGS) (ab7481, abcam) for 15 minutes, before being incubated in a primary antibody overnight at 4°C. The following day, samples were washed again twice in 0.1% PTX for 5 minutes each, blocked in 16% NGS for 15 minutes, before being incubated in a secondary antibody for two hours at room temperature. Samples were washed three times in 0.1% PTX for 5 minutes each, and mounted in VECTASHIELD (Vector Laboratories, H-1000, RRID: AB_2336789) with TOPRO3 iodide (ThermoFisher Scientific, T3605; 1:500) in glass bottom mounting dishes (MatTek Corporation, 35 mm, P35G-0–14-C).

### Immunohistochemistry

The following primary antibodies were used and diluted in 0.1% PTX: mouse anti-hindsight (1:50, DSHB 1G9, RRID: AB_2617420), mouse anti-Antennapedia (1:25, DSHB 4C3, RRID: AB_528082). The following primary antibodies were used and diluted in 0.1% Tween 20 (1X PBS with 0.1% Tween 20 [Fisher BioReagents, BP337–500]): mouse anti- P1 (1:100, anti-NimC1, a kind gift from Dr. Istvan Ando, Hungarian Academy of Sciences, Hungary).

The following secondary antibody was used and diluted in 0.1% PTX (for Hnt and Antp staining) or 0.1% Tween 20 (for P1 staining): donkey anti-mouse Cy3 (1:400, Jackson Immunoresearch Laboratories, Code: 715-165-150, RRID: AB_2340813) and TOPRO3 iodide (1:800).

### Analysis of single-cell RNA-Seq data

Partek Flow software was used for single-cell sequencing analysis. The previously published single-cell data set was used, which includes the same initial data processing parameters for read trimming, quality control, alignment of reads to the Drosophila melanogaster reference genome r6.22, normalization and low input filtration, and exclusion of ribosomal RNA genes [95].

Principal component (PC) analysis (PCA) was completed prior to graph-based clustering. Based on the Scree plot, the first 20 PCs were selected to use as the input for clustering and data visualization tasks. Graph-based clustering was first performed with 50 nearest neighbors (NNs) and resolution (res) of 0.25–0.75 giving 6–9 clusters, with 7 clusters (res = 0.5-0.57) showing the most marked differences in gene expression between clusters. The data was visualized using UMAP with a local neighborhood size of 15, minimal distance of 0.1, Euclidean distance metric, and random generator seed of 0. We used established lymph gland marker genes to identify which cell type each of the graph-based clusters corresponds as done previously [82]. We determined sex by using the expression of two long non-coding RNAs (lncRNAs): lncRNA:roX1, lncRNA:roX2. Cells that are high for expression of both lncRNA:roX1 and lncRNA:roX2 were characterized as males, cells that were low for both were characterized as female, and cells that had high expression for only one of the lncRNAs were characterized as ambiguous and excluded from further analysis.

We used an ANOVA analysis to determine which genes were differentially expressed in males versus female cells. We set a threshold of 1.5-fold enrichment for genes to be considered differentially expressed and discarded genes which were enriched below that threshold. We also used a false-discovery rate (FDR) threshold of less than or equal to 0.0001, discarding any genes which were above that threshold. We took the remaining genes and thus had a list of differentially expressed genes (DEGs) for males and females (S1–S4 Files). We then performed a similar analysis comparing male and female cells in each of the graph-based clusters to identify sex differences in specific lymph gland cell types (S1–S4 Files).

We then compared the list of DEGs from each cluster to one another and to the DEGs for the lymph gland overall (S10 File). We used the multiple list comparator from molbiotools to compare the lists and generate Venn diagrams to see which genes were uniquely over-expressed in each cluster. We then used g:Profiler’s

g:GOSt tool [96] for gene-set enrichment analysis to find gene ontology terms, KEGG and WP pathways, or transcription factor binding sites associated with the DEGs in each cluster (S5–S8 Files). We used these terms to highlight DEGs of particular interest due to their pattern of enrichment in specific male or female cell types, their involvement in blood cell development or disease, and their conservation across species (S9 File).

### Infection

An ampicillin resistant, GFP expressing strain of *Escherichia coli* [93,94](a kind gift from Dr. Bret Finlay, The University of British Columbia, Vancouver, Canada) and *Pectobactorium carotovorum* (a kind gift from Dr. Edan Foley, University of Alberta, Edmonton, Canada) were grown in LB media and incubated at 37°C and 30°C respectively overnight. Second instar larvae were picked out of food, washed in ddH2O and 70% ethanol, then starved on a polydimethylsiloxane pad for 2 hours before being placed into 4g of fly food with either 200 µl of PBS (control) or 200µL of *E.coli* or *P. carotovorum* resuspended in PBS. Vials were then placed back in 25°C for 9 hours and dissected using the protocol described previously.

### Imaging and data analysis

All images were acquired on an Olympus FV1000 inverted confocal microscope, using a 40x lens. Image analysis was done using Olympus Fluoview (Ver.1.7c) and MATLAB scripts previously used in Khadiklar, 2017 [93]. Total number of nuclei, prohemocytes and plasmatocytes were determined using this Matlab script. Crystal cells and niche cells were counted manually using the cell counter plugin in ImageJ.

To correct for body size, average weight was taken for males and females of a genotype by collecting 10–12 third instar larvae, weighing them, and dividing total weight by number of larvae weighed. This weight, in milligrams, was then used to correct data points for body size (Fig 1F’-1J’).

Statistics were performed using GraphPad Prism. P values were determined using a two-tailed unpaired t-test, a one way ANOVA with multiple comparisons (Tukey’s test), or a two-way ANOVA multiple comparison (Tukey’s Test). **** indicates P < 0.0001, *** indicates P < 0.001, ** indicates P < 0.01, * indicates P < 0.05, ns (non-significant) indicates P > 0.05. The sample size and statistical method of each analysis were indicated in the figure legends.

A two-way ANOVA (Tukey’s test) was used to determine both p values in multiple comparisons, as well as an overall sex:genotype or sex:treatment interaction value. The multiple comparison significance are shown within the figure, with the exact p values and the interaction value shown in the figure legends.

## Supporting information

S1 Movie Three dimensional UMAP visualization of single-cell sequencing data showing cells from three biological replicate samples (21,157 total cells), each containing a mixed population of lymph gland primary lobes from 5 male and 6 female larvae. Graph based clusters defining lymph gland cell types (left panel) and expression of the male-specific genes lncRNA:roX1 (middle panel) and lncRNA:roX2 (right panel) visualized on the UMAP. PSC, posterior signaling center; MZ, medullary zone; IZ, intermediate progenitor; proPL, proplasmatocyte; PL, plasmatocyte; CC, crystal cell; X, mitotic cluster.
(MOV)

S1 File Anova analysis was used to determine fold change in the number of reads for each gene, comparing female cells to male cells, as distinguished by expression of roX1 and rox2. We set a threshold of 1.5 fold change, and <=0.0001 for the FDR step-up, discarding any genes which did not meet both thresholds.
(PDF)

S2 File Anova analysis was used to determine fold change in the number of reads for each gene in the MZ, comparing female cells to male cells, as distinguished by expression of roX1 and rox2. We set a threshold of 1.5 fold change, and <=0.0001 for the FDR step-up, discarding any genes which did not meet both thresholds.
(PDF)

S3 File Anova analysis was used to determine fold change in the number of reads for each gene in the crystal cells, comparing female cells to male cells, as distinguished by expression of roX1 and rox2. We set a threshold of 1.5 fold change, and <=0.0001 for the FDR step-up, discarding any genes which did not meet both thresholds.
(PDF)

S4 File Anova analysis was used to determine fold change in the number of reads for each gene in the PSC, comparing female cells to male cells, as distinguished by expression of roX1 and rox2. We set a threshold of 1.5 fold change, and <=0.0001 for the FDR step-up, discarding any genes which did not meet both thresholds.
(PDF)

S5 File Using g:Profilers’s g:GOSt tool for gene-set enrichment analysis we analyzed lists of unique DEGs from S4 File (see highlighted columns) to find gene ontology terms, pathways, and transcription factor targets that are enriched in each sex and cluster overall. (PDF)

S6 File Using g:Profilers’s g:GOSt tool for gene-set enrichment analysis we analyzed lists of unique DEGs from S4 File (see highlighted columns) to find gene ontology terms, pathways, and transcription factor targets that are enriched in each sex and cluster in the MZ. (PDF)

S7 File Using g:Profilers’s g:GOSt tool for gene-set enrichment analysis we analyzed lists of unique DEGs from S4 File (see highlighted columns) to find gene ontology terms, pathways, and transcription factor targets that are enriched in each sex and cluster in the crystal cells. (PDF)

S8 File Using g:Profilers’s g:GOSt tool for gene-set enrichment analysis we analyzed lists of unique DEGs from S4 File (see highlighted columns) to find gene ontology terms, pathways, and transcription factor targets that are enriched in each sex and cluster in the PSC. (PDF)

S9 File A selection of interesting sex-specific DEGs for further study. These genes were selected for being enriched in one cell type, being broadly conserved across species (including in humans in most cases), and having some connection to blood cell development or disease, or immune cell phenotypes. References are included for each gene detailing the known roles of these genes or their mammalian orthologs in blood cell development, disease, or phenotypes.
(PDF)

S10 File We used the multiple list comparator from molbiotools to compare DEG lists from Table 1 and determine which genes were uniquely overexpressed in each cluster. We ran this analysis both with and without the ‘overall’ category, which refers to the comparison of total male and female lymph gland cells.
(PDF)

S1 Table Total counts for sex determination genes. Total number of sex determination genes separated by males and females, as well as total counts per cell.
(PDF)

S1 Fig Differences between collier+ and Antp+ cell counts. Total cell count for both collier+ PSC cells (expressing GFP through *collier-gal4*) as well as Antp+ cells (stained with Antp antibody) in males and females. A significant difference was found in females between the two cell types (p = 0.0029). A significant difference was found in males between the two cell types (p = 0.0003).
(TIF)

S2 Fig Mean Intensity of *colliergal4* and *tep4gal4* Mean intensity of both collier+ and tep4+ cells measured for both males and females using ImageJ. (TIF)

S3 Fig Raw cell counts from main text Fig 1H-1L corrected by dividing each female data point by average total lymph gland nuclei count (main text Fig 1G) and dividing each male data point by average phenotype weight (main text Fig 1G). (A) Corrected number of PSC cells (Antp+) (female n = 64, male n = 59, p = 0.7819). (B) Corrected number of core progenitors (tep4+) (female n = 64, male n = 59, p = 0.9705). (C) Corrected number of crystal cells (Hnt+) (female n = 25, male n = 19, p = 0.0056). (D) Corrected number of plasmatocytes (P1+) (female n = 30, male n = 22, p = 0.4531). (E) Corrected number total progenitors (domeMESO+) (female = 24, male = 26, p = 0.6048). **** indicates P < 0.0001, *** indicates P < 0.001, ** indicates P < 0.01, * indicates P < 0.05, ns (non-significant) indicates P > 0.05. Error bars are 95% CI.
(TIF)

S4 Fig Crystal cell counts corrected for body and lymph gland size. (A) Raw cell counts for crystal cells in *w1118 x tep4GFP* male and female larvae show a significant difference (p < 0.0001). (B) Total cell counts for crystal cells in *w1118 x tep4GFP* male and female larvae, corrected for body size, show a significant difference (p = 0.0056). (C)Total cell counts for crystal cells in *w1118 x tep4GFP* male and female larvae, corrected for total lymph gland size (total nuclei count for each lobe corresponding to crystal cell number data point), show a significant difference (p = 0.0011).
(TIF)

S5 Fig Feminization with c2-gal4 (A-B) Raw cell counts for total number of nuclei, stained with ToPro. Quantification using a two-way ANOVA, post-hoc Tukey’s test, for: c2-Gal4 female control (n = 22), c2-Gal4 feminized females (n = 23), UAS TraF control females (n = 27), c2-Gal4 control males (n = 21),c2-Gal4 feminized males (n = 16), UAS TraF control males (n = 21). The genotype:sex interaction constant was not significant (p = 0.4208). (C-D) Raw cell counts for total number of crystal cells, stained with Hnt. Quantification using a two-way ANOVA, post-hoc Tukey’s test, for: c2-Gal4 control (n = 22), c2-Gal4 feminized females (n = 23), UAS TraF control (n = 18), c2-Gal4 control males (n = 21), c2-Gal4 feminized males (n = 16), UAS TraF control males (n = 24). The genotype:sex interaction constant was not significant (p = 0.7396). (E-F) Raw cell counts for total number of progenitors, expressing GFP in domeMESO+ cells. Quantification using a two-way ANOVA, post-hoc Tukey’s test, for: c2-Gal4 control females (n = 22), c2-Gal4 feminized females (n = 23), UAS TraF control females(n = 27), c2-Gal4 control males (n = 21), c2-Gal4 feminized males (n = 16), UAS TraF control males(n = 21). The genotype:sex interaction constant was not significant (p = 0.0590).
(TIF)

S6 Fig *domeMESO* *gal4* based feminization of progenitors. A) Raw cell counts for total number of nuclei. A 2 way ANOVA, Tukey’s test showed: no significance between *domeMESO gal4* control females and *domeMESOgal4* feminized females (p = 0.7209), a significant difference between *UAS TraF* control females and *domeMESOgal4* feminized females (p = 0.0005), no significance between *domeMESO* control males and *domeMESO* feminized males (p = 0.9949), and no significance between *UAS TraF* control males and *domeMESO* feminized males (p = 0.2093). The genotype:sex interaction constant was not significant (p = 0.1876). (B) Raw cell counts for total number of nuclei comparing females and males of each group. A 2 way ANOVA, Tukey’s test showed: no significant difference between *domeMESO gal4* control females and males (p = 0.4752), no significant difference between *domeMESO gal4* feminized females and males (p = 0.1425), and a significant difference between *UAS TraF* control females and males (p < 0.0001). (C) Raw cell counts for total number of crystal cells. A 2 way ANOVA, Tukey’s test showed: no significant difference between *domeMESO gal4* control females and *domeMESO gal4* feminized females (p = 0.2599),no significant difference between *UAS TraF* control females and *domeMESO gal4* feminized females(p > 0.9999), no significant difference between *domeMESO gal4* control males and *domeMESO gal4* feminized males (p = 0.2422), and no significant difference between *UAS TraF* control males and *domeMESO gal4* feminized males (p = 0.9452). The genotype:sex interaction constant was not significant (p = 0.7756). (D) Raw cell counts for total number of crystal cells comparing females and males of each group. A 2 way ANOVA, Tukey’s test showed: no significant difference between *domeMESO gal4* control females and males (p = 0.1782), no significant difference between *domeMESO gal4* feminized females and males (p = 0.3322), and a significant difference between *UAS TraF* control females and males (p = 0.0032). (E) Raw cell counts for total number of *domeMESO+* progenitors. A 2 way ANOVA, Tukey’s test showed: no significant difference between *domeMESO gal4* control females and *domeMESO gal4* feminized females (p > 0.9999), a significant difference between *UAS TraF* control females and *domeMESO gal4* feminized females (p < 0.0001), no significant difference between *domeMESO gal4* control males and *domeMESO gal4* feminized males (p = 0.9393), and a significant difference between *UAS TraF* control males and *domeMESO gal4* feminized males (p = 0.0001). The genotype:sex interaction constant was significant (p = 0.0252). (F) Raw cell counts for total number of *domeMESO+* progenitors comparing females and males of each group. A 2 way ANOVA, Tukey’s test showed: no significant difference between *domeMESO gal4* control females and males (p = 0.5104), no significant difference between *domeMESO gal4* feminized females and males(p = 0.1194), and a significant difference between *UAS TraF* control females and males (p < 0.0001).
(TIF)

S7 Fig Heterozygous/Homozygous domeMESOGFP. Heterozygous cross of UAS TraF; + x domeMESOGFP and homozygous cross of UAS TraF;domeMESOGFP x domeMESOGFP, separated by sex. (A) Raw total nuclei count of heterozygous females(n = 17) and homozygous females(n = 16) are shown to have no significant difference (p = 0.2208). (B) Raw total nuclei count of heterozygous males(n = 21) and homozygous males (n = 16) are shown to have no significant difference (p = 0.2259). (C) Significant difference between heterozygous females and males (p = 0.0007), and significant difference between homozygous females and males (p = 0.0067). (D) Raw crystal cell count of heterozygous females (n = 17) and homozygous females (n = 16) are shown to have no significant difference (p = 0.3339). (E) Raw crystal cell count of heterozygous males (n = 21) and homozygous males (n = 16) are shown to have no significant difference (p = 0.6017). (F) Significant difference between heterozygous females and males (p = 0.0463), and significant difference between homozygous females and males (p < 0.0001). (G) Raw domeMESO+ progenitor count of heterozygous females (n = 17) and homozygous females (n = 16) are shown to have no significant difference (p = 0.7122). (H) Raw domeMESO+ progenitor count of heterozygous males (n = 19) and homozygous males (n = 16) are shown to have no significant difference (p = 0.9136). (I) Significant difference between heterozygous females and males (p = 0.0022), and significant difference between homozygous females and males (p = 0.0025).
(PDF)

S1 Data All raw and corrected cell counts for male and female *w1118 x tep4 GFP* wildtype flies. (PDF)

S2 Data All raw cell counts for total nuclei, crystal cells, and domeMESO+ progenitors for Fig 3 (CNS feminization), including both UAS and gal4 controls, and feminized experimental group. (PDF)

S3 Data All raw cell counts for total nuclei, crystal cells, and domeMESO+ progenitors for Fig 4 (fat body feminization), including both UAS and gal4 controls, and feminized experimental group. (PDF)

S4 Data All raw cell counts for total nuclei, crystal cells, and domeMESO+ progenitors for Fig 5 (domeMESO+ progenitor feminization), including both UAS and gal4 controls, and feminized experimental group. (PDF)

S5 Data All raw cell counts for total nuclei, crystal cells, and domeMESO+ progenitors for Fig 6 (PSC feminization), including both UAS and gal4 controls, and feminized experimental group. (PDF)

S6 Data All raw cell counts for total nuclei, crystal cells, and domeMESO+ progenitors for Fig 7 (insulin receptor knockdown in the PSC), including both UAS and gal4 controls, and experimental group. (PDF)

S7 Data All raw cell counts for total nuclei, crystal cells, and domeMESO+ progenitors for Fig 8 (constitutively active insulin receptor in the PSC), including both UAS and gal4 controls, and experimental group. (PDF)

S8 Data All raw cell counts for total nuclei, crystal cells, domeMESO+ progenitors, and plasmatocytes for Fig 9, including both control and infected males and females with *E.coli.* (PDF)

S9 Data All raw cell counts for total nuclei, crystal cells, domeMESO+ progenitors, and plasmatocytes for Fig 10, including both control and infected males and females with *P.carotovorum.* (PDF)
