# Supplementary material for: Sex differences in the regulation and function of cellular immunity in Drosophila
Source: PLoS Genet. 2026 Jul 10;22(7):e1012151. doi: 10.1371/journal.pgen.1012151 (PMC13399539; doi:10.1371/journal.pgen.1012151)
Supplement: S1 File — We set a threshold of 1.5 fold change, and <=0.0001 for the FDR step-up, discarding any genes which did not meet both thresholds. (PDF) [file pgen.1012151.s002.pdf]

	A	B	C	D	E	F	G	H	I	J
1	**Female Genes overall**					**Male Genes Overalll**				
2	**Gene**	**FDR**	**Fold change**	**LSMean**		**Gene**	**FDR**	**Fold change**	**LSMean**	
3	lncRNA:CR40469	0.00E+00	25.66	381.49		lncRNA:roX1	0.00E+00	#####	589.04	
4	CG43133	0.00E+00	14.02	101.7		lncRNA:roX2	0.00E+00	#####	311.00	
5	CG32816	2.52E-181	4.43	10.245		msl-2	0.00E+00	3.90	16.18	
6	CR43214	0.00E+00	3.92	16.865		CG32706	#######	2.67	6.93	
7	Cht2	3.16E-135	2.63	13.861		CG6999	1.88E-93	2.63	4.40	
8	CG3038	1.13E-178	2.47	12.618		CG15739	#######	2.56	9.43	
9	CR43215	8.34E-77	2.45	2.8777		lncRNA:CR45979	8.87E-64	2.18	2.70	
10	CR43211	0.00E+00	2.20	55.7		D2hgdh	#######	2.13	11.52	
11	CG15784	1.59E-35	2.18	25.842		CG4586	1.44E-60	2.07	3.35	
12	MCTS1	0.00E+00	2.16	145.67		comt	2.25E-72	2.06	3.58	
13	CG4301	1.41E-76	2.11	10.018		CG3568	1.96E-96	2.04	10.51	
14	CG9733	6.28E-33	2.11	14.48		CG5273	#######	2.03	8.93	
15	Cpr5C	2.87E-05	2.08	2.5977		CG10859	8.78E-47	2.03	3.06	
16	Tsp3A	1.77E-114	2.01	10.893		jv	1.59E-25	1.98	6.33	
17	NimC1	2.36E-29	2.01	16.193		CG16957	2.88E-34	1.84	2.90	
18	CG14434	0.00E+00	1.93	81.315		lncRNA:CR32582	#######	1.82	18.83	
19	CG32641	5.87E-60	1.90	7.3755		fs(1)Yb	7.70E-51	1.81	1.94	
20	CecB	7.02E-143	1.87	386.9		CG17919	2.24E-12	1.79	3.62	
21	sano	4.07E-17	1.85	6.8053		corolla	5.62E-35	1.73	2.01	
22	Sxl	0.00E+00	1.79	67.863		CR18166	1.94E-43	1.72	3.26	
23	CR43212	4.15E-85	1.79	14.163		CG7997	#######	1.71	57.40	
24	AnxB11	2.73E-279	1.78	91.913		CG11655	3.93E-65	1.71	7.39	
25	Crg-1	1.98E-33	1.78	2.4806		lncRNA:CR45625	1.58E-49	1.68	5.81	
26	dpr14	8.87E-30	1.75	3.7694		CG3176	#######	1.68	20.13	
27	CecA1	1.42E-115	1.73	199.71		CG15317	4.99E-64	1.68	10.23	
28	CG34054	1.44E-41	1.72	22.984		AttA	5.52E-08	1.66	7.24	
29	Karl	0.00E+00	1.66	453.78		CG42369	1.84E-56	1.66	47.76	
30	wus	4.81E-28	1.64	3.3782		CG33181	6.36E-25	1.64	4.20	

	A	B	C	D	E	F	G	H	I	J
31	Arpc3B	0.00E+00	1.64	300.2		sug	4.54E-41	1.59	26.14	
32	unc-13	1.83E-64	1.61	17.824		CG4239	1.04E-54	1.58	10.84	
33	CG31821	2.05E-12	1.61	14.254		Su(Ste):CR42415	3.57E-21	1.58	1.58	
34	mthl4	4.58E-24	1.60	6.6094		wde	4.60E-91	1.56	35.11	
35	DIP-alpha	1.95E-31	1.59	6.8221		mof	1.79E-38	1.56	6.63	
36	CecC	2.19E-98	1.59	1022.3		CG43867	1.00E-21	1.55	2.71	
37	Tsp42Ed	4.64E-06	1.58	4.105		CG5167	1.87E-87	1.53	28.91	
38	prage	2.10E-131	1.57	193.77		MtnA	3.86E-26	1.53	390.56	
39	CG33225	7.05E-25	1.57	27.969		aay	2.45E-06	1.52	27.21	
40	CG3587	1.38E-39	1.56	8.418		CkIIalpha-i3	1.54E-43	1.51	10.51	
41	Rip11	4.06E-100	1.56	158.92		TM4SF	5.63E-10	1.50	3.58	
42	CG9981	5.42E-20	1.56	2.7021						
43	CG10804	9.43E-19	1.55	2.3367						
44	CecA2	4.25E-73	1.55	419.81						
45	CG4593	3.68E-177	1.55	71.598						
46	Sox15	1.10E-18	1.53	1.6546						
47	CG1840	8.05E-105	1.53	42.534						
48	lawc	5.04E-120	1.52	59.021						
49	png	1.68E-18	1.52	4.3765						
50	CG5004	1.12E-115	1.51	88.79						
51	CG3781	1.98E-109	1.51	36.792						
52	RhoGAP18B	2.49E-182	1.51	143.34						
53										
54										
55										
56										
57										
58										
59										
