# Supplementary material for: Sex differences in the regulation and function of cellular immunity in Drosophila
Source: PLoS Genet. 2026 Jul 10;22(7):e1012151. doi: 10.1371/journal.pgen.1012151 (PMC13399539; doi:10.1371/journal.pgen.1012151)
Supplement: S2 File — We set a threshold of 1.5 fold change, and <=0.0001 for the FDR step-up, discarding any genes which did not meet both thresholds. (PDF) [file pgen.1012151.s003.pdf]

	K	L	M	N	O	P	Q	R	S	T
1	**Female Genes MZ**					**Male Genes MZ**				
2	**Gene**	**FDR**	**Fold change**	**LSMean**		**Gene**	**FDR**	**Fold change**	**LSMean**	
3	lncRNA:CR40469	#######	36.88	340.59		lncRNA:roX1	#######	596.90	596.90	
4	CG43133	#######	20.83	185.74		lncRNA:roX2	#######	340.04	340.04	
5	CR43214	#######	3.95	17.40		msl-2	#######	4.26	15.99	
6	Cht2	8.06E-12	3.08	5.24		comt	3.67E-28	2.73	3.66	
7	CR43215	2.00E-26	2.65	3.08		CG15739	5.97E-22	2.41	6.07	
8	CR43211	#######	2.26	61.91		CG3176	2.89E-43	2.28	16.37	
9	CG3038	3.95E-35	2.24	10.07		CG32706	1.03E-17	2.27	5.57	
10	MCTS1	#######	2.19	148.24		CG32625	2.68E-13	2.21	4.55	
11	CG9733	2.75E-11	2.10	15.20		CG5273	2.93E-11	2.13	4.99	
12	CG14434	#######	2.05	99.87		CG11655	1.22E-16	2.13	5.60	
13	CecB	2.72E-46	1.97	419.45		CG6999	1.81E-10	2.08	3.00	
14	CG15784	9.24E-08	1.96	11.55		lncRNA:CR32582	1.35E-35	2.06	16.03	
15	CR43212	2.02E-39	1.94	16.33		lncRNA:CR45979	1.10E-08	1.87	2.33	
16	Sxl	#######	1.94	61.22		CG43867	1.86E-08	1.86	2.64	
17	CG31821	7.97E-13	1.91	14.98		CG16957	1.58E-08	1.82	3.07	
18	CG32641	7.41E-17	1.88	6.57		CR18166	3.01E-12	1.81	3.10	
19	lncRNA:CR43264	2.46E-06	1.87	5.79		CG4586	3.97E-07	1.79	2.34	
20	wus	1.27E-11	1.84	3.44		CG3568	9.47E-06	1.78	4.78	
21	CecA1	8.94E-64	1.83	295.28		CG15317	1.10E-20	1.77	11.11	
22	lncRNA:CR30009	4.44E-23	1.81	10.82		mof	1.27E-15	1.77	7.01	
23	AnxB11	1.39E-87	1.78	100.90		CkIIalpha-i3	2.86E-12	1.77	7.93	
24	His2B:CG17949	2.93E-09	1.76	2.68		nkd	6.66E-04	1.76	2.83	
25	Tsp3A	1.35E-19	1.74	8.76		CG1998	8.83E-06	1.76	3.08	
26	CecC	1.29E-45	1.72	#####		CG5167	2.97E-19	1.72	21.20	
27	CG3587	1.00E-15	1.71	8.08		D2hgdh	4.97E-07	1.70	5.31	
28	CG10804	2.33E-09	1.66	2.72		corolla	2.37E-08	1.68	2.16	
29	Fas3	2.10E-07	1.66	7.85		wde	2.24E-41	1.67	44.62	
30	Arpc3B	#######	1.65	240.51		CG7997	2.53E-36	1.65	38.94	

	K	L	M	N	O	P	Q	R	S	T
31	Atf3	5.41E-64	1.64	58.45		GstT3	3.35E-19	1.65	14.81	
32	Wsck	2.93E-05	1.64	3.05		CG43759	6.20E-05	1.63	2.18	
33	unc-13	2.35E-22	1.62	17.67		lncRNA:CR4562	1.61E-07	1.63	4.59	
34	CG3781	1.23E-36	1.61	32.67		Hsp70Aa	1.68E-09	1.59	42.11	
35	CG12056	1.79E-11	1.61	9.63		CG6762	6.02E-11	1.59	10.61	
36	Karl	#######	1.61	274.16		CG4239	3.13E-10	1.59	9.11	
37	Chrac-16	3.12E-30	1.60	19.36		ewg	5.52E-13	1.56	13.67	
38	CG31689	1.09E-05	1.60	2.51		sdk	2.74E-05	1.53	2.59	
39	CecA2	3.74E-54	1.60	815.30		CG32817	2.60E-06	1.50	2.44	
40	lncRNA:CR45517	1.81E-08	1.60	4.14						
41	DIP-alpha	3.06E-11	1.60	6.84						
42	CG5004	1.72E-51	1.58	98.01						
43	CG33225	1.73E-26	1.58	73.29						
44	Crg-1	8.60E-06	1.57	2.03						
45	CG4593	1.96E-58	1.57	72.43						
46	CR43217	4.89E-07	1.56	2.44						
47	CG17982	2.82E-35	1.56	38.32						
48	CG11590	3.08E-56	1.55	60.98						
49	CG7322	9.06E-25	1.55	25.78						
50	CG34331	9.85E-54	1.55	63.85						
51	hpRNA:CR32207	1.99E-14	1.55	17.62						
52	lncRNA:CR42868	1.05E-14	1.53	9.64						
53	RNaseP:RNA	1.70E-06	1.53	2.23						
54	Dab	2.24E-06	1.53	4.46						
55	Gclc	3.62E-34	1.52	72.53						
56	CG2444	6.36E-05	1.52	445.41						
57	rdgB	1.22E-20	1.51	15.58						
58	CG17109	1.58E-04	1.51	7.16						
59	Aven	4.82E-08	1.50	7.00						
