# Supplementary material for: Sex differences in the regulation and function of cellular immunity in Drosophila
Source: PLoS Genet. 2026 Jul 10;22(7):e1012151. doi: 10.1371/journal.pgen.1012151 (PMC13399539; doi:10.1371/journal.pgen.1012151)
Supplement: S3 File — We set a threshold of 1.5 fold change, and <=0.0001 for the FDR step-up, discarding any genes which did not meet both thresholds. (PDF) [file pgen.1012151.s004.pdf]

	U	V	W	X	Y	Z	AA	AB	AC	AD
1	**Female Genes CCs**					**Male Genes CCs**				
2	**Gene**	**FDR**	**Fold change**	**LSMean**		**Gene**	**FDR**	**Fold change**	**LSMean**	
3	lncRNA:CF	#######	25.78	276.85		lncRNA:roX1	#######	555.08	555.08	
4	CG43133	1.00E+00	5.33	17.22		lncRNA:roX2	#######	205.59	205.59	
5	CG32816	1.00E+00	3.65	7.15		jv	1.26E-05	7.45	18.19	
6	CG17778	5.18E-01	2.97	5.49		msl-2	7.19E-49	6.18	30.48	
7	Gasp	2.23E-03	2.72	3.01		CG15739	5.54E-86	5.11	88.87	
8	MCTS1	1.42E-12	2.22	136.44		lncRNA:CR459	1.62E-10	3.71	4.14	
9	CG14434	4.08E-02	2.16	64.63		CG6999	9.51E-04	3.30	3.62	
10	CG30148	5.83E-01	2.01	63.36		CG11318	7.55E-81	2.89	11.39	
11	Karl	5.55E-05	1.97	148.75		trol	4.87E-08	2.74	20.88	
12	CG10527	6.53E-01	1.86	45.36		lncRNA:CR325	5.66E-14	2.18	24.46	
13	alpha-Est4	1.00E+00	1.82	3.81		Ilp6	8.55E-15	2.12	126.50	
14	CG14629	7.31E-01	1.74	602.40		CG9119	6.40E-31	2.09	2819.06	
15	CG4593	1.69E-01	1.72	73.33		asRNA:CR449	2.58E-22	2.05	5.85	
16	CG14270	9.01E-01	1.71	32.62		E23	2.50E-06	2.01	4.28	
17	lncRNA:CF	1.00E+00	1.68	4.96		snRNA:U6:96A	7.97E-04	2.01	2.01	
18	CrebB	9.98E-01	1.66	38.88		D2hgdh	5.65E-13	1.82	25.84	
19	Sxl	3.37E-02	1.65	82.29		CG34232	2.09E-06	1.82	25.58	
20	CG11590	2.59E-01	1.58	51.98		Jheh3	1.17E-26	1.78	4.77	
21	Arpc3B	1.00E+00	1.57	212.06		mthl14	1.51E-09	1.76	2.23	
22	Galphai	1.00E+00	1.56	63.82		Hk	7.70E-08	1.71	4.39	
23	Tep1	1.00E+00	1.56	152.46		CG2017	2.34E-05	1.70	10.88	
24	CG7453	8.17E-01	1.54	54.24		lncRNA:CR430	4.05E-04	1.70	17.93	
25	CG3939	2.21E-11	1.51	72.77		Tsp42Er	4.71E-46	1.67	15.87	
26						CG5418	8.46E-52	1.66	193.89	
27						lncRNA:CR448	3.95E-31	1.66	2.21	
28						Gp150	3.95E-04	1.65	10.71	
29						Vav	7.42E-08	1.62	21.31	
30						asRNA:CR461	1.99E-04	1.62	2.75	

	U	V	W	X	Y	Z	AA	AB	AC	AD
31						CG7997	1.16E-04	1.60	38.02	
32						AsnS	5.03E-07	1.59	41.05	
33						CG34120	3.08E-12	1.59	2.34	
34						PCNA2	1.47E-05	1.57	2.71	
35						CG11576	7.67E-19	1.57	24.41	
36						lncRNA:CR443	8.54E-11	1.56	1.92	
37						CG42240	4.56E-06	1.55	7.76	
38						mwh	1.79E-33	1.53	4.94	
39						lncRNA:CR447	1.44E-10	1.53	2.24	
40						CG6805	1.17E-07	1.52	66.02	
41						CG9008	7.10E-04	1.51	4.50	
42										
43										
44										
45										
46										
47										
48										
49										
50										
51										
52										
53										
54										
55										
56										
57										
58										
59										
