# Supplementary material for: Sex differences in the regulation and function of cellular immunity in Drosophila
Source: PLoS Genet. 2026 Jul 10;22(7):e1012151. doi: 10.1371/journal.pgen.1012151 (PMC13399539; doi:10.1371/journal.pgen.1012151)
Supplement: S4 File — We set a threshold of 1.5 fold change, and <=0.0001 for the FDR step-up, discarding any genes which did not meet both thresholds. (PDF) [file pgen.1012151.s005.pdf]

	AE	AF	AG	AH	AI	AJ	AK	AL
1	**Female Genes PSC**					**Male Genes PSC**		
2	**Gene**	**FDR**	**Fold change**	**LSMean**		**Gene**	**FDR**	**Fold change**
3	lncRNA:CR40469	########	160.00	1036.74		lncRNA:roX1	1.84E-295	757.50
4	CG6287	9.72E-05	1.78	797.79		lncRNA:roX2	1.38E-303	729.46
5	mthl7	1.93E-09	1.55	259.86		Lcp65Ab1	3.42E-07	328.85
6	CG15784	6.51E-12	2.22	234.56		Cpr47Ec	2.58E-06	227.69
7	Karl	5.55E-05	1.52	212.68		CG34227	8.84E-06	166.00
8	NimB5	5.21E-08	1.54	183.97		Lcp65Ab2	1.26E-06	153.09
9	CG3939	2.21E-11	2.04	177.26		CG13403	1.22E-09	114.35
10	Cpr5C	0.00E+00	6.24	150.59		dpy	3.89E-11	67.00
11	ImpL2	6.87E-07	1.82	141.46		Ets98B	1.20E-14	45.48
12	CG10508	3.26E-09	1.53	130.26		fj	1.18E-16	41.17
13	Vps2	1.06E-04	1.75	126.61		SKIP	4.65E-17	29.50
14	MCTS1	1.42E-12	2.71	119.63		slf	2.93E-07	26.83
15	CR43211	4.07E-04	2.20	91.09		CG9449	2.72E-05	23.62
16	Hers	6.95E-04	1.83	90.96		CG34398	8.91E-05	15.35
17	CG13369	2.14E-05	2.02	90.33		pk	4.59E-13	10.98
18	CG12112	2.10E-04	1.77	89.77		ovo	2.14E-05	7.61
19	TkR99D	########	2.32	83.48		lncRNA:CR43432	4.87E-05	5.42
20	CG43759	2.23E-15	1.67	66.29		mirr	4.62E-06	4.48
21	CG9220	2.19E-24	1.66	39.03		CG8526	2.31E-05	3.87
22	CG14855	1.78E-24	1.90	38.67		CG46339	1.48E-11	3.33
23	hlk	9.73E-09	1.58	37.03		CG34370	3.05E-04	3.26
24	snRNA:U2:14B	7.98E-06	2.33	34.09		NetA	1.42E-43	3.05
25	CG15365	3.08E-16	1.56	29.77		Picot	1.90E-04	3.00
26	comm	1.50E-06	1.64	20.75		axed	9.35E-07	2.95
27	lncRNA:CR44933	2.72E-22	1.53	20.26		NetB	1.23E-09	2.90
28	CG8369	7.17E-09	1.72	15.85		Ilp6	6.10E-07	2.77
29	Ace	1.53E-10	1.75	14.94		CG8547	5.65E-19	2.69
30	CCKLR-17D3	2.21E-11	1.61	14.16		Hsp70Bb	6.77E-05	2.67

	AE	AF	AG	AH	AI	AJ	AK	AL
31	Idgf4	1.01E-23	3.51	13.60		Ca-alpha1D	4.88E-07	2.67
32	Dl	8.10E-08	4.20	12.82		Npc2a	5.96E-09	2.60
33	asRNA:CR45912	2.40E-45	4.58	11.42		CG1113	2.30E-04	2.58
34	comm2	1.61E-06	1.77	8.73		rdgA	9.57E-35	2.48
35	CG31183	2.14E-05	1.92	6.68		CG32280	3.15E-05	2.40
36	lncRNA:CR33218	2.62E-04	5.03	5.03		lncRNA:CR45959	5.38E-06	2.39
37	CG34002	9.26E-07	2.42	4.97		bark	2.03E-09	2.30
38	CG14516	1.96E-12	3.00	4.94		CG13284	8.48E-64	2.17
39	form3	5.28E-04	2.55	4.69		CG9095	2.83E-07	2.16
40	Septin4	9.72E-05	4.01	4.01		CG2781	2.99E-33	2.08
41	CG17321	7.43E-07	3.27	3.27		ko	1.04E-23	1.96
42	CG17121	5.33E-07	3.12	3.12		GILT2	1.62E-31	1.96
43	Dh31-R	3.41E-04	3.05	3.05		CG11134	9.59E-06	1.66
44						Ac13E	3.00E-04	1.66
45						Pgant2	2.11E-26	1.54
46								
47								
48								
49								
50								
51								
52								
53								
54								
55								
56								
57								
58								
59								

	AM	AN	AO	AP	AQ	AR	AS	AT	AU
1									
2	**LSMean**								
3	757.50								
4	729.46								
5	328.85								
6	227.69								
7	166.00								
8	153.09								
9	114.35								
10	67.00								
11	45.48								
12	41.17								
13	29.50								
14	26.83								
15	23.62								
16	15.35								
17	10.98								
18	7.61								
19	5.42								
20	4.48								
21	5.58								
22	7.82								
23	5.23								
24	7.14								
25	7.67								
26	10.87								
27	8.78								
28	144.20								
29	13.68								
30	10.15								

	AM	AN	AO	AP	AQ	AR	AS	AT	AU
31	2.67								
32	26.33								
33	2.58								
34	120.55								
35	18.76								
36	2.39								
37	4.21								
38	12.60								
39	3.74								
40	71.33								
41	28.85								
42	158.19								
43	77.29								
44	15.55								
45	22.90								
46									
47									
48									
49									
50									
51									
52									
53									
54									
55									
56									
57									
58									
59									
