# Supplementary material for: Sex differences in the regulation and function of cellular immunity in Drosophila
Source: PLoS Genet. 2026 Jul 10;22(7):e1012151. doi: 10.1371/journal.pgen.1012151 (PMC13399539; doi:10.1371/journal.pgen.1012151)
Supplement: S5 File — (PDF) [file pgen.1012151.s006.pdf]

Female Genes overall					Male Genes Overalll				
GO or pathway	ID	Adjusted p-value	Genes		GO or pathway	ID	Adjusted p-value	Genes	
flippase activity	GO:0140327	0.003349	CG4301,CG9981		sex chromosome	GO:0000803	0.01370	MSL-2,MOF	
ATPase-coupled intramembrane lipid transporter	GO:0140326	0.049811	CG4301,CG9981		X chromosome	GO:0000805	0.01370	MSL-2,MOF	
defense response to insect	GO:0002213	0.001438	CECB,CECA1,CECA2		X chromosome located dosage compensation complex, transcription activating	GO:0016456	0.01370	MSL-2,MOF	
defense response to Gram-positive	GO:0050830	0.028312	CECB,CECA1,CECC, CECA2		dosage compensation complex	GO:0046536	0.01370	MSL-2,MOF	
phospholipid-translocating ATPase complex	GO:1990531	0.005406	CG4301,CG9981		MSL complex	GO:0072487	0.02550	MSL-2,MOF	
Toll and Imd signaling pathway	KEGG:04624	0.001696	CECB,CECA1,CECC, CECA2						
Intrahepatic cholestasis with episodic jaundice	HP:0006575	0.008293	CG4301,CG9981						
Dysphoria	HP:0033838	0.008293	CG4301,CG9981						
Abnormal liver function tests during pregnancy	HP:0200148	0.008293	CG4301,CG9981						
Increased serum bile acid concentration during pregnancy	HP:0200150	0.008293	CG4301,CG9981						
Asterixis	HP:0012164	0.016572	CG4301,CG9981						

Pruritus on foot	HP:0030900	0.016572	CG4301,CG9981						
Abnormal speech discrimination	HP:0001963	0.027598	CG4301,CG9981						
