# Supplementary material for: Sex differences in the regulation and function of cellular immunity in Drosophila
Source: PLoS Genet. 2026 Jul 10;22(7):e1012151. doi: 10.1371/journal.pgen.1012151 (PMC13399539; doi:10.1371/journal.pgen.1012151)
Supplement: S9 File — These genes were selected for being enriched in one cell type, being broadly conserved across species (including in humans in most cases), and having some connection to blood cell development or disease, or immune cell phenotypes. References are included for each gene detailing the known roles of these genes or their mammalian orthologs in blood cell development, disease, or phenotypes. (PDF) [file pgen.1012151.s010.pdf]

| Gene | Enriched in | Summary | Reference |
|---|---|---|---|
| MCTS1 | female overall | Malignant T cell amplified sequence 1 is a translation reinitiation and ribosome recycling factor; mammalian ortholog is associated with Lymphoma and X-linked immunodeficiency | Prosniak et al. 1998; Bohlen et al. 2023 |
| Ilp6 | male multiple zones (PSC and CC) | Insulin-like peptide involved in crystal cell development in response to CO2/O2 gaseous imblance; mammalian ortholog involved in thrombopoiesis | Cho et al. 2018; Chen et al. 2018 |
| TkR99D | female PSC | Tachykinin-like peptide receptor, a class of GPCRs that respond to neuropeptides; mammalian orthologs involved in hematopoiesis | Liu et al. 2007 |
| rdgA | male PSC | Diacylglycerol kinase involved in phospholipase C signaling; loss of the mammalian ortholog DGKZ results hyperactive T cell response | Zhong et al. 2002 |
| CecB | female MZ | Anti-microbial peptide involved in the immune response | Carboni et al. 2022 |
| CG5167 | male MZ | putative ortholog SCCPDH (saccharopine dehydrogenase) involved in glycolipid biosynthesis; expressed on alveolar macrophages | Patel et al. 2017 |
| Tep1 | female CC | Complement-like protein involved in the innate immune response; human ortholog CD109 is seen on activated platelets, T cells, and a subset of HSPCs | Dostalova et al. 2017; Batal et al. 2025 |
| CG15739 | male CC | phosphatase involved in vitamin B metabolism and juvenile hormone synthesis; human ortholog PDXP is expressed in RBCs | Gohla 2019 |

References


1. Prosniak M, Dierov J, Okami K, Tilton B, Jameson B, Sawaya BE, et al. A novel candidate oncogene, MCT-1, is involved in cell cycle progression. Cancer Res. 1998;58(19):4233-4237.
2. Bohlen J, Zhou Q, Philippot Q, Ogishi M, Rinchai D, Nieminen T, et al. Human MCTS1-dependent translation of JAK2 is essential for IFN-γ immunity to mycobacteria. Cell. 2023;186(23):5114-5134.e27. doi:10.1016/j.cell.2023.09.024. PMID: 37875108.
3. Chen S, Hu M, Shen M, Wang S, Wang C, Chen F, et al. IGF-1 facilitates thrombopoiesis primarily through Akt activation. Blood. 2018;132(2):210-222. doi:10.1182/blood-2018-01-825927.
4. Liu K, Castillo MD, Murthy RG, Patel N, Rameshwar P. Tachykinins and hematopoiesis. Clin Chim Acta. 2007;385(1-2):28-34. doi:10.1016/j.cca.2007.07.008.
5. Zhong XP, Hainey EA, Olenchock BA, Zhao H, Topham MK, Koretzky GA. Regulation of T cell receptor-induced activation of the Ras-ERK pathway by diacylglycerol kinase zeta. J Biol Chem. 2002;277(34):31089-31098. doi:10.1074/jbc.M203818200.
6. Carboni S, Lepidi S, Ponzini E, Ghezzani C, Natale M, Paus R, et al. Antimicrobial peptide expression in the skin: relevance for innate immune responses and inflammatory diseases. Int J Mol Sci. 2022;23(5):2499. doi:10.3390/ijms23052499.
7. Patel VI, Booth JL, Duggan ES, Cate S, White VL, Hutchings D, et al. Transcriptional classification and functional characterization of human airway macrophage and dendritic cell subsets. J Immunol. 2017;198(3):1183-1201. doi:10.4049/jimmunol.1600777.
8. Dostálová A, Rommelaere S, Poidevin M, Lemaitre B. Thioester-containing proteins regulate the Toll pathway and play a role in Drosophila defence against microbial pathogens and parasitoid wasps. BMC Biol. 2017;15:79. doi:10.1186/s12915-017-0408-0.
9. Batal A, Garousi S, Finnson KW, Philip A. CD109, a master regulator of inflammatory responses. Front Immunol. 2025;15:1505008. doi:10.3389/fimmu.2024.1505008.
10. Gohla A. Do metabolic HAD phosphatases moonlight as protein phosphatases? Biochim Biophys Acta Mol Cell Res. 2019;1866(1):153-166. doi:10.1016/j.bbamcr.2018.08.016.
