# Supplementary material for: Sex differences in the regulation and function of cellular immunity in Drosophila
Source: PLoS Genet. 2026 Jul 10;22(7):e1012151. doi: 10.1371/journal.pgen.1012151 (PMC13399539; doi:10.1371/journal.pgen.1012151)
Supplement: S1 Table — Total number of sex determination genes separated by males and females, as well as total counts per cell. (PDF) [file pgen.1012151.s012.pdf]

Supplementary table 1. Total counts for sex determination genes

Gene	Total counts	
	Female (10242 cells)	Male (8865 cells)
lncRNA:roX1	10,242.00	5,221,802.35
lncRNA:roX2	10,242.00	2,756,999.65
msl-2	42,482.85	143,428.82
Sxl	695,048.41	336,273.68
tra	834,186.40	572,064.22

Gene	Total counts per cell	
	Female	Male
lncRNA:roX1	1.00	589.04
lncRNA:roX2	1.00	311.00
msl-2	4.15	16.18
Sxl	67.86	37.93
tra	81.45	64.53
