# Supplementary figures and images for: Sex differences in the regulation and function of cellular immunity in Drosophila
Source: PLoS Genet. 2026 Jul 10;22(7):e1012151. doi: 10.1371/journal.pgen.1012151 (PMC13399539; doi:10.1371/journal.pgen.1012151)

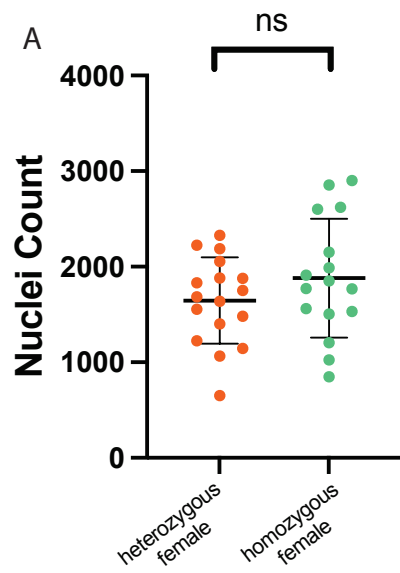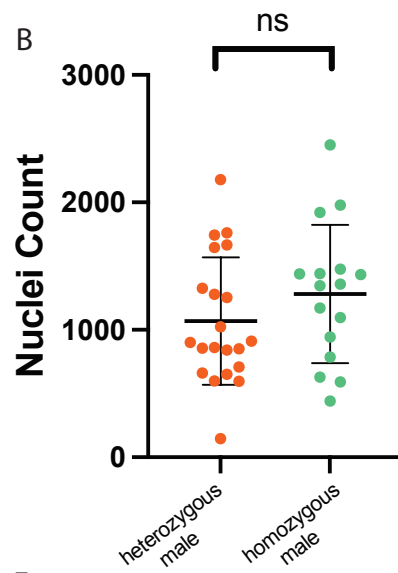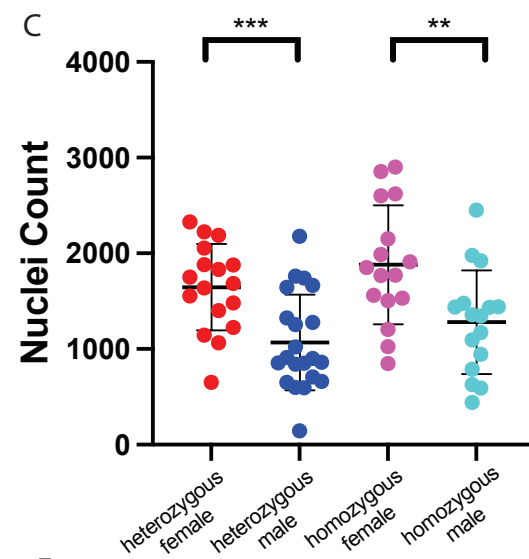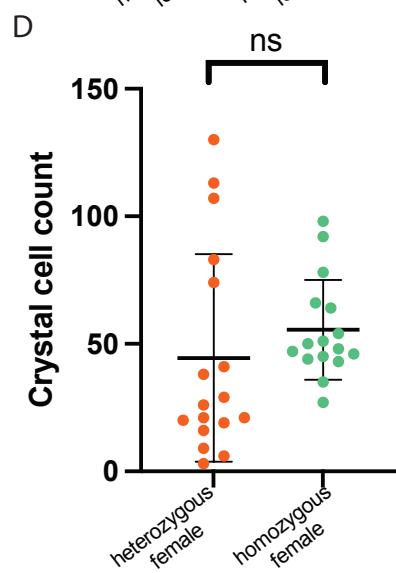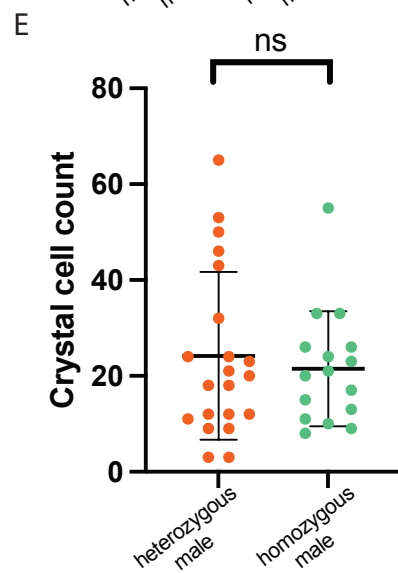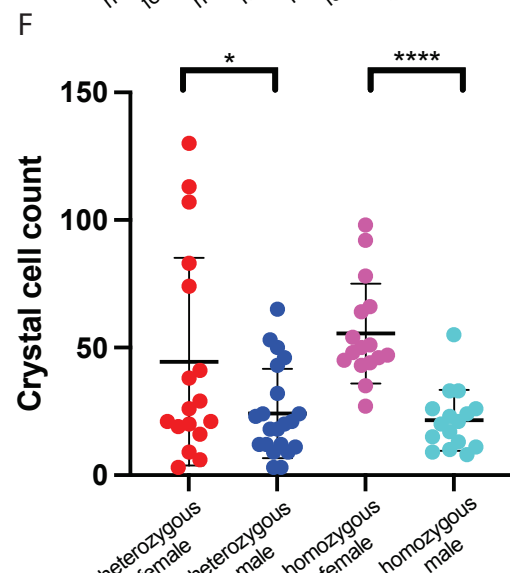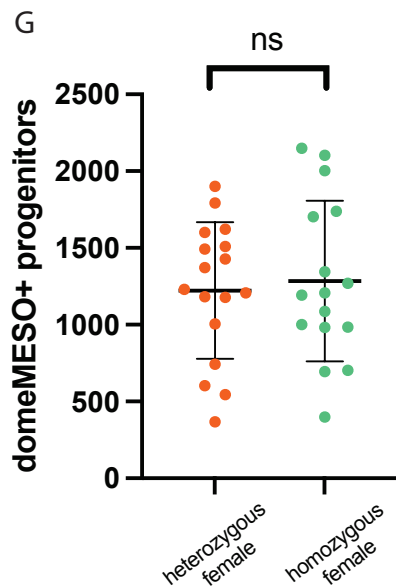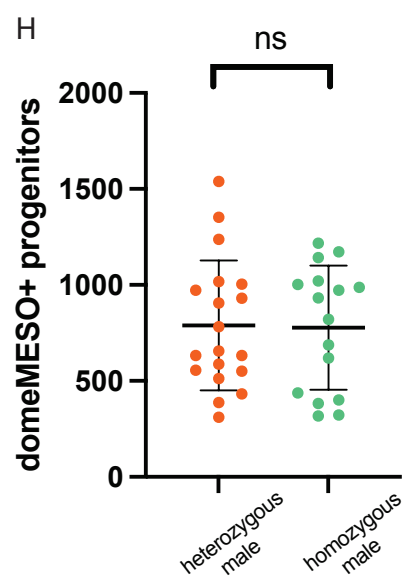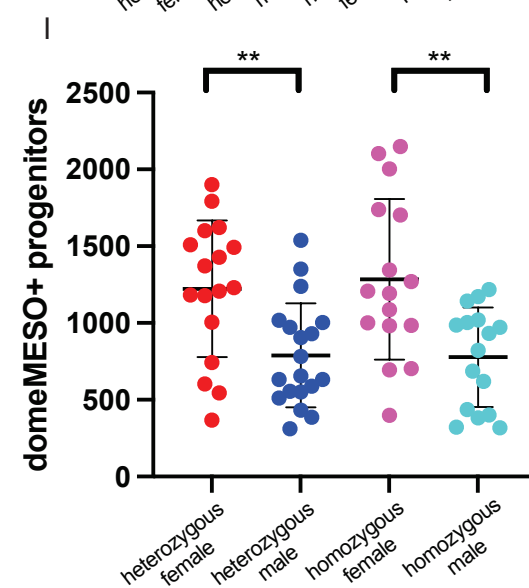

Supplement: S7 Fig — Heterozygous cross of UAS TraF; + x domeMESOGFP and homozygous cross of UAS TraF;domeMESOGFP x domeMESOGFP, separated by sex. (A) Raw total nuclei count of heterozygous females(n = 17) and homozygous females(n = 16) are shown to have no significant difference (p = 0.2208). (B) Raw total nuclei count of heterozygous males(n = 21) and homozygous males (n = 16) are shown to have no significant difference (p = 0.2259). (C) Significant difference between heterozygous females and males (p = 0.0007), and significant difference between homozygous females and males (p = 0.0067). (D) Raw crystal cell count of heterozygous females (n = 17) and homozygous females (n = 16) are shown to have no significant difference (p = 0.3339). (E) Raw crystal cell count of heterozygous males (n = 21) and homozygous males (n = 16) are shown to have no significant difference (p = 0.6017). (F) Significant difference between heterozygous females and males (p = 0.0463), and significant difference between homozygous females and males (p < 0.0001). (G) Raw domeMESO+ progenitor count of heterozygous females (n = 17) and homozygous females (n = 16) are shown to have no significant difference (p = 0.7122). (H) Raw domeMESO+ progenitor count of heterozygous males (n = 19) and homozygous males (n = 16) are shown to have no significant difference (p = 0.9136). (I) Significant difference between heterozygous females and males (p = 0.0022), and significant difference between homozygous females and males (p = 0.0025). (PDF) [file pgen.1012151.s019.pdf]
