# Supplementary material for: Sex differences in the regulation and function of cellular immunity in Drosophila
Source: PLoS Genet. 2026 Jul 10;22(7):e1012151. doi: 10.1371/journal.pgen.1012151 (PMC13399539; doi:10.1371/journal.pgen.1012151)
Supplement: S1 Data — (PDF) [file pgen.1012151.s020.pdf]

NUCLEI COUNT		PSC count		PROGENITOR Count		CRYSTAL CELL count		PLASMATOCYTE count		NUCLEI CORRECTED		PSC CORRECTED		PROGENITOR CORRECTED		CRYSTAL CELL CORRECTED		PLASMATOCYTES CORRECTED	
Female	Male	Female	Male	Female	Male	Female	Male	Female	Male	Female	Male	Female	Male	Female	Male	Female	Male	Female	Male
2207	1609	99	107	593	392	17	15	457	162	985.27	935.47	44.196	62.209	264.73	227.91	7.5893	8.7209	204.02	94.186
2039	1704	122	80	1026	551	24	7	388	766	910.27	990.7	54.464	46.512	458.04	320.35	10.714	4.0698	173.21	445.35
2000	1023	82	101	963	358	42	27	346	318	892.86	594.77	36.607	58.721	429.91	208.14	18.75	15.698	154.46	184.88
1792	1186	112	78	955	657	48	29	460	258	800	689.53	50	45.349	426.34	381.98	21.429	16.86	205.36	150
2412	1240	83	90	790	598	52	70	613	324	1076.8	720.93	37.054	52.326	352.68	347.67	23.214	40.698	273.66	188.37
2424	1220	117	87	772	350	90	30	654	202	1082.1	709.3	52.232	50.581	344.64	203.49	40.179	17.442	291.96	117.44
1829	1688	73	72	526	651	44	6	874	311	816.52	981.4	32.589	41.86	234.82	378.49	19.643	3.4884	390.18	180.81
1782	1720	117	90	585	654	29	9	507	207	795.54	1000	52.232	52.326	261.16	380.23	12.946	5.2326	226.34	120.35
1947	761	107	45	594	475	41	8	365	343	869.2	442.44	47.768	26.163	265.18	276.16	18.304	4.6512	162.95	199.42
1850	769	93	55	657	314	58	17	366	249	825.89	447.09	41.518	31.977	293.3	182.56	25.893	9.8837	163.39	144.77
1673	2083	95	63	1106	759	67	32	764	424	746.88	1211	42.411	36.628	493.75	441.28	29.911	18.605	341.07	246.51
2018	2181	88	100	898	856	59	14	685	292	900.89	1268	39.286	58.14	400.89	497.67	26.339	8.1395	305.8	169.77
1943	1283	78	48	795	685	30	20	212	386	867.41	745.93	34.821	27.907	354.91	398.26	13.393	11.628	94.643	224.42
2034	1174	89	58	892	587	29	22	539	409	908.04	682.56	39.732	33.721	398.21	341.28	12.946	12.791	240.63	237.79
1887	1536	48	55	941	742	59	11	324	385	842.41	893.02	21.429	31.977	420.09	431.4	26.339	6.3953	144.64	223.84
2222	1532	73	47	620	963	57	8	330	448	991.96	890.7	32.589	27.326	276.79	559.88	25.446	4.6512	147.32	260.47
1970	1499	96	65	652	560	22	30	425	325	879.46	871.51	42.857	37.791	291.07	325.58	9.8214	17.442	189.73	188.95
1440	1597	124	77	425	556	48	58	319	215	642.86	928.49	55.357	44.767	189.73	323.26	21.429	33.721	142.41	125
2393	1583	75	59	1016	707	46	13	243	337	1068.3	920.35	33.482	34.302	453.57	411.05	20.536	7.5581	108.48	195.93
2323	1514	102	68	962	815	28	10	225	186	1037.1	880.23	45.536	39.535	429.46	473.84	12.5	5.814	100.45	108.14
2183	1149	99	56	839	667	42		367	286	974.55	668.02	44.196	32.558	374.55	387.79	18.75		163.84	166.28
1270	1254	136	38	860	599	44		178	533	566.96	729.07	60.714	22.093	383.93	348.26	19.643		79.464	309.88
2618	1725	83	64	1091	433	58		402		1168.8	1002.9	37.054	37.209	487.05	251.74	25.893		179.46	
2011	1786	82	94	900	727	59		323		897.77	1038.4	36.607	54.651	401.79	422.67	26.339		144.2	
2524	1931	157	85	1011	440	18		362		1126.8	1122.7	70.089	49.419	451.34	255.81	8.0357		161.61	
2542	1689	93	77	1332	476	72		264		1134.8	981.98	41.518	44.767	594.64	276.74	32.143		117.86	
1781	1750	67	90	727	922			313		795.09	1017.4	29.911	52.326	324.55	536.05			139.73	
1936	1664	92	100	791	562			377		864.29	967.44	41.071	58.14	353.13	326.74			168.3	
2828	1083	100	36	1030	328			185		1262.5	629.65	44.643	20.93	459.82	190.7			82.589	
2730	1030	51	7	949	420			88		1218.8	598.84	22.768	4.0698	423.66	244.19			39.286	
2772	1061	74	78	835	431					1237.5	616.86	33.036	45.349	372.77	250.58				
2245	1060	102	65	839	440					1002.2	616.28	45.536	37.791	374.55	255.81				
2552	1170	119	56	558	488					1139.3	680.23	53.125	32.558	249.11	283.72				
2387	1539	108	52	721	525					1065.6	894.77	48.214	30.233	321.88	305.23				
1940	1400	75	86	1169	429					866.07	813.95	33.482	50	521.88	249.42				
2365	1906	99	59	1232	500					1055.8	1108.1	44.196	34.302	550	290.7				
2587	1869	132	46	819	813					1154.9	1086.6	58.929	26.744	365.63	472.67				
1676	1964	78	101	850	566					748.21	1141.9	34.821	58.721	379.46	329.07				
1405	1660	86	75	355	455					627.23	965.12	38.393	43.605	158.48	264.53				
1935	1784	74	52	442	716					863.84	1037.2	33.036	30.233	197.32	416.28				
2204	1609	138	70	567	608					983.93	935.47	61.607	40.698	253.13	353.49				
2010	1155	91	58	1019	579					897.32	671.51	40.625	33.721	454.91	336.63				
1775	1162	80	85	840	579					792.41	675.58	35.714	49.419	375	336.63				
1838	1371	99	87	322	686					820.54	797.09	44.196	50.581	143.75	398.84				
2319	2343	55	45	679	910					1035.3	1362.2	24.554	26.163	303.13	529.07				
1641	1437	85	90	475	486					732.59	835.47	37.946	52.326	212.05	282.56				
1796	1692	53	50	900	997					801.79	983.72	23.661	29.07	401.79	579.65				
1445	1747	34	71	412	771					645.09	1015.7	15.179	41.279	183.93	448.26				
1205	1247	57	54	585	567					537.95	725	25.446	31.395	261.16	329.65				
985	1899	79	78	346	674					439.73	1104.1	35.268	45.349	154.46	391.86				
2156	1844	112	7	609	878					962.5	1072.1	50	4.0698	271.88	510.47				
2317	2423	81	72	446	691					1034.4	1408.7	36.161	41.86	199.11	401.74				
2353	2549	89	105	556	609					1050.4	1482	39.732	61.047	248.21	354.07				
2732	1475	66	29	745	857					1219.6	857.56	29.464	16.86	332.59	498.26				
2524	1737	92	55	1003	587					1126.8	1009.9	41.071	31.977	447.77	341.28				
2751	2076	60	68	1570	830					1228.1	1207	26.786	39.535	700.89	482.56				
2726	2316	80	85	1293	1024					1217	1346.5	35.714	49.419	577.23	595.35				
2549	2129	51	81	979	810					1137.9	1237.8	22.768	47.093	437.05	470.93				
2269	2042	148	85	664	1191					1012.9	1187.2	66.071	49.419	296.43	692.44				
3109		113		1228						1387.9		50.446		548.21					
3133		59		1607						1398.7		26.339		717.41					
3568		109		955						1592.9		48.661		426.34					
3297		99		1248						1471.9		44.196		557.14					
2559		79		925						1142.4		35.268		412.95					
