# Supplementary material for: Sex differences in the regulation and function of cellular immunity in Drosophila
Source: PLoS Genet. 2026 Jul 10;22(7):e1012151. doi: 10.1371/journal.pgen.1012151 (PMC13399539; doi:10.1371/journal.pgen.1012151)
Supplement: S2 Data — (PDF) [file pgen.1012151.s021.pdf]

NUCLEI								CRYSTAL CELL								PROGENITORS						
FEMALE				MALE				FEMALE				MALE				FEMALE				MALE		
elav gal4	UAS/TraF	TraF		elav gal4	UAS/TraF	TraF		elav gal4	UAS/TraF	TraF		elav gal4	UAS/TraF	TraF		elav gal4	UAS/TraF	TraF		elav gal4	UAS/TraF	TraF
2697	3175	1562		2091	1757	1520		14	73	15		13	25	15		494	1656	1207		1141	824	714
4249	3031	1908		2392	1576	955		43	91	24		12	15	1		1996	1467	1471		943	413	477
3515	1891	2794		1495	1339	2388		40	85	23		45	30	3		1617	744	1686		580	780	1409
3359	3066	2306		1443	1533	2049		46	89	14		19	23	16		1560	790	1501		535	768	1265
2224	3255	1906		2222	1239	1752		37	59	16		27	17	10		821	1650	1049		1046	483	965
2603	3546	2615		2207	1089	1204		77	50	29		37	10	13		1228	2237	1326		1177	498	725
2235	4251	2692		2716	3061	1665		87	110	48		17	48	3		868	2005	2003		1502	1161	1287
2228	3229	2462		2100	2831	2393		51	45	41		18	64	7		1137	1667	1396		944	1546	1205
2364	2747	3518		1554	2884	2192		47	79	21		52	37	57		987	945	1845		742	1280	1261
2212	4116	3243		1174	2310	2065		29	107	72		37	43	24		928	1631	2127		733	1143	1193
1649	2454	2867		1746	2986	2225		15	76	98		51	74	30		971	918	1726		905	1179	1316
2143	2468	2882		1872	2646	1108		81	64	51		33	79	26		761	1252	1705		914	1083	719
2159	2533	3365		1895	2212	1896		54	40	77		38	82	20		983	1034	1640		941	854	1032
1985	2824	1795		1660	1849	1208		53	31	54		27	54	21		980	960	1278		704	752	805
2750	2108	3093		2622	1407	1962		28	55	48		69	32	33		1433	845	1898		1140	803	994
2749	2216	3238		2188	2040	2096		26	20	38		40	20	23		1495	969	2014		1112	1351	1116
3060	2665	4139		1874	2688	1405		42	54	60		22	96	19		1143	1128	1977		1132	1361	1055
3610	3509	2223		1443	2199	1587		65	52	71		7	71	7		1716	1433	1615		1078	1099	951
4046	6297	2190				1762		116	99					6		1803	1562	1317				1245
	2117	1737				1766			24					3			1194	1350				1207
	3462	2594				1374			50					51			1209	1583				885
		3501												36				1876				
		3140												55				1720				
		3098												37				1447				
		2192																1713				
		2391																1512				
		2370																1605				
