# Supplementary material for: Sex differences in the regulation and function of cellular immunity in Drosophila
Source: PLoS Genet. 2026 Jul 10;22(7):e1012151. doi: 10.1371/journal.pgen.1012151 (PMC13399539; doi:10.1371/journal.pgen.1012151)
Supplement: S3 Data — (PDF) [file pgen.1012151.s022.pdf]

NUCLEI								CRYSTAL CELL								PROGENITORS						
FEMALE				MALE				FEMALE				MALE				FEMALE				MALE		
R4 gal4	UAS/TraF	TraF		R4 gal4	UAS/TraF	TraF		R4 gal4	UAS/TraF	TraF		R4 gal4	UAS/TraF	TraF		R4 gal4	UAS/TraF	TraF		R4 gal4	UAS/TraF	TraF
1183	2553	1562		924	1731	1520		24	59	15		5	67	15		536	820	1207		686	541	714
1401	3368	1908		1098	2958	955		16	60	24		15	71	1		870	953	1471		658	893	477
1131	3958	2794		856	2405	2388		16	94	23		22	86	3		760	1277	1686		351	708	1409
918	3674	2306		775	2433	2049		22	86	14		8	56	16		500	1049	1501		474	809	1265
1766	2263	1906		855	2390	1752		31	64	16		2	95	10		1090	1229	1049		439	826	965
1767	2470	2615		1037	1157	1204		87	87	29		23	43	13		636	653	1326		716	482	725
2113	2255	2692		1602	2776	1665		65	87	48		20	61	3		986	296	2003		858	815	1287
1745	1916	2462		1009	1935	2393		50	60	41		27	41	7		831	847	1396		607	538	1205
2284	2302	3518		927	1660	2192		50	78	21		35	15	57		1314	1054	1845		753	668	1261
2611	1966	3243		1457	1501	2065		90	72	72		20	18	24		891	1022	2127		717	761	1193
2305	3651	2867		1057	1422	2225		57	93	98		17	26	30		1310	1289	1726		602	643	1316
2194	3992	2882		1533	1378	1108		46	116	51		26	20	26		1055	1277	1705		893	767	719
2233	3364	3365		2163	1502	1896		57	74	77		48	23	20		754	1525	1640		1126	714	1032
1618	3789	1795		892	2747	1208		40	71	54		41	112	21		671	1360	1278		468	712	805
2807	2512	3093		905	2571	1962		38	103	48		83	73	33		1508	1216	1898		503	1086	994
1993	3007	3238		1198	1733	2096		42	71	38		10	21	23		1090	1667	2014		719	586	1116
2192	1897	4139		1124	1812	1405		108	53	60		8	40	19		854	666	1977		725	836	1055
2806	1645	2223		1484	1520	1587		96	41	71		1	23	7		1033	624	1615		653	841	951
1155	2356	2190		1152	1205	1762		14	71			2	9	6		630	768	1317		651	752	1245
1046	2547	1737		865	2390	1766		20	62			20	78	3		623	1218	1350		616	752	1207
1755	2277	2594		807	2822	1374		72	26			18	86	51		906	1246	1583		573	750	885
2163	3097	3501			1731			66	57			39	40	36		896	1291	1876			644	
	3409	3140			1800				94			29	40	55			1324	1720			754	
	3707	3098			2509				101			13	100	37			1576	1447			771	
	3571	2192			2628				79			6	106				1721	1713			924	
	3202	2391			1933				37				18				1760	1512			871	
	3715	2370			1961				130				26				713	1605			1063	
	2894				1693				43				52				1209				745	
	2088				2749				93				70				706				1191	
	1746				1618				50				14				727				767	
	2235				1969				61				17				765				1195	
	2407								58								613					
