# Supplementary material for: Sex differences in the regulation and function of cellular immunity in Drosophila
Source: PLoS Genet. 2026 Jul 10;22(7):e1012151. doi: 10.1371/journal.pgen.1012151 (PMC13399539; doi:10.1371/journal.pgen.1012151)
Supplement: S4 Data — (PDF) [file pgen.1012151.s023.pdf]

NUCLEI								CRYSTAL CELL								PROGENITORS						
FEMALE				MALE				FEMALE				MALE				FEMALE				MALE		
tep4gal4	UAS/TraF	TraF		tep4gal4	UAS/TraF	TraF		tep4gal4	UAS/TraF	TraF		tep4gal4	UAS/TraF	TraF		tep4gal4	UAS/TraF	TraF		tep4gal4	UAS/TraF	TraF
2804	3110	1562		870	1782	1520		46	14	15		4	17	15		1639	1715	1207		625	992	714
2033	2829	1908		1520	2400	955		25	16	24		2	21	1		1186	1209	1471		694	574	477
1760	1959	2794		1574	2177	2388		40	33	23		66	42	3		1009	756	1686		525	751	1409
2015	2093	2306		1694	2382	2049		18	34	14		72	33	16		1227	1057	1501		699	769	1265
2918	2898	1906		2006	3231	1752		56	27	16		27	32	10		1467	1559	1049		1238	1381	965
1930	2888	2615		2205	2694	1204		36	42	29		63	30	13		1007	1040	1326		1167	1427	725
2219	1662	2692		1907	1016	1665		28	27	48		3	7	3		1435	1145	2003		1324	667	1287
2743	1915	2462		1545	1213	2393		39	25	41		16	5	7		1808	935	1396		1104	580	1205
2510	2326	3518		1938	2511	2192		6	13	21		8	31	57		1501	1344	1845		1361	921	1261
2575	2066	3243		2920	1550	2065		17	8	72		25	10	24		1578	1187	2127		2108	844	1193
2649	2802	2867		2741	2062	2225		101	8	98		21	15	30		1299	1944	1726		1924	866	1316
2197	2022	2882		2086	2543	1108		35	11	51		37	13	26		1363	1397	1705		1199	672	719
1592	2934	3365		1719	1197	1896		27	14	77		13	23	20		1112	1842	1640		1150	363	1032
1724	1931	1795		1870	1353	1208		82	24	54		30	26	21		816	1068	1278		1401	779	805
2804	1768	3093		2145	1372	1962		129	24	48		14	30	33		1547	778	1898		1196	727	994
2230	2757	3238		1927	1284	2096		85	54	38		1	10	23		1282	906	2014		883	902	1116
1835	2708	4139		1925	1142	1405		39	27	60		12	19	19		1074	1244	1977		1286	829	1055
2683	1666	2223		1557	2500	1587		46	53	71		10	9	7		1516	885	1615		734	1182	951
2272	1785	2190		1960	1621	1762		15	57			27	2	6		1396	576	1317		913	781	1245
3728	1103	1737		2098		1766		48	65			4		3		1577	546	1350		1394		1207
3058	1517	2594		1201		1374		28	68			7		51		1520	671	1583		675		885
2587	1333	3501		1490				13	48			16		36		1461	627	1876		936		
	2593	3140		1345					42			10		55			530	1720		961		
	960	3098		2250					15			11		37			588	1447		1374		
	2874	2192		2196					107			23					693	1713		1168		
	3452	2391		1274					130			11					859	1512		698		
		2370		2111								37						1605		1371		
				2144								27								1405		
