# Supplementary material for: Sex differences in the regulation and function of cellular immunity in Drosophila
Source: PLoS Genet. 2026 Jul 10;22(7):e1012151. doi: 10.1371/journal.pgen.1012151 (PMC13399539; doi:10.1371/journal.pgen.1012151)
Supplement: S5 Data — (PDF) [file pgen.1012151.s024.pdf]

NUCLEI								CRYSTAL CELL								PROGENITORS						
FEMALE				MALE				FEMALE				MALE				FEMALE				MALE		
collier gal4	UAS/TraF	TraF		collier gal4	UAS/TraF	TraF		collier gal4	UAS/TraF	TraF		collier gal4	UAS/TraF	TraF		collier gal4	UAS/TraF	TraF		collier gal4	UAS/TraF	TraF
3030	2552	1562		2722	2178	1520		42	27	15		12	29	15		2406	888	1207		1565	1159	714
2441	1589	1908		2864	3472	955		34	10	24		25	49	1		1582	876	1471		1484	1562	477
1830	1827	2794		2152	1781	2388		9	23	23		22	32	3		750	1016	1686		1458	578	1409
1776	2945	2306		3160	801	2049		41	21	14		9	31	16		1017	988	1501		1831	166	1265
2530	2821	1906		1449	2163	1752		31	47	16		21	63	10		1493	833	1049		749	824	965
2518	3034	2615		1930	2875	1204		11	41	29		7	58	13		1243	1720	1326		581	1016	725
3099	2851	2692		1179	1661	1665		49	74	48		19	2	3		1736	1355	2003		414	311	1287
3048	3249	2462		1514	3023	2393		63	41	41		25	4	7		1742	1356	1396		581	698	1205
3294	3258	3518		1653	2287	2192		52	18	21		13	105	57		1750	1673	1845		659	1064	1261
2188	4104	3243		1588	4903	2065		75	28	72		3	99	24		1415	827	2127		674	1528	1193
2583	2133	2867		2045	3802	2225		50	14	98		19	33	30		1568	1060	1726		1008	929	1316
1174	2168	2882		1827	3407	1108		40	32	51		39	23	26		532	1117	1705		1431	926	719
2848	3118	3365		1934	2581	1896		43	34	77		16	16	20		1315	1301	1640		1146	657	1032
2494	3406	1795		2393	2614	1208		47	61	54		19	5	21		1171	1111	1278		1642	603	805
3615	2290	3093		1990	1897	1962		101	38	48		30	18	33		1267	729	1898		1539	916	994
2086	2495	3238		1432	1863	2096		64	78	38		26	13	23		993	981	2014		665	1285	1116
2711		4139		1268	3156	1405		76		60		25	4	19		1149		1977		620	1255	1055
3163		2223		1769	3103	1587		56		71		36	8	7		848		1615		917	984	951
2638		2190		1752	1255	1762		9				9	87	6		909		1317		925	878	1245
2578		1737		2304	2555	1766		24				13	114	3		1184		1350		1231	1983	1207
3476		2594		1751		1374						7	105	51		1254		1583		913		885
4513		3501		2062								14		36		2735		1876		1188		
3219		3140		1819								17		55		2229		1720		1181		
3060		3098		2201								22		37		1955		1447		1260		
3969		2192		1768								3				2391		1713		1140		
3520		2391		2683								5				1891		1512		2095		
3998		2370		2168								8				1878		1605		1743		
4261				1254								16				1954				774		
				2370								19								1336		
				2318																1092		
				1748																1061		
