# Supplementary material for: Sex differences in the regulation and function of cellular immunity in Drosophila
Source: PLoS Genet. 2026 Jul 10;22(7):e1012151. doi: 10.1371/journal.pgen.1012151 (PMC13399539; doi:10.1371/journal.pgen.1012151)
Supplement: S6 Data — (PDF) [file pgen.1012151.s025.pdf]

NUCLEI							
FEMALE				MALE			
colliergal4	gal4/RNAi	UAS InR RNAi		colliergal4	gal4/RNAi	UAS InR RNAi	
3030	4152	3423		2722	3296	2898	
2441	4396	3902		2864	4003	2370	
1830	3065	1707		2152	2298	932	
1776	3348	3093		3160	2336	1851	
2530	3676	1808		1449	3455	2427	
2518	4376	2045		1930	3726	2977	
3099	4339	2240		1179	2616	1691	
3048	2595	2348		1514	2765	2458	
3294	2951	2463		1653	3088	1738	
2188	3032	2400		1588	3417	2392	
2583	2821	2143		2045	3383	2300	
1174	2397	3504		1827	3636	2191	
2848	3515	2594		1934	2723	1801	
2494	1914	2661		2393	3156	3054	
3615	2464	3354		1990	2725	2571	
2086	3255	4010		1432	1676	2276	
2711	2303	3364		1268	1587	1816	
3163	3419	2910		1769	2386	2359	
2638	3303	3057		1752	2989	2493	
2578	2986	2476		2304	1153	3125	
3476	3533	2344		1751	1310	2067	
4513	4324	2696		2062		2046	
3219	4028	2940		1819		1581	
3060	2496	3823		2201		2540	
3969	1773	2649		1768		1252	
3520	1952	1983		2683		1079	
3998	1216			2168		1178	
4261				1254		995	
				2370		2070	
				2318		1983	
				1748			

CRYSTAL CELL							
FEMALE				MALE			
colliergal4	gal4/RNAi	UAS InR RNAi		colliergal4	gal4/RNAi	UAS InR RNAi	
42	66	95		12	45	50	
34	51	108		25	56	60	
9	17	35		22	38	4	
41	53	70		9	32	27	
31	25	34		21	26	41	
11	27	51		7	27	40	
49	25	44		19	46	23	
63	14	11		25	62	34	
52	2	26		13	15	12	
75	35	43		3	86	23	
50	59	46		19	86	30	
40	39	70		39	62	45	
43	69	68		16	39	6	
47	53	46		19	103	17	
101	21	78		30	111	38	
64	34	64		26	35	49	
76	51	90		25	33	37	
56	49	54		36	60	40	
9	54	83		9	75	47	
24	61	25		13	16	49	
	48	15		7	0	20	
	15	17		14		38	
	6	63		17		26	
	56	46		22		22	
	36	19		3		30	
		14		5		15	
				8		3	
				16		4	
				19		13	
						21	

PROGENITORS							
FEMALE				MALE			
colliergal4	gal4/RNAi	UAS InR RNAi		colliergal4	gal4/RNAi	UAS InR RNAi	
2406	1800	1294		1565	1635	1356	
1582	1357	1267		1484	1431	1130	
750	1468	849		1458	1071	346	
1017	1583	1459		1831	1176	888	
1493	2121	1004		749	1602	713	
1243	1714	1111		581	2032	1250	
1736	2215	880		414	1134	571	
1742	1245	1079		581	1349	803	
1750	1701	1070		659	1536	1106	
1415	1984	995		674	1665	1160	
1568	2127	972		1008	2038		
532	1007	1285		1431	1910	1032	
1315	1208	1359		1146	1450	689	
1171	965	1538		1642	1644	1086	
1267	1175	1847		1539	947	1305	
993	1786	1654		665	599	846	
1149	1699	1155		620	694	688	
848	2172	1125		917	947	1003	
909	2052	1250		925	975	1006	
1184	944	1339		1231	576	1007	
1254	952	843		913	578	552	
2735	1964	1222		1188		935	
2229	1817	715		1181		735	
1955	1378	1134		1260		1052	
2391	919	883		1140		584	
1891	768	1262		2095		586	
1878	402			1743		568	
1954				774		533	
				1336		1111	
				1092		907	
				1061			
