# Supplementary material for: Sex differences in the regulation and function of cellular immunity in Drosophila
Source: PLoS Genet. 2026 Jul 10;22(7):e1012151. doi: 10.1371/journal.pgen.1012151 (PMC13399539; doi:10.1371/journal.pgen.1012151)
Supplement: S7 Data — (PDF) [file pgen.1012151.s026.pdf]

NUCLEI								CRYSTAL CELL								PROGENITORS						
FEMALE				MALE				FEMALE				MALE				FEMALE				MALE		
colliergal4	gal4/Ca-InR	UAS Ca-InR		colliergal4	gal4/Ca-InR	UAS Ca-InR		colliergal4	gal4/Ca-InR	UAS Ca-InR		colliergal4	gal4/Ca-InR	UAS Ca-InR		colliergal4	gal4/Ca-InR	UAS Ca-InR		colliergal4	gal4/Ca-InR	UAS Ca-InR
3030	4132	2479		2722	3659	1140		42	37	39		12	37	38		2406	1461	1636		1565	1919	571
2441	3701	3683		2864	2864	2655		34	55	35		25	51	46		1582	1268	2405		1484	1664	1343
1830	4610	2477		2152	2567	2510		9	38	11		22	47	22		750	2428	1637		1458	1110	1501
1776	3573	4241		3160	1617	3941		41	41	52		9	43	27		1017	1371	1338		1831	461	1528
2530	3789	3941		1449	1360	1442		31	36	56		21	21	37		1493	2339	1784		749	508	1002
2518	4164	2809		1930	2384	1700		11	29	66		7	28	10		1243	2486	1428		581	814	1057
3099	3658	3290		1179	2259	1053		49	10	129		19	80	8		1736	2387	1302		414	551	430
3048	2603	3300		1514	1643	946		63	17	62		25	58	4		1742	1026	1800		581	561	644
3294	3217	2612		1653	2783	1721		52	29	75		13	19	27		1750	2256	1404		659	1442	974
2188	2882	3155		1588	3309	2762		75	12	45		3	28	23		1415	1560	931		674	2211	1870
2583	3634	2747		2045	2562	2936		50	44	50		19	26	21		1568	2156	952		1008	1278	1666
1174	2420	3867		1827	1604	2362		40	19	54		39	19	75		532	1298	1299		1431	971	1026
2848	4590	3397		1934	2461	2990		43	11	54		16	24	13		1315	2125	1447		1146	1490	1908
2494	3909	4225		2393	3369	2616		47	8	104		19	62	0		1171	1665	1575		1642	1339	1833
3615	1993	2699		1990	2510	1706		101	51	56		30	3	8		1267	1114	1740		1539	1605	852
2086	2498	3322		1432	3204	2707		64	54	16		26	2	6		993	1445	1552		665	2029	1512
2711	3187	2917		1268	1190	2581		76	44	29		25	15	39		1149	1102	1871		620	780	1600
3163	2974	2583		1769	2515	1794		56	15	4		36	42	32		848	1350	1812		917	1401	615
2638	2071	2540		1752	3074	3563		9	11	12		9	41	29		909	749	1901		925	1923	1877
2578	2650	2185		2304	2984	2215		24	54	34		13	60	23		1184	1176	1214		1231	1222	1154
3476	3477	2436		1751		2241			22	45		7		39		1254	1232	1115		913		1170
4513	2126	3295		2062					17	45		14				2735	900	1356		1188		
3219	3298	2932		1819					16	26		17				2229	969	1870		1181		
3060	3984	1721		2201					1	24		22				1955	1505	953		1260		
3969	2730	2471		1768					3	64		3				2391	1069	827		1140		
3520	2285			2683					41			5				1891	1371			2095		
3998	2943			2168					49			8				1878	2004			1743		
4261	2307			1254								16				1954	1654			774		
	3209			2370								19					1972			1336		
	4532			2318													2501			1092		
				1748																1061		
