# Supplementary material for: Sex differences in the regulation and function of cellular immunity in Drosophila
Source: PLoS Genet. 2026 Jul 10;22(7):e1012151. doi: 10.1371/journal.pgen.1012151 (PMC13399539; doi:10.1371/journal.pgen.1012151)
Supplement: S8 Data — (PDF) [file pgen.1012151.s027.pdf]

E COLI INFECTION																		
NUCLEI					CRYSTAL CELL					PROGENITORS					PLASMATOCYTES			
control F	control M	infectionF	infectionM		control F	control M	infectionF	infectionM		control F	control M	infectionF	infectionM		control F	control M	infectionF	infectionM
3313	1971	2998	1831		51	22	85	18		1778	1185	1391	1202		233	385	295	139
3004	2762	2789	1879		47	20	76	8		1744	1205	982	1256		460	427	165	232
2349	1094	3458	1859		47	3	184	46		1407	680	561	998		168	187	368	357
2623	1967	3444	1372		120	4	99	21		1296	1187	1061	756		304	319	537	133
2421	1964	4632	1389		42	3	196	24		1156	1227	1439	656		451	175	293	321
2206	1157	3691	2677		94	5	209	35		1225	815	1352	715		184	51	279	311
4138	1197	3911	1750		108	22	269	31		2195	796	1077	853		204	247	488	343
3063	2573	2926	1548		36	21	119	33		1438	667	955	1022		346	246	429	434
3523	1649	3370	2118		38	19	169	90		1496	629	889	1581		177	230	252	543
2847	1124	2261	2371		62	42	33	68		1105	941	1162	751		63	106	223	155
2468	1383	2697	2751		29	41	62	50		1174	663	1139	925		106	100	437	407
2480	1491	3445	2707		40	24	232	99		1113	895	646	1114		341	198	257	773
1915	1356	2101	2691		52	40	92	100		956	1144	1899	1565		191	363	707	308
2194	1793	3203	1820		42	85	165	47		1135	1190	1569	1266		297	312	269	278
2596	2218	2917	2949		43	48	311	52		1235	803	1250	918		73	144	767	579
2405	2408	5120	2790		47	25	99	92		1238	793	1049	692		309	76	626	329
3359	1503	2939	2201		31	42	49	70		1574	923	1704	862		221	237	454	189
1810	1582	3294	1518		49	27	149	9		988	877	2473	766		324	304	495	284
2451	1619	3857	1063		30	17	286	8		962	1015	1695	941		165	377	204	
1281	1357	3652	1579		31	11	80	39		680	1028	1272	1138		107	250	144	
1490	2093	4350	1680		46	10	103	21		704	1231		1204		227	146	238	
1645	2975	2936	1719		32	11	38	31		931	1031		978		295	184	396	
1840	2968	2923	2387		25	45	105	55		976	1216				264	43	244	
2435	2839		2112		45	12		30		1042					128	249	365	
2434	2715		2684		60	25		36		1050						167	423	
3009	2643				73	21				1765						183		
2751					46					1401						262		
3735					45					1018								
3414					61					1340								
2052					52													
2371					55													
2030																		
2570																		
