# Supplementary material for: Sex differences in the regulation and function of cellular immunity in Drosophila
Source: PLoS Genet. 2026 Jul 10;22(7):e1012151. doi: 10.1371/journal.pgen.1012151 (PMC13399539; doi:10.1371/journal.pgen.1012151)
Supplement: S9 Data — (PDF) [file pgen.1012151.s028.pdf]

P. CAROTOVORUM INFECTION																		
NUCLEI					CRYSTAL CELL					PROGENITORS					PLASMATOCYTES			
control F	control M	infectionF	infectionM		control F	control M	infectionF	infectionM		control F	control M	infectionF	infectionM		control F	control M	infectionF	infectionM
3313	1971	2475	2382		51	22	20	56		1778	1185	1593	957		233	385	228	269
3004	2762	3024	2821		10	20	42	49		1744	1205	1770	1536		460	427	478	248
2349	1094	3194	1886		47	3	59	45		1407	680	1565	704		168	187	377	285
2623	1967	3867	2307		47	4	136	21		1296	1187	1752	952		304	319	366	281
2421	1964	2813	2427		120	3	129	46		1156	1227	1420	835		451	175	471	382
2206	1157	3504	2837		42	5	140	51		1225	815	1872	1221		184	51	278	249
4138	1197	3322	2702		3	22	90	39		2195	796	1288	889		204	247	360	108
3063	2573	2937	1969		94	21	42	55		1438	667	1275	777		346	246	285	159
3523	1649	2848	1894		108	19	72	66		1496	629	1348	1005		177	230	197	349
2847	1124	2966	1779		36	42	74	59		1105	941	1495	680		63	106	358	166
2468	1383	2296	2140		38	41	12	23		1174	663	1284	1220		106	100	437	667
2480	1491	1651	1698		62	24	39	11		1113	895	791	1025		341	198	167	247
1915	1356	2979	1441		29	40	101	18		956	1144	593	570		191	363	213	325
2194	1793	2829	1702		40	85	44	38		1135	1190	1543	824		297	312	438	191
2596	2218	2398	2534		52	48	121	36		1235	803	1553	1026		73	144	266	110
2405	2408	4163	1237		42	25	103	20		1238	793	1687	715		309	76	277	106
3359	1503	4032	1869		43	42	96	27		1574	923	2198	930		221	237	152	273
1810	1582	2375	1948		47	27	74	39		988	877	775	1045		324	304	208	
2451	1619	2542	2355		31	17		46		962	1015	1312	717		165	377	207	
1281	1357				49	11				680	1028				107	250	218	
1490	2093				30	10				704	1231				227	146	105	
1645	2975				31	11				931	1031				295	184	216	
1840	2968				46	45				976	1216				264	43		
2435	2839				32	12				1042					128	249		
2434	2715				25	25				1050						167		
3009	2643				45	21				1765						183		
2751					60					1401						262		
3735					73					1018								
3414					46					1340								
2052					45													
2371					61													
2030					52													
2570					55													
