# Supplementary material for: Sex differences in the regulation and function of cellular immunity in Drosophila
Source: PLoS Genet. 2026 Jul 10;22(7):e1012151. doi: 10.1371/journal.pgen.1012151 (PMC13399539; doi:10.1371/journal.pgen.1012151)
Supplement: S8 File — (PDF) [file pgen.1012151.s009.pdf]

| | Female Genes PSC | | | | | Male Genes PSC | | | |
|---|---|---|---|---|---|---|---|---|---|
| | GO or pathway | ID | Adjusted p-value | Genes | | GO or pathway | ID | Adjusted p-value | Genes |
| | peptide receptor activity | GO:0001653 | 0.00249 | CCKLR-17D3,CG31183,TKR99D,DH31- | | regulation of axon guidance | GO:1902667 | 0.0235 | PK,NETA,NETB |
| | neuropeptide receptor activity | GO:0008188 | 0.03917 | CCKLR-17D3,TKR99D,DH31-R | | cell periphery | GO:0071944 | 0.0101 | LCP65AB1,CPR47EC,LCP65AB2 |
| | G protein-coupled receptor activity | GO:0004930 | 0.04506 | CCKLR-17D3,TKR99D,DH31-R,MTHL7 | | extracellular matrix | GO:0031012 | 0.0156 | LCP65AB1,CPR47EC,LCP65AB2,DPY,NETA,NETB |
| | Neuroactive ligand-receptor interaction | KEGG:04080 | 0.02683 | CCKLR-17D3,TKR99D,DH31-R | | external encapsulating structure | GO:0030312 | 0.0295 | LCP65AB1,CPR47EC,LCP65AB2 |
| | | | | | | extracellular region | GO:0005576 | 0.0343 | LCP65AB1,CG34227,LCP65AB2[illegible] |
| | | | | | | Longevity regulating pathway - multiple species | KEGG:04213 | 0.0087 | ILP6,HSP70BB,AC13E |
| | | | | | | | | | |
| | | | | | | | | | |
| | | | | | | | | | |
| | | | | | | | | | |
| | | | | | | | | | |
