# Supplementary material for: Sex differences in the regulation and function of cellular immunity in Drosophila
Source: PLoS Genet. 2026 Jul 10;22(7):e1012151. doi: 10.1371/journal.pgen.1012151 (PMC13399539; doi:10.1371/journal.pgen.1012151)
Supplement: S10 File — We ran this analysis both with and without the ‘overall’ category, which refers to the comparison of total male and female lymph gland cells. (PDF) [file pgen.1012151.s011.pdf]

Female DEGs by Zone

PSC	CC	MZ	PSC and MZ	PSC and CCs	CC and MZ	PSC and CC and MZ
CG12112	CG32816	CecC	CG15784	CG3939	Arpc3B	Karl
Idgf4	CG17778	CecA2	CR43211		CG11590	MCTS1
CG15365	Gasp	CG2444			CG14434	lncRNA:CR40469
CG14855	CG30148	CecB			CG43133	
CG13369	CG10527	CecA1			CG4593	
form3	alpha-Est4	CG5004			Sxl	
CCKLR-17D3	CG14629	AnxB11				
snRNA:U2:14B	CG14270	Gclc				
CG9220	lncRNA:CR45530	CG33225				
Hers	CrebB	CG34331				
CG31183	Galphai	Atf3				
septin 4	Tep1	CG17982				
CG17321	CG7453	CG3781				
Dl		CG7322				
Vps2		Chrac-16				
comm2		hpRNA:CR32207				
NimB5		unc-13				
lncRNA:CR33218		rdgB				
CG10508		CR43212				
TkR99D		CG31821				
CG14516		CG9733				
CG8369		lncRNA:CR42868				
lncRNA:CR44933		lncRNA:CR30009				
comm		CG12056				
l(2)01289		Tsp3A				
asRNA:CR45912		CG17109				
Ace		CG3587				
Dh31-R		Fas3				
CG17121		Aven				
Cpr5C		CG3038				
mthl7		CR43214				
CG34002		DIP-alpha				
CG6287		CG32641				
ImpL2		lncRNA:CR43264				
CG43759		Dab				
		lncRNA:CR45517				
		wus				
		Wsck				
		Cht2				
		CG10804				
		CG31689				
		CR43217				
		His2B:CG17949				
		RNaseP:RNA				
		Crg-1				
		CR43215				

Male DEGs by Zone

PSC	CC	MZ	PSC and MZ	CC and MZ	PSC and CC	PSC and CC and MZ
Lcp65Ab1	jv	comt		CG15739	Ilp6	lncRNA:roX1
Cpr47Ec	CG11318	CG3176		CG6999		lncRNA:roX2
CG34227	trol	CG32706		CG7997		
Lcp65Ab2	CG9119	CG32625		D2hgdh		
CG13403	asRNA:CR44992	CG5273		lncRNA:CR32582		
dpy	E23	CG11655		lncRNA:CR45979		
Ets98B	snRNA:U6:96Ac	CG43867		msl-2		
fj	CG34232	CG16957				
SKIP	Jheh3	CR18166				
slf	mthl14	CG4586				
CG9449	Hk	CG3568				
CG34398	CG2017	CG15317				
pk	lncRNA:CR43051	mof				
ovo	Tsp42Er	CkIIalpha-i3				
lncRNA:CR43432	CG5418	nkd				
mirr	lncRNA:CR44895	CG1998				
CG8526	Gp150	CG5167				
CG46339	Vav	corolla				
CG34370	asRNA:CR46128	wde				
NetA	AsnS	GstT3				
Picot	CG34120	CG43759				
axed	PCNA2	CG43051				
NetB	CG11576	Hsp70Aa				
CG8547	lncRNA:CR44380	CG6762				
Hsp70Bb	CG42240	CG4239				
Ca-alpha1D	mwh	ewg				
Npc2a	lncRNA:CR44757	sdk				
CG1113	CG6805	CG32817				
rdgA	CG9008					
CG32280						
lncRNA:CR45959						
bark						
CG13284						
CG9095						
CG2781						
ko						
GILT2						
CG11134						
Ac13E						
Pgant2						

Female DEGs by Zone including overall

PSC	PSC and CC	CC	MZ	MZ and CCs	PSC and MZ	PSC and MZ and CC	PSC and MZ and overall	MZ and overall	overall	MZ and CC and overall	CC and overall	PSC and overall	PSC and CC and overall	PSC and CC and MZ and overall
CG12112	CG3939	CG17778	CG2444	CG11590			CG15784	CG2444	CG4301	Arpc3B	CG32816	Cpr5C		Karl
Idgf4		Gasp	Gclc				CR43211	Gclc	NimC1	CG14434				MCTS1
CG15365		CG30148	CG34331					CG34331	sano	CG43133				lncRNA:CR40469
CG14855		CG10527	Atf3					Atf3	dpr14	CG4593				
CG13369		alpha-Est4	CG17982					CG17982	CG34054	Sxl				
form3		CG14629	CG7322					CG7322	mthl4					
CCKLR-17D3		CG14270	Chrac-16					Chrac-16	Tsp42Ed					
snRNA:U2:14B		lncRNA:CR45530	hpRNA:CR32207					hpRNA:CR32207	prage					
CG9220		CrebB	rdgB					rdgB	Rip11					
Hers		Galphai	lncRNA:CR42868					lncRNA:CR42868	CG9981					
CG31183		Tep1	lncRNA:CR30009					lncRNA:CR30009	Sox15					
septin 4		CG7453	CG12056					CG12056	CG1840					
CG17321			CG17109					CG17109	lawc					
Dl			Fas3					Fas3	png					
Vps2			Aven					Aven	RhoGAP18B					
comm2			lncRNA:CR43264					lncRNA:CR43264						
NimB5			Dab					Dab						
lncRNA:CR33218			lncRNA:CR45517					lncRNA:CR45517						
CG10508			Wsck					Wsck						
TkR99D			CG31689					CG31689						
CG14516			CR43217					CR43217						
CG8369			His2B:CG17949					His2B:CG17949						
lncRNA:CR44933			RNaseP:RNA					RNaseP:RNA						
comm														
hlk														
asRNA:CR45912														
Ace														
Dh31-R														
CG17121														
mthl7														
CG34002														
CG6287														
ImpL2														
CG43759														

Male DEGs by Zone including overall

PSC	PSC and CC	CC	MZ	MZ and CCs	PSC and MZ	PSC and MZ and CC	PSC and MZ and overall	MZ and overall	overall	MZ and CC and overall	CC and overall	PSC and overall	PSC and CC and overall	PSC and CC and MZ and overall
CG12112	CG3939	CG17778	CG2444	CG11590			CG15784	CG2444	CG4301	Arpc3B	CG32816	Cpr5C		Karl
Idgf4		Gasp	Gclc				CR43211	Gclc	NimC1	CG14434				MCTS1
CG15365		CG30148	CG34331					CG34331	sano	CG43133				lncRNA:CR40469
CG14855		CG10527	Atf3					Atf3	dpr14	CG4593				
CG13369		alpha-Est4	CG17982					CG17982	CG34054	Sxl				
form3		CG14629	CG7322					CG7322	mthl4					
CCKLR-17D3		CG14270	Chrac-16					Chrac-16	Tsp42Ed					
snRNA:U2:14B		lncRNA:CR45530	hpRNA:CR32207					hpRNA:CR32207	prage					
CG9220		CrebB	rdgB					rdgB	Rip11					
Hers		Galphai	lncRNA:CR42868					lncRNA:CR42868	CG9981					
CG31183		Tep1	lncRNA:CR30009					lncRNA:CR30009	Sox15					
septin 4		CG7453	CG12056					CG12056	CG1840					
CG17321			CG17109					CG17109	lawc					
Dl			Fas3					Fas3	png					
Vps2			Aven					Aven	RhoGAP18B					
comm2			lncRNA:CR43264					lncRNA:CR43264						
NimB5			Dab					Dab						
lncRNA:CR33218			lncRNA:CR45517					lncRNA:CR45517						
CG10508			Wsck					Wsck						
TkR99D			CG31689					CG31689						
CG14516			CR43217					CR43217						
CG8369			His2B:CG17949					His2B:CG17949						
lncRNA:CR44933			RNaseP:RNA					RNaseP:RNA						
comm														
hlk														
asRNA:CR45912														
Ace														
Dh31-R														
CG17121														
mthl7														
CG34002														
CG6287														
ImpL2														
CG43759														
