# Supplementary material for: Sex differences in the regulation and function of cellular immunity in Drosophila
Source: PLoS Genet. 2026 Jul 10;22(7):e1012151. doi: 10.1371/journal.pgen.1012151 (PMC13399539; doi:10.1371/journal.pgen.1012151)
Supplement: Attachment — Submitted filename: Response to Reviewers.docx [file pgen.1012151.s030.docx]

Dear Dr. Wapner,

We enclose for your consideration our response to the reviews of our manuscript entitled “**Sex differences in the regulation and function of cellular immunity in Drosophila**”. We are grateful for the very helpful and constructive feedback of the reviewers and have significantly improved the manuscript based on their feedback. In our revised manuscript, we added new data and experiments, reanalyzed data, as well as expanded, revised, and improved the text in accordance with the reviewers’ input. Although we were not able to completely answer every question raised by the reviewer, due to technical challenges and time limitations, we put in tremendous effort to address every single of their questions and we will be willing to engage the reviewers further if they still have further concerns.

Some of the major improvements to the manuscript include:

- Addressed questions about the PSC numbers and normalization method we employed raised by reviewer 1.
- Provided better characterization, controls, and explanation of data using the DomeMesoGal4 and DomeMesoGFP transgenes (requested by reviewers 1 and 3)
- Added analysis for the NimC gene to the single-cell RNA Data (requested by reviewers 1 and 3).
- Performed a more comprehensive analysis of sex differences in other clusters (including propl, iz, and pl) (requested by reviewers 1) and improved the description of this section as commented on by reviewer 3.
- Added numerous other clarifications, changes, and improvements in line with reviewer suggestions.

A detailed response to each reviewer’s comment is provided below. We hope that the reviewers find the revised manuscript improved and that is now suitable for publication in PLOS Genetics.

Best Regards,

Guy Tanentzapf + Alexandra Dvoskin

**Response to reviewer #1:**

*1. The authors report raw PSC numbers that are significantly higher (68±22 for males and 91±24 for females) than those reported by other studies (typically 30-40 cells per lobe). Indeed, in lines 126-127, the authors define the PSC as “a group of a few dozen cells”. Why are their PSC numbers reported throughout the paper significantly higher? If this is simply a difference in analysis methods, this should be noted and explained in the text to give clarity to the reader and so as not to confuse the field.*

The reviewer is correct to note that the values we report differ from other published values. To investigate this point in detail we went back and compared the values listed in other publications from our lab to those of in our present manuscript. We found the values from previous papers from our lab do fluctuate somewhat based on genetic background and growth conditions but hovered between 40-60, a bit higher than the 30-40 as the reviewer claims (for example In Ho et al, (2021) we reported that we observed an average of 45.28 ± 10.97 cells (n = 21 primary lobes)). This analysis did however confirm the reviewer’s suspicion that numbers in our present study are higher than those previously reported. We hypothesized that this may be due to differences between how PSC numbers have previously been assessed by us and others and what we did in our current manuscript. Specifically, in this manuscript we counted PSC cells based on stainings with Antp antibody from DSHB. In the past we, and others, used other strategies to mark PSC cells such as driving UAS-GFP with Antp-Gal4 or Collier-Gal4. To check if this might underlie the differences in values we stained flies from a cross of Collier-Gal4 to UAS-GFP and co-stained with the Antp antibody we employed. Our results (see below) show that indeed PSC counts with Antp staining of UAS-Gal4 flies crossed to collier-GFP result in numbers that were higher than when we count GFP positive cells. Nonetheless this difference accounted for ~15-20 more cells which was not sufficient to explain all of the difference between previous counts and the kind of numbers we got for the w^-^; tep4-Gal GFP. We propose that the higher numbers we observed were due to a combination of differences in both counting method and genotype. We have now added the comment below to the results (page 8 lines 163-170) and include the figure below as supplemental figure 1 to note that our numbers differ from previous reports and speculate on why.

“We noted that these numbers of PSC cells we recorded were higher than the numbers we and other groups previously reported (for example we reported ~45 PSC cells in Ho et al, 2021). This could partly be explained due to differences in methodology, as we used an antibody staining against Antp to do our PSC counts (Supplementary Figure 1), while others used different methods. However, methodology alone did not account of for all of these differences and may represent genotype specific variation”

**Supplemental figure 1- Differences between collier+ and Antp+ cell counts** Total cell count for both collier+ PSC cells (expressing GFP through *collier-gal4*) as well as Antp+ cells (stained with Antp antibody) in males and females. A significant difference was found in females between the two cell types (p=0.0029). A significant difference was found in males between the two cell types (p=0.0003).

*2. The authors show that crystal cell numbers differ significantly between males and females even after normalizing to body weight. Would normalizing crystal cell numbers to the total lymph gland cell numbers still reveal a difference between sexes? Crystal cell numbers are known to be affected by the lymph gland size, and for this reason, most studies usually report crystal cell percentage rather than raw counts. This analysis could reveal whether the reported correlation between crystal cell number and lymph gland size is similar between the sexes or due to inherent differences in lymph gland size between the sexes.*

That is an excellent question and one we gave some thought to. The simple answer is that it does not matter how we normalise, whether to body size or number of cells in the lymph gland the results are the same. For an illustration please see figure below which we now included in Supplementary figure 4 and is discussed in the text on page 10 and lines 209-211.

**Supplemental figure 4- Crystal cell counts corrected for body and lymph gland size** (A) Raw cell counts for crystal cells in *w1118 x tep4GFP* male and female larvae show a significant difference (p<0.0001). (B) Total cell counts for crystal cells in *w1118 x tep4GFP* male and female larvae, corrected for body size, show a significant difference (p=0.0056). (C)Total cell counts for crystal cells in *w1118 x tep4GFP* male and female larvae, corrected for total lymph gland size (total nuclei count for each lobe corresponding to crystal cell number data point), show a significant difference (p=0.0011).

*3. Lines 163-167 say that the authors labeled progenitors with domeMESO GFP, but the figure and figure legend for Figure 1 indicate that the progenitors are labeled by Tep4gal4; UAS-GFP. This should be clarified and corrected in the text and/or figure legends.*

We thank the reviewer for pointing out this oversight. This has now been corrected.

*4. In fact, throughout the manuscript, the authors use Tep4 and domeMESO drivers interchangeably. However, it is well established that Tep4 is expressed only in core progenitors, whereas domeMESO is expressed in both core and distal progenitors (PMID: 32243888). Several studies (including those from the authors: PMID: 34713801) have shown that genetic manipulations with these drivers can yield different results, especially as these populations are in different stages of the cell cycle and to differentially respond to signaling pathway manipulations (e.g., PMID: 38866012 ; PMID: 39466810). For this reason:*

The reviewer is correct to point out that we used both drivers and failed to delineate the difference between the populations labelled by each marker. We have now carried out additional analysis as suggested by the reviewer and this is included in figure 1 (and mentioned in the results section on page 8 lines 169-175. This analysis shows similar sex differences when either driver is used.

*a. For Figure 1, I recommend assessing the number of domeMESO-positive progenitors (as the text mentions) to determine whether they differ significantly between males and females, similar to the analysis for Tep4 progenitors (presumably what was done in Figure 1).*

This analysis (shown above) is included in the modified figure 1 (shown above)

*b. The difference between the populations marked by Tep4 and domeMESO should be explained in the text, clearly stating that when using Tep4, these are core progenitors. In contrast, when using domeMESO, the authors can use the term 'progenitors'. And the text describing the results from these figures should be double-checked for accuracy to confirm the correct driver is referenced.*

This is a good suggestion, we have now added this requested clarification throughout the text.

*5. For scRNASeq analysis, why weren’t sex differences compared, or results shown for comparisons between proPL, IZ, and PL clusters? The authors noted (lines 200-202) that these clusters were particularly different between sexes in the UMAP, so I am curious why these were not compared. These comparisons should be included for completeness, and if no DEGs were identified, this should be reported by the authors.*

*a. Interestingly, NimC1 (the antigen for P1 antibody, which the authors use for Plasmatocyte staining and is a marker for PL) was identified as a top overall gene differentially expressed between sexes (in the supplemental table). The authors should consider including it in the bubble plot for Figure 2A.*

Thank you for this helpful suggestion. We have added a more complete analysis of the sex differences in the other clusters (including proPL, IZ, and PL) to Supplementary file 1 and updated Figure 2B to include data from the additional clusters. This additional analysis is described in the results section (page 11 lines 260-286). As a result of this analysis, we have identified additional differentially expressed genes (DEGs) that show sex differences (Supplementary file 1 – Table 1), performed gene set enrichment for the DEGs in these other clusters (Supplementary file 1-Table 2), highlighted some of the interesting DEGs found in these other clusters (Figure 2B; Supplementary file 1 – Table 3), and mapped which DEGs overlap between the clusters (Supplementary file 1-Table 4). For example, one finding that resulted from this additional analysis is that male IZ cells are enriched for many DNA replication genes and protein folding chaperones (Supplementary file 1-Table 2). Overall, we believe that including this additional analysis strengthens the paper.

a. Thank you for the suggestion, we have now added NimC1 to the bubble plot in Figure 2B. We did not initially include NimC1 in the bubble plot, as it is not highly expressed overall, but the enrichment of NimC 1 in the female proPL and PL clusters is apparent in the updated bubble plot in Figure 2B. We agree that the differential expression of NimC1 across male and female cells is interesting, especially given the importance of NimC1 in plasmatocyte function. Therefore, we have now included NimC1 in Supplementary file 1-Table 3 as an ‘Interesting DEG’.

*6. I am not sure I agree with the author’s conclusions about the results from feminizing the progenitors in Figure 5. When comparing males and females in tep4gal4>TraF for nuclei count (Figure 5B), crystal cell count (Figure 5F) and progenitor count (Figure 5J), all of these are no longer significantly different between males and females and thus suggest that feminizing the females and males reverses the sex-bias in these numbers. Furthermore, the interaction constants for nuclei and progenitors are significant in the feminized progenitors experiments. This suggests that indeed some sex differences in the lymph gland are mediated by the progenitors. But I would agree that sex differences in crystal cell number in the lymph gland are not mediated by the Tep4+ progenitors.*

The reviewer asked a good question and we indeed thought a lot about this point and decided to soften our conclusions from the previous version. Overall, we agree that a less rigorous approach could find an effect of feminizing the progenitors but we wanted to be consistent, and if anything err on the side of caution. In other words our approach in interpreting the data was deliberately very conservative.

We sought to be as rigorous as possible in how we interpreted the results and set a high threshold to accept an effect as real. In particular the thresholds that needed to be met to deem a result as meaningful was observing BOTH the multiple comparisons across genotypes showing a significant p value, as well as the sex:genotype interaction being significant. When it came to the feminization of the progenitors, these standards were not fully met.

First, we noted a significant sex:genotype interaction for lymph gland size as well as progenitor numbers. This was intriguing but upon closer inspection neither of these results failed to meet our rigorous standards.

For the nuclei counts indicating lymph gland size (Fig 5A), while we do see a significant sex:genotype interaction, we do not see a significant increase or decrease in males or females when looking at the multiple comparisons. This means we cannot confirm that transgenes are active in the progenitors in this instance so are weary of using these experiments as a basis for a strong conclusion. A similar situation exists from crystal cell numbers upon Tra^F^ expression.

For the progenitor counts (Figure 5I), we again saw a significant sex:genotype interaction. However, this result did not meet another one of our required thresholds. In particular, one of the confounding factors in our experiments was the impact of genetic background. To control for that we enforced a threshold where we required the effect to be due to changes induced by feminization of males which is particularly relevant for figure 5I. Because females already express Tra^F^ , observing increase or decrease of cell counts with the overexpression of the Tra^F^ construct in females to a degree that impacts sex differences does not suggest a meaningful elimination of sex differences.

In other words, we failed to meet our desired threshold for all three of the values we assayed for when we feminized progenitors in males (lymph gland size, crystal cell number, progenitor numbers).

We attempted to explain this better and added a section to the discussion in the revised manuscript on page 17 lines 395-412. We also softened our conclusion to state that all we did was fail to find conclusive proof of an effect in these experiments. This leaves the possibility that a different set of experiments could provide more conclusive evidence at a later date. If the reviewer thinks that in this particular instance we should soften our conclusions more we are willing to consider this further.

*a. This data may be difficult to interpret because there are smaller differences between males and females in the tep4-gal4 only controls, but this underscores the need to repeat these experiments with domeMESO-GAL4 to truly conclude that “Sex differences in the lymph gland are not mediated by progenitors” (line 330). Thus, in Figure 5, I suggest using the domeMESO-GAL4 driver to feminize progenitors in addition to Tep4, as this might yield different results*

Upon feminization of *domeMESO+* progenitors using *domeMESO gal4*, we see that there is no change in nuclei, crystal cell, or domeMESO+ progenitors count. We see the same issue as with *tep4 gal4,* where there are such small differences between the males and females in the *domeMESO gal4* only controls in all three cell types. But looking at our rigorous thresholds we held for these feminization experiments, we cannot conclude that *domeMESO+* or *tep4+* progenitors are responsible for mediating sex differences in the lymph gland(page 17, lines 405-412).

**domeMESO gal4 Feminization-** (A) Raw cell counts for total number of nuclei. A 2 way ANOVA, Tukey’s test showed: no significance between *domeMESO gal4* control females and *domeMESOgal4* feminized females (p=0.7209), a significant difference between *UAS TraF* control females and *domeMESOgal4* feminized females (p=0.0005), no significance between *domeMESO* control males and *domeMESO* feminized males (p=0.9949), and no significance between *UAS TraF* control males and *domeMESO* feminized males (p=0.2093). The genotype:sex interaction constant was not significant (p=0.1876). (B) Raw cell counts for total number of nuclei comparing females and males of each group. A 2 way ANOVA, Tukey’s test showed: no significant difference between *domeMESO gal4* control females and males (p=0.4752), no significant difference between *domeMESO gal4* feminized females and males (p=0.1425), and a significant difference between *UAS TraF* control females and males (p<0.0001). (C) Raw cell counts for total number of crystal cells. A 2 way ANOVA, Tukey’s test showed: no significant difference between *domeMESO gal4* control females and *domeMESO gal4* feminized females (p=0.2599),no significant difference between *UAS TraF* control females and *domeMESO gal4* feminized females(p>0.9999), no significant difference between *domeMESO gal4* control males and *domeMESO gal4* feminized males (p=0.2422), and no significant difference between *UAS TraF* control males and *domeMESO gal4* feminized males (p=0.9452). The genotype:sex interaction constant was not significant (p=0.7756). (D) Raw cell counts for total number of crystal cells comparing females and males of each group. A 2 way ANOVA, Tukey’s test showed: no significant difference between *domeMESO gal4* control females and males (p=0.1782), no significant difference between *domeMESO gal4* feminized females and males (p=0.3322), and a significant difference between *UAS TraF* control females and males (p=0.0032). (E) Raw cell counts for total number of *domeMESO+* progenitors. A 2 way ANOVA, Tukey’s test showed: no significant difference between *domeMESO gal4* control females and *domeMESO gal4* feminized females (p>0.9999), a significant difference between *UAS TraF* control females and *domeMESO gal4* feminized females (p<0.0001), no significant difference between *domeMESO gal4* control males and *domeMESO gal4* feminized males (p=0.9393), and a significant difference between *UAS TraF* control males and *domeMESO gal4* feminized males (p=0.0001). The genotype:sex interaction constant was significant (p=0.0252). (F) Raw cell counts for total number of *domeMESO+* progenitors comparing females and males of each group. A 2 way ANOVA, Tukey’s test showed: no significant difference between *domeMESO gal4* control females and males (p=0.5104), no significant difference between *domeMESO gal4* feminized females and males(p=0.1194), and a significant difference between *UAS TraF* control females and males (p<0.0001).

*b. Note: the gal4 insertion in Tep4 (from Kyoto DGRC) may disrupt gene function (hence why it cannot go homozygous), and so the smaller differences between males and females in this background may be biologically relevant.*

We thank the reviewer for raising this interesting point. We repeated the feminization of progenitors experiment with the domeMESOgal4 (Supplementary figure 5) and found a similar trend to that we saw using tep4gal4, this argues that the issue raised by the reviewer does not impact our findings in a meaningful way..

*c. Similarly, in Figure 5, I recommend quantifying Tep4-positive core progenitor numbers, as these may show significant differences. Indeed, if that is what was used in Figure 1, there were significant differences in Tep4-positive core progenitors in wildtype and so quantifying those numbers in addition to the domemeso+ progenitors in the feminized backgrounds is essential for making conclusions about the role of progenitors in sex differences.*

It is our hope that repeating the experiments with the DomeMeso-Gal4 addresses these concerns.

*7. The authors state that “it should become a standard practice to consider sex as a key variable for all phenotypic characterization of the lymph gland.” But the authors also noted that controlling for body weight eliminates most raw count differences for nuclei, PSC, and progenitors (and that there were no differences for plasmatocytes). Most studies do not report raw counts but rather normalized counts to lymph gland size (raw count/total nuclei). Figure 1 should include sex-specific “normalized” values, as this would give important context to interpreting many past experiments.*

The reviewers raise a really great point. We went ahead and tested whether reporting normalized counts to lymph gland size (raw count/total nuclei) effectively corrects for size differences between males and females. This analysis, shown in Supplemental Figure 3, illustrates that indeed normalized counts correct for sex specific differences. We modified our results and discussion on page 9 lines 201-204 to explicitly mention this point as we agree this provides important context.

*a. Another important point the authors may want to point out is that earlier work using domeless-GAL4 to drive manipulations in the progenitors (or other GAL4s that are lethal in males) only looked at these manipulations in female animals, as it would be impossible to drive the GAL4 in males as it is lethal. So there could be an important and interesting missing body of data on the impact of some of the same signaling pathways mentioned in the introduction that is yet to be explored (and discrepancies between results from manipulations that use dome-GAL4 and domeMESO-GAL4, which is not lethal in males, could suggest sex-specific effects and would make it that much more essential to take sex into account when analyzing and interpreting results)*

That is again a great point which we now mention in the discussion on page 25 Lines 576-578.

*Minor Questions:
1. For the insulin pathway experiments, have the authors considered testing additional pathway members, such as expressing a constitutively active form of PI3K or knocking down the kinase Akt?*

This is an excellent suggestion and in our future experiments that will make up a big part of our planned future follow up work.

*2. How do PSC feminized males respond to infection with P. carotovorum and E. coli?*

This is also a key experiment in our future to do list.

*Minor Comments:
1. In all figures except for the last two, the labels for nuclei, crystal cells, and progenitors should be in larger, brighter font to improve visibility.*

Thanks for this suggestion. We have made the font larger as requested.

*2. In Figure 6, authors should update the labels to match the rest of the figures:
‘Hnt’ to ‘Crystal cells’
‘ToPro’ to Nuclei’
‘domeMESO-GFP’ to ‘Progenitors’*

We have corrected the labels in the revised figure.

*3. Line 138-139 should use the flybase accepted term for dilps (Ilp2 and Ilp6) similar to what is used in Figure 2 or both terms as is used in the discussion (lines 478-480)*

We have corrected the erroneous nomenclature.

*4. Typos/misspellings: line 36, 168, 209,*

Thanks for catching these errors.  *5. Figure call on line 325 may be incorrect*

We have corrected the figure cell out  *6. Citations are missing after the statement “The PSC has an established role in orchestrating the behaviour of different cell types in the lymph gland” on line 353. These citations are important because the types of differences the PSC has been shown to orchestrate would have an impact on what would be expected from manipulations of sex in the PSC.*

We agree with the reviewer and have added citations. Specifically we now cite:

Hao Y, Jin LH. Dual role for Jumu in the control of hematopoietic progenitors in the *Drosophila* lymph gland. *eLife*. 2017;6:e25094. doi:10.7554/eLife.25094.

Pennetier D, Oyallon J, Morin‑Poulard I, Dejean S, Vincent A, Crozatier M. Size

control of the Drosophila hematopoietic niche by bone morphogenetic protein

signaling reveals parallels with mammals. Proc Natl Acad Sci U S A.

2012;109(9):3389–94. doi:10.1073/pnas.1109407109

Krzemień J, Dubois L, Makki R, Meister M, Vincent A, Crozatier M. Control of blood

cell homeostasis in Drosophila larvae by the posterior signalling centre. Nature.

2007;446(7133):325–8. doi:10.1038/nature05650

Mandal L, Martinez‑Agosto JA, Evans CJ, Hartenstein V, Banerjee U. A Hedgehog‑

and Antennapedia‑dependent niche maintains Drosophila haematopoietic precursors. Nature. 2007;446(7133):320–4. doi:10.1038/nature05585

*7. In line 834, ‘insulin’ should be changed to ‘insulin pathway’, since the experiments involve activating the pathway rather than insulin itself.*

We have made the requested change.

**Response to reviewer #2:**

1. *Some of this fact of the PSC on cellularity is controlled by Insulin signaling, as shown with PSC-specific Insulin Receptor knockdown or activation, which effects crystal numbers significantly, with a sex:genotype interaction. While the overall cellularity trends in the same direction, the effect is not as robust and lacks a sex:genotype interaction.This finding is reminiscent of the finding in Figure 1, where only the crystal cell numbers were different after correct for body weight. Perhaps the authors should focus on this difference, which seems to represent a true difference in developmental outcomes rather than a difference only in size? At a minimum, some context and connections between this data and earlier data should be included.*

In line with the point highlighted by the reviewer we have now highlighted, in the revised manuscript the similarity between the findings in figure 1 and the role of insulin signalling in the PSC. This can be seen on page 24 line 559-563. We also want to emphasize that while we do believe the particularly pronounced sex specific differences in crystal cell numbers are of great interest, this does not diminish, at least in our eyes, the other differences that are more aligned with the general differences in size. The reason is that there still needs to be some mechanism acting in the LG that scales up its size in line with these more general differences in body size. Our finding for the involvement of the PSC in achieving these differences illustrates one such mechanism.

2. *In the last section of the manuscript, larvae are fed bacteria, either E. coli or Ecc15. It is not convincing that this E. coli feeding is an "infection", as claimed. The details on the E. coli strain and its virulence are not provided. Ecc15 feeding of larvae is an established infection methodology with clear evidences of both pathology and immune activation.*

Apologies for not providing more details. While it is true that the Ecc15 feeding approach is better documented and studied, the E. coli infection protocol we implemented is well established in our lab and has been characterised by us and used in a number of our previously published manuscripts (including the Ho et al. 2023 and Khadilkar et al. 2017 manuscript listed below ). Specifically, we used the sam ampicillin resistant, GFP expressing E. coli as was used in these papers. This has been clarified in the materials and methods on page 33 line 740.

*Ho, K. Y. L., Carr, R. L., Dvoskin, A. D., & Tanentzapf, G. (2023). Kinetics of blood cell differentiation during hematopoiesis revealed by quantitative long-term live imaging. eLife, 12, e84085.* [*https://doi.org/10.7554/eLife.84085*](https://doi.org/10.7554/eLife.84085)

*Khadilkar, R. J., Vogl, W., Goodwin, K., & Tanentzapf, G. (2017). Modulation of occluding junctions alters the hematopoietic niche to trigger immune activation. eLife, 6, e28081. https://doi.org/10.7554/eLife.28081*

3. *Ecc15 feeding showed a similar pattern, with crystal cells significantly increased only in females. The authors make a large, but confusing point, about how this trend did not reach significance in the sex:genotype interaction analysis, but do not provide even a speculation as to why this discrepancy compared to the robust statistical differences for females (***) in the Ecc feed females (only). This particular condition is particular noisy (Figure 10D); perhaps they lack of significance in the sex:genotype is more of issue with variation rather than a lack of actual interaction? The authors should provide more explanation on this disconnect in their statistical analysis and how it relates (or not) to (potential) real biological outcomes.*

We thank the Reviewer for making this point. We also wondered why the sex:treatment interaction was not significant for the Ecc-fed larvae with respect to crystal cells. To address this issue, we tested if a non-normal distribution within our data could explain why we did not see a sex:treatment interaction in crystal cell numbers in Ecc-fed larvae despite a clear trend toward a female-biased effect. We found our data were not normally-distributed and consequently used non-parametric tests to determine whether there was a sex:treatment interaction for the effect of Ecc infection on crystal cell number. Despite the fact that there is an apparent female-biased effect, our non-parametric test still did not detect a significant sex:treatment interaction. Specifically, in two types of non-parametric two way ANOVA, the Schreier-Ray-Hare test and an Aligned Rank Transform test, we obtained P values consistent with an insignificant interaction (p=0.1491 and p=0.599, respectively). This means that we cannot confidently conclude that female crystal cell numbers are increased to a greater degree than male crystal cell numbers due to Ecc infection. In our revised manuscript, we adjusted the results text, figure legend, and methods to reflect this additional statistical analysis. We also added text to acknowledge variability in female crystal cell numbers could potentially explain the lack of sex-biased effect, and state that future experiments will be needed to address this important question

4. *Minor comment:
   To this reader, the description of how the scRNAseq data was "sexed" seemed a bit off; certainly required careful reading of the Methods. I feel like the analysis was to score cells for the lncRNAs ROX1/2 to define sex, and the rest of the analysis flowed from there. I.e. there is nothing obvious in movie 1, left UMAP to show two groups within each cluster....until you also show the lncRNAs on the same UMAP, middle and right. I suspect this was a eureka moment, when this split was realized (once the sex specific RNAs were re-introduced into the analysis). Nonetheless, please make sure the narrative description of the analysis is complete; to this reader it was a bit confusing in that very first paragraph.*

Thank you for pointing this out. Upon re-reading this section, we agree that the explanation in the first paragraph was a bit confusing. Therefore, we rewrote the first paragraph to more clearly and completely explain up front how we used lncRNAs roX1 and roX2 to sex individual lymph gland cells in our analysis. We appreciate the suggestion and hope that this change adds clarity to the narrative.

**Response to reviewer #3:**

*Major comments:
1. Data on the specificity of the drivers used in this study are essential, as expression in other tissues has been previously reported (DOI: 10.1002/dvg.23600,* [*https://doi.org/10.7554/eLife.61409*](https://doi.org/10.7554/eLife.61409) *as an example). This is particularly relevant for Col-Gal4 which is expressed, as tep4-Gal4, in other tissues/cells including the posterior lobes of the lymph gland (*[*https://doi.org/10.7554/eLife.61409*](https://doi.org/10.7554/eLife.61409)*), thus impacting the interpretation of the lymph gland PSC as a mediator of sex specificity in the lymph gland.*

The reviewer identifies an important and difficult point, that is by no means unique to our study but impacts all studies on systemic signals that operate in the lymph gland. It is indeed likely that all the drivers we used, including Col-Gal4 and Dome-Meso are expressed in other tissues and in the posterior lobes. It is not really possible with available drivers to completely discount the contribution of other tissues with 100% certainty. This is an important caveat which we have now added as a section in the discussion on page 26 line 594-605. We attempted to address this by showing that wherever expression there is in other tissues the expression in the PSC for Col-Gal4 is high and specific, at least within the primary lobe of the lymph gland itself.

For testing the specificity of the collier-gal4 driver within the primary node of lymph gland, we stained for Antp (another PSC cell marker), in a collier-gal4 x UAS eGFP larvae, with collier cells marked in GFP. Colocalization between Antp+ cells and collier+ cells was assessed using contingency-table analysis and Fisher’s exact test, comparing observed double-positive cells to expected frequencies under independence. Total cell counts were pooled across various images in both males and females separately.

The results for females (n=1484 cells) showed a significant amount of colocalization between Antp+ cells and collier+ cells (p<0.0001). The results for males (n=974 cells) also showed a significant amount of colocalization between Antp+ and collier+ cells(p<0.0001).

*2. The authors do not document the efficiency of the genetic tools used in the experiments. The manuscript's main message is that females and males are not equivalent. Yet the authors do not show that the drivers used in their gain-of-function experiments are expressed at similar levels in both sexes and thus cannot exclude that the phenotypes may be at least in part due to different induction levels of UAS transgenes.*

This is an excellent suggestion. To test the efficiency of both the tep4-gal4 and collier-gal4 drivers in males and females, and ensure that these drivers are expressed at similar levels in both sexes, we expressed GFP in both of these drivers and, using imageJ, measured intensity across a variety of images with the same imaging settings (laser power, gain and offset). We did this by going through slices of images, finding full cells that were not overlapped with other cells, and selecting a 0.96^2^ area to measure intensity. Since collier-gal4 GFP is expressed in the nucleus, we always took intensity in the center of the cell. For tep4-gal4 GFP, which is expressed in the cell membrane, we took the intensity of a cell which was not touching another cell in order to not risk overlapping membranes and increased intensity.

In these results, we found that there was no significant difference between male and female mean intensity for collier-gal4 (p=0.0740), or for tep4-gal4 (p=0.5192). The graphs are shown below. This data is included in supplemental figure 2 and discussed in the text on pages 8-9 Lines 177-185.

*3. The authors use RNAi to knock down InR in the lymph gland. A single RNAi construct is used and no validation of the efficiency of the construct is presented.*

*This is a good question. We specifically chose a line that is available from the BDSC and was used extensively by other groups previously to suppress InR expression in Drosophila. The line’s effectiveness has been well established before and we list two examples below illustrating its efficiency:*

*In Raza et al, it was shown that knockdown of InR, as well as multiple other genes, resulted in “considerable migration delays” in egg chambers of Drosophila.*

*In Urwyler et al, it was shown that knockdown of InR, using the same line from bloomington (51518), resulted in a decrease in the number of dendrites in the Drosophila brain.*

*We now explain this about the lines in the paper text on page 19 lines 443-445*

*Urwyler, O., Izadifar, A., Vandenbogaerde, S., Sachse, S., Misbaer, A., & Schmucker, D. (2019). Branch-restricted localization of phosphatase Prl-1 specifies axonal synaptogenesis domains. Science (New York, N.Y.), 364(6439), eaau9952.* [*https://doi.org/10.1126/science.aau9952*](https://doi.org/10.1126/science.aau9952)

*Raza, Q., Choi, J. Y., Li, Y., O'Dowd, R. M., Watkins, S. C., Chikina, M., Hong, Y., Clark, N. L., & Kwiatkowski, A. V. (2019). Evolutionary rate covariation analysis of E-cadherin identifies Raskol as a regulator of cell adhesion and actin dynamics in Drosophila. PLoS genetics, 15(2), e1007720. https://doi.org/10.1371/journal.pgen.1007720*

*4. Given that two thirds of the adult hemocytes originate from the embryo rather than from the lymph gland, and that those hemocytes can provide systemic signals, could the authors check whether the embryonically derived hemocytes also show sexual dimorphism? If so, the authors should evoke/discuss the possibility that those cells may also affect the lymph gland sex specific phenotypes and account for different effects in the adult as well.*

*We spent a lot of time trying to address this question, but for various persistent technical reasons, our efforts have not been successful to date. We agree this is an important question in our future work and we plan on continuing to do our best to address this question. For now we have mentioned the possibility raised by the reviewer in the paper text in the discussion on page 25 line 589-591.*

*5. The manuscript describes differential expression of sex-determination genes following segregation of male and female lymph gland cells (lines 227–230). However, despite strong enrichment of lncRNA:roX1 and lncRNA:roX2, expression of msl-2 is low and restricted to a small fraction of male cells, while Sxl and tra show only marginal sex-based differences. Providing absolute expression levels of these genes, to be compared with other clusters’ genes/markers, would help interpretation. Also, tra and sxl are expressed in both sexes, then differentially spliced in females and males. Were the female specific isoforms taken into account in their analysis?*

To address the first part of your suggestion:

Thank you for the suggestion of including the absolute expression levels for *lncRNA:roX1,* *lncRNA:roX2, msl-2, Sxl,*and *tra.* We made a new table containing the total counts observed for these genes in male and female cells (Supplemental Table 1). Your observation is correct that in our data *msl-2, Sxl,* and *tra* are expressed at much lower levels than *lncRNA:roX1*or *lncRNA:roX2,* with *msl-2* exhibiting the lowest expression compared to the other genes*.* It is worth noting that the differences we observed in the absolute expression levels of these genes in our RNA-Seq data is in line with what was previously reported in the FlyCell Atlas 2 database (Krause et al. 2021). FlyCell Atlas 2 shows two orders of magnitude higher FPKM (Fragments Per Kilobase of transcript per Million reads) for *lncRNA:roX1* compared to *msl-2*. Given the significantly lower expression of *msl-2*, it is more likely to see “dropout” (e.g. cells showing no expression) in *msl-2* in single cell RNA-Seq data. This dropout is due to single cell RNA-Seq methods only capturing 10-15% of transcripts in any given cell, and thus making it less likely to capture lowly expressed genes (AlJanahi et al. 2018). Moreover, the sex-based differences we observe for *Sxl* (1.79x higher in female cells) and *tra* (1.26x higher in female cells) are similar to the sex differences reported for these genes in FlyCell Atlas 2, in which *Sxl* exhibits 1.81x higher expression in females and *tra* shows a 1.49x higher expression in females.

References:

Krause, S. A., Overend, G., Dow, J. A. T., & Leader, D. P. (2022). FlyAtlas 2 in 2022: Enhancements to the Drosophila melanogaster expression atlas. *Nucleic Acids Research*, *50*(D1), D1010–D1015. <https://doi.org/10.1093/nar/gkab971>

AlJanahi, A. A., Danielsen, M., & Dunbar, C. E. (2018). An introduction to the analysis of single-cell RNA-sequencing data. *Molecular Therapy Methods & Clinical Development*, *10*, 189-196. <https://doi.org/10.1016/j.omtm.2018.07.003>

To address the second part of your question:

Unfortunately, we are unable to take female- or male-specific isoforms into account in our analysis. This is not possible due to the limitations of the sequencing depth (we sequenced 21,200 cells with <32,500 reads per cell) and the technique used to make the single cell cDNA libraries (10X Single Cell 3’ GEX version 3) which provides “short reads from fragments of the 3’ end” of transcripts (Arzulluz-Luque, 2018) This is a limitation of many single cell RNA-Seq studies due in large part to the high costs required to achieve the necessary sequencing depth for isoform analysis for tens of thousands of cells (e.g. the >20000 cells analyzed in this study). There have been some studies that performed isoform analysis on single cell RNA-Seq data, however, these used specialized single cell cDNA library prep techniques that provide reads across the full length of transcripts and these studies sequenced <100 cells to achieve the necessary sequencing depth (20-40 million reads per cell) required for isoform analysis (Arzulluz-Luque, 2018). Newer technologies such as PacBio circular consensus sequencing also enable isoform analysis in single cell RNA-Seq.

References:

Arzalluz-Luque, Á., & Conesa, A. (2018). Single-cell RNAseq for the study of isoforms—How is that possible? Genome Biology, 19(1), 110. <https://doi.org/10.1186/s13059-018-1496-z>

Shi, Z.-X., Chen, Z.-C., Zhong, J.-Y., Hu, K.-H., Zheng, Y.-F., Chen, Y., Xie, S.-Q., Bo, X.-C., Luo, F., Tang, C., Xiao, C.-L., & Liu, Y.-Z. (2023). High-throughput and high-accuracy single-cell RNA isoform analysis using PacBio circular consensus sequencing. Nature Communications, 14(1), 2631. <https://doi.org/10.1038/s41467-023-38324-9>

We appreciate your comment and hope that as technology advances, isoform analysis can be applied more frequently to address questions similar to the one you posed.

*Furthermore, the sex-biased expression of TkR99D, rdgA, Ilp6, and MCTS1 in the PSC raises interesting questions. Do these genes have known roles in crystal cell biology? Are their expression levels affected in col>traF or col>InR RNAi contexts?*

*We agree that these are important questions that merit comprehensive analysis and this will form a major part of our future analysis.*

*6. Fig 3 panels E and F show that crystal cells show no sex difference in the driver control. Given this data, it is hard to conclude that neuronal derived feminizing signals do not act on the lymph gland. Perhaps using a different neuronal driver will help circumventing this issue and eliminating confounding results.*

We appreciate the reviewer’s concern here. The line of experiment we are undertaking is challenging as it involves complex genotypes and the phenotypes we are assaying are sensitive to genetic background. Because of this we sought to be as rigorous as possible in how we interpreted the results and set a high threshold to accept an effect as real. In particular the thresholds that needed to be met to deem a result as meaningful was observing BOTH the multiple comparisons across genotypes showing a significant p value, as well as the sex:genotype interaction being significant. When it came to the feminization of the CNS, these standards were not fully met.

We again spent a great deal of time trying different drivers and did not obtain more clear cut results. For example, using another common neuronal driver, C2-gal4, did not give more conclusive results. We understand the reviewer’s concern and have softened our language in how we present our results in regards to neuronal feminization. We now specifically state that with the tools currently available we were unable to find definitive evidence for neuronal involvement for neuronally derived signals in sex-differences in the lymph gland. See page 14 line 335-337.

*7. The authors utilize the overexpression of the traF gene in CNS, fat body, lymph gland progenitors and PSC to “feminize” these tissues. To support this strategy, authors may want to elaborate on the cellular/molecular impact of traF overexpression on these tissues. For instance, are there differences in size? Do they show altered expression of sex-related or sex-specific genes?*

We failed to set this experiment in context. In the revised manuscript we have elaborated on this topic in line with reviewer suggestions on page 13 Lines 304-311.

The experiment of using TraF to feminize various tissues is a commonly used technique in the field. Some examples include the feminization of the *Drosophila* CNS, fat body, and overall fly(Rideout, Hudry, DeFalco, Argue). Overall, using these techniques leads to differences in physiology as well as changes in tissue size and gene expression.

Physiology: In Rideout et al. 2015, TraF was expressed using the *daughterless (da)*- gal4 driver, and showed that male pupae expressing TraF were significantly bigger than their wild type male counterparts, and were comparable to wild type female pupae(Figure 1). Also, that over-expression of TraF in females also showed an increase in body size even compared to their wild type female counterparts(Figure 1).

In Hudry et al. 2016, the use of UAS TraF in intestinal progenitors (using *esg-*gal4) showed an increase in regenerative proliferation of intestinal stem cells post tissue damage in males compared to their control counterparts, comparable to control females(Figure 2).

Molecular data as to ability of TraF to control sex identity comes from: It was also shown that the expression of UAS TraF in intestinal progenitors using *esg*-gal4, in an otherwise *tra* null female, was sufficient for the female specific isoform expression of the *tra* gene (Figure 3).

In DeFalco et al. 2008, UAS TraF was ubiquitously expressed in the soma (using *tubulin*-gal4) in *Drosophila* embryos, and found that both XX and XY embryonic gonads lacked pigment cells, which are a precursor of the Dr*osophila* testis, while ovaries do not have pigment cells(Figure 4).

Physiology: In Argue et al. 2014, it was shown that expression of TraF using Cg-gal4 (expressed in the fat body) had an effect on feminized males that made them physiologically and act like females. For example, it was found that feminized fat bodies in males reversed heart rate to that of the opposite sex following starvation stress(Figure 2).

Rideout EJ, Narsaiya MS, Grewal SS. The sex determination gene transformer

regulates male‑female differences in Drosophila body size. PLoS Genet.

2015;11(12):e1005683. doi:10.1371/journal.pgen.1005683

Hudry B, Khadayate S, Miguel‑Aliaga I. The sexual identity of adult intestinal stem

cells controls organ size and plasticity. Nature. 2016;530(7590):344–8.

doi:10.1038/nature16953

DeFalco, T., Camara, N., Le Bras, S., & Van Doren, M. (2008). Nonautonomous sex	determination controls sexually dimorphic development of the Drosophila gonad.	*Developmental cell*, *14*(2), 275–286. <https://doi.org/10.1016/j.devcel.2007.12.005>

Argue KJ, Neckameyer WS. Altering the sex determination pathway in Drosophila

fat body modifies sex‑specific stress responses. Am J Physiol Regul Integr

Comp Physiol. 2014;307(1):R82–92. doi:10.1152/ajpregu.00003.2014

*8. Along this line, since male PSC driven feminization via traF overexpression leads to a crystal cell number phenotype similar to that obtained upon inactivation of the Insulin Receptor (InR) in males, could the authors assess the levels of InR expression in the traF overexpressing male PSC? This will corroborate the statement made at line 414: ‘the insulin pathway in the PSC mediates sex differences in crystal cell number’ an in the discussion at line 456.*

*Yet again we spent a great deal of time trying various approaches to address this question but were not successful. We therefore softened our conclusions and discussed the important caveat raised by the reviewer on page 21 lines 483-484.*

*9. How do the authors account for effects coming from different genetic backgrounds, which may confound the interpretation of some data? For example, Fig 3 panels A and B show differences in the nuclear count between control females and males. In other controls, there is no such difference, see Fig 5 panels A,B (driver control)*

We agree with the reviewer that the genetic backgrounds we use in our manuscript sometimes differ in the magnitude of the sex difference in traits related to organ size or cell number. This was not unexpected, as genetic background has an effect on the sex-bias in complex traits such as size. Reassuringly, on occasions where the sex difference is not significant, we still detected a strongly sex-biased trend that agrees with the overall sex-bias we report (e.g., Figure 5A,B there is a strong trend toward a female bias in cell number that is simply not significant). We therefore do not believe that this issue confounds our main interpretation and conclusions. However, to ensure readers are aware of this issue, we added text to the revised manuscript for transparency (page 16 line 386-393):

*“As before, we compared experimental group flies to flies heterozygous for either the Tra^F^ construct (Tra^F^/+) or the Gal4 driver (tep4-Gal4/+) and performed statistical analysis by determining the sex:genotype interaction constant [MOU1] (Table 1). While we note that the magnitude of sex differences in lymph gland parameters in the Tep4-GAL4 control was not as large as in other strains, this was not unexpected given known effects of genetic background on sex difference in size-related traits (cite Shingleton here). Because we still observed a strong female bias in these parameters in Tep4-GAL4, we performed our analysis as with other strains.”*

*10. The authors state (lines 269-271) that to control for differences in the genetic backgrounds, they use two controls (driver alone, UAS transgene alone in heterozygous conditions). Yet, they introduce differences in the genetic background with domeMesoGFP. The Gal4 controls are obtained by crossing Gal4 lines with domeMesoGFP (final genotype Gal4/+; domeMesoGFP/+) and the UAS control by crossing UAS-traF; domeMesoGFP with domeMesoGFP (final genotype UAS-traF/+; domeMesoGFP). Could homozygous domeMesoGFP explain the differences seen in Fig. 3E (males), 3I (females), 4E (males), 4I (females)...? Control by crossing with domeMesoGFP is also used in InR KD/GOF experiments, which produces controls with different genetic backgrounds according to the methods section.*

We agree with the reviewer that this was an important missing control which we have now added (see Supplemental figure 5, included below) to page 29 lines 665-667. The data shows the presence of DomeMesoGFP has no effect on sex differences in the lymph gland.

***Heterozygous/Homozygous domeMESOGFP*** *Heterozygous cross of UAS TraF;+ x domeMESOGFP and homozygous cross of UAS TraF;domeMESOGFP x domeMESOGFP, separated by sex. (A) Raw total nuclei count of heterozygous females(n=17) and homozygous females(n=16) are shown to have no significant difference (p=0.2208). (B) Raw total nuclei count of heterozygous males(n=21) and homozygous males (n=16) are shown to have no significant difference (p=0.2259). (C) Significant difference between heterozygous females and males (p=0.0007), and significant difference between homozygous females and males (p=0.0067). (D) Raw crystal cell count of heterozygous females (n=17) and homozygous females (n=16) are shown to have no significant difference (p=0.3339). (E) Raw crystal cell count of heterozygous males (n=21) and homozygous males (n=16) are shown to have no significant difference (p=0.6017). (F) Significant difference between heterozygous females and males (p=0.0463), and significant difference between homozygous females and males (p<0.0001). (G) Raw domeMESO+ progenitor count of heterozygous females (n=17) and homozygous females (n=16) are shown to have no significant difference (p=0.7122). (H) Raw domeMESO+ progenitor count of heterozygous males (n=19) and homozygous males (n=16) are shown to have no significant difference (p=0.9136). (I) Significant difference between heterozygous females and males (p=0.0022), and significant difference between homozygous females and males (p=0.0025).*

*11. The 3D UMAP presented in Movie 1 shows a clear distinction between female and male clusters of hemocytes. The biggest sex-specific distance seems to be observed for the IZ cluster. Why was it disregarded in the analysis?*

*Thank you for pointing this out. We agree with you that the paper would be stronger with the analysis of all clusters, especially the IZ since it shows the clearest physical separation between male and female cells on the UMAP (Movie 1). We have addressed your concern by including analysis of the sex differences in the other clusters (including proPL, IZ, and PL) to Supplementary file 1 and updated Figure 2B to include data from the additional clusters. This additional analysis is described in the results section (page 11 lines 257-286). As a result of this analysis, we have identified additional differentially expressed genes (DEGs) that show sex differences (Supplementary file 1 – Table 1), performed gene set enrichment for the DEGs in these other clusters (Supplementary file 1-Table 2), highlighted some of the interesting DEGs found in these other clusters (Figure 2B; Supplementary file 1 – Table 3), and mapped which DEGs overlap between the clusters (Supplementary file 1-Table 4). For example, one finding that resulted from this additional analysis is that male IZ cells are enriched for many DNA replication genes and gene encoding protein folding chaperones (Supplementary file 1-Table 2), but these are not enriched in the female IZ cells or other sex-specific clusters. Some of the differentially expressed genes in the IZ cells from one sex are also enriched in other cell types from that same sex (e.g. CecB is enriched in female MZ, IZ, and PL cells, Figure 2B). Overall, we believe that including this additional analysis strengthens the paper. Thank you again for this suggestion.*

*12. Line 438-439: ‘the magnitude or even the existence of sex-differences in the response to infection may vary depending on the bacterial strain used’. How did the authors control for the absolute and sex specific food intake for the two infection protocols?*

This is an excellent point that we should have mentioned. Previous work by Sawala et al (reference below, with relevant section quoted) showed that in L2 and L3 larva, there are sex differences in food intake but that these are proportional to body size. In our context this means the same amount of infective material consumed would be similar once normalised for size. We now mention this on page 21 line 495-497.

*“We focused on measuring larval food intake at the early L2 and early L3 stages, when sex differences in growth rate are maximal. We found that the absolute food intake was higher for females than males (*[*S2 Fig*](https://pmc.ncbi.nlm.nih.gov/articles/PMC5627897/#pbio.2002252.s002)*). If higher food intake in females drives their increased growth rate, then females would be predicted to have a higher food intake than males per unit of body mass. However, we were unable to detect any significant male-female difference in food intake per unit of body mass (mass-specific food intake) at either early L2 or early L3 (*[*S2 Fig*](https://pmc.ncbi.nlm.nih.gov/articles/PMC5627897/#pbio.2002252.s002)*). We cannot rule out that very small differences in mass-specific food intake do exist between males and females but are hard to detect. Nevertheless, these results suggest that sex differences in absolute food intake are a consequence of preexisting body size differences rather than the driving force that establishes SSD.” from* Sawala, A., & Gould, A. P. (2017). The sex of specific neurons controls female body growth in Drosophila. *PLoS biology*, *15*(10), e2002252. https://doi.org/10.1371/journal.pbio.2002252

*13. The observed higher sensitivity of the female lymph gland to E. coli raises the question of whether this contributes to the reported lower survival of adult females upon infection (doi: 10.4161/fly.5082). Is this phenotype dependent on crystal cells, or could other lymph gland-derived or circulating immune cells be involved?

It is not obvious to us how the tendency to produce higher levels of crystal cells following exposure to E. Coli would reduce survival in females. We agree that delineating the role of specific populations of hemocytes in sex-differences in the response to infection should be a key goal of our future work.*

*Minor comments:

- Lines 460-461: The authors mention sex differences in the cellular immune response elicited by bacterial infection, referring to the counts of lymph gland hemocytes. In the larva, if the lymph gland is not ruptured, the cellular immune response is provided exclusively by the circulating hemocytes.*

Sorry for being unclear, we wanted to highlight similarities between our larval data on sex differences in the production of hemocytes and previous published reports of sex-differences in adults following infection. We did wish to confuse these two. We clarified this in the text. *- The scRNAseq data indicate a significant enrichment of NimC1 in female lymph glands compared to males (Table S1), which does not seem to coincide with the NimC1 labelling and count presented in Figure 1E, E’,J. Can the authors comment on this discrepancy?*

*See point 5 in the reply to reviewer #1.*
