# Supplementary material for: Sex differences in the regulation and function of cellular immunity in Drosophila
Source: PLoS Genet. 2026 Jul 10;22(7):e1012151. doi: 10.1371/journal.pgen.1012151 (PMC13399539; doi:10.1371/journal.pgen.1012151)
Supplement: Attachment — Submitted filename: Response to Reviewers2.docx [file pgen.1012151.s031.docx]

Dear Pablo,

Thanks again for sending our paper to the reviewers again. We are happy to see we have now satisfied 2 out of the 3 reviewers and that the third reviewer is mostly satisfied.

*Point by point reply to reviewer #3 comments:*

*1)Point 1: The authors point to the PSC as a mediator of sex specificity in the lymph gland, making it essential to address concerns regarding the specificity of the drivers used, particularly col-Gal4, which is expressed not only in the primary lobe’s PSC but also in other lymph gland lobes at late larval stage. To address this, the authors assessed colocalization between col-Gal4 and Antp in the primary lobe. However, this does not resolve the issue of potential unspecific effects. While we acknowledge the challenge of specific tools and appreciate the expanded discussion, the authors could for instance compare col expression across different sites to better estimate the possible contribution of these tissues. Earlier developmental stages may be also examined, when expression from posterior lobes should have less pronounced impact.*

While we agree that our experiments in this manuscript do not directly address which other tissues outside of the lymph gland express collier-Gal4, these collier-Gal4 expression profile experiments have been previously published in Benmimoun et al. PNAS 2015 (see Figure S1 from Benmimoun et al. below). From the figure, collier-GAL4 is also expressed in the brain across development and at the A/P boundary in the wing disc pouch in 3^rd^ instar larvae. Moreover, Benmimoun et al. report collier-Gal is expressed “sporadically in the posterior lobes”. However, given that our results with elav-Gal4 failed to find a role for the CNS in sex-specific effects on hematpoesis, we think it is very unlikely that collier-Gal4 expression in the brain is responsible for any of the lymph gland phenotypes observed. Similarly, given that the wing disc has never been implicated in hematopoiesis in the lymph gland and the posterior lobe expression in the lymph gland is sporadic, it is unlikely that these tissues are contributing the phenotypes observed in this manuscript. Importantly, although Benmimoun et al. demonstrated a role for Collier protein in lymph gland primary lobe core progenitors, the collier-Gal4 construct does not show expression in primary lobe core progenitors. Therefore, we think it is unlikely that the lymph gland primary lobe core progenitors are contributing to the observed phenotypes driven by collier-Gal4.

Based on the published data and the arguments presented above, we made the following changes to address the reviewer’s point—

We have added a short description of the published collier-Gal4 expression pattern outside of the lymph gland and a citation Benmimoun et al. paper to the discussion section in our revised manuscript (page 27, Lines 638-641).

*2) Point 3: To address the lack of validation for the experiments performed using a sole RNAi line targeting InR, the authors cite two studies in which this RNAi line was used. However, none of these studies directly test the ability of such RNAi to decrease InR expression and only in Urwyler et al alternative strategies were used to confirm the results obtained with this RNAi line. Authors may want to cite this work only to sustain the claim that “their effectiveness at modifying InR activity is well established [80-81].”*

As requested, we now cite only Urwyler et al.

We want to emphasize that the tool we are using is established tool used in many publications without the additional validation being requested here. We cite the BDSC number for this line (BDSC_51518) which allows a quick search that can reveal many other publications that use this reagent for in the same way we did, to knockdown InR expression.

For example

1) <https://www.biorxiv.org/content/10.64898/2026.01.29.702574v1.full>

2. <https://www.sciencedirect.com/science/article/pii/S2589004223015675>

3. <https://elifesciences.org/articles/70810/figures>

4. <https://www.cell.com/current-biology/fulltext/S0960-9822(18)30093-9>

5. <https://pmc.ncbi.nlm.nih.gov/articles/PMC9844989/>

6. <https://www.nature.com/articles/s41598-026-45137-5_reference.pdf>

7. https://www.pnas.org/doi/pdf/10.1073/pnas.2426930122

*3) Points 6 and 7: The authors aim to test whether sex-specific traits in the lymph gland are controlled by CNS sex by feminizing the CNS through TraF overexpression using the elav-Gal4 driver. To support their approach, they now cite studies reporting TraF-induced feminization, yet none use elav-Gal4, leaving unresolved whether this approach is sufficient to induce CNS feminization and calling for experimental validations. Feminization of cells expressing the elav driver should be validated by qPCR assays on known sex specific genes. Could the authors clarify what they mean by “we failed to set this experiment in context” or provide more fitting reference, if available?*

We again want to emphasize here that the approach we used, TraF overexpression using the elav-Gal4 driver, is widespread and established. This is not simply a matter of us “citing a few studies” bur rather us utilising a methodology that is both longstanding and extensively characterised for which there are many examples in the literature. It is true the more recently the approach of using *elav-GAL4* to drive UAS-tra has become less common in the field of sex differences. The reason is that people avoid using it in instances where behavior is a confounding factor as it was discovered that *tra*'s regulates courtship behavior. Improved tools, mainly the availability of many new drivers that allow targeting of specific subsets of neurons in the CNS have driven a shift away from pan-neuronal expression, though pan-neuronal expression is more suitable to our needs. However, here are some key papers that establish and mechanistically support the approach of feminizing the CNS using expression driven by *elav-GAL4*. Below are some examples, this is by no means a complete list:

<https://pubmed.ncbi.nlm.nih.gov/9418892/>

This paper shows that *tra* regulates *fru* splicing.

<https://www.sciencedirect.com/science/article/pii/S0092867405004071#bib28>

This paper shows that without the *tra* splice site in *fru,* females perform male-typical sexual behaviours.

<https://pmc.ncbi.nlm.nih.gov/articles/PMC2148331/#sec1>

This paper uses *elav-GAL4* to show that you can make a male have aggression patterns like you'd normally see in a female by *tra* expression. Note that this study did however observe some residual FruM in the CNS with elav-GAL4>UAS-tra, which may be due to the fact we now know *elav-GAL4* isn't completely pan-neuronal in adults (<https://academic.oup.com/genetics/article/229/3/iyaf014/8015406>). Nevertheless, given that FruM is necessary and sufficient for aggression (<https://pubmed.ncbi.nlm.nih.gov/17115036/>), the only logical conclusion is that it's via *tra* regulation of FruM even if FruM isn't completely absent.

<https://onlinelibrary.wiley.com/doi/epdf/10.1002/neu.10100?saml_referrer>

This paper uses *elav-GAL4* to show that *tra* expression in males abrogates courtship.

<https://www.nature.com/articles/nature04229>

This reference shows that loss of *tra* in neuron clones produces ectopic FruM expression in females.

<https://www.science.org/doi/10.1126/science.7846534?__cf_chl_tk=Q2ziiXqEB3brGxy9h70K8roKxcEHfldUKu_9s3PU_xA-1772120544-1.0.1.1-uodgkiIzwfyVukDdbMbSPipRsG68eVaharb1xWl3.X8>

This article was the first to show feminization using *tra* of various brain structures (not using elav) causes abnormal courtship and mating behaviour.

<https://www.jneurosci.org/content/18/11/4335>

a similar story but using the neuron specific driver *voila-GAL4* to feminizes mushroom bodies and some of antennal lobe.

*4) Moreover, the elav-Gal4 driver show genetic background issues, as control males do not display significantly reduced CC and progenitor numbers in the lymph gland. Although one alternative driver was tested, additional drivers for elav or other pan-neuronal markers could have been explored. In this context, the data mentioned in the rebuttal letter using the alternative C2-Gal4 driver should have been shown and a reference for that driver as well.*

We now include the C2-Gal4 driver data in the manuscript (supplemental figure 7). We also edited the text to be more cautious and clearly state that we failed to find evidence for involvement of the CNS in sex differences rather than that we completely discount this possibility (for example see pages 14 Lines 308-316).

Again, we want to emphasize that Elav-Gal4 is the gold standard for pan neuronal expression in that its expression has been studied extensively showing that it drives uniform, strong and specific expression in the CNS. The question is, given the best tools currently available can we find evidence for a role for the CNS in mediating sex differences in the lymph gland, the answer to that question is no.

*5) The title of the paragraph ‘We find no evidence that Sex differences in the lymph gland are mediated by the CNS’ does not fit with the statement “we were unable to find conclusive evidence in data and subsequent statistical analysis for the interpretation that sex differences in the lymph gland were mediated by the CNS” (lines 315–317). Either the limitations of their approach are more clearly explicated to place the conclusion in a more rigorous context or the data should be removed.*

We apologise that that the explanation of the results was unclear leader to some confusion. We have modified our conclusion to be more in line with the title of the paragraph. Specifically, for the data to be significant, the experimental flies must be different from both the UAS and the GAL4 control, and the direction of the effect must be the same. If those conditions are not met, then feminizing the CNS does not have an effect on lymph gland. Our language here was imprecise, we stated that we were 'unable to find conclusive evidence.....' but more precise way to describe our findings is to state the effects we saw were 'not statistically significant'. We have modified the results section accordingly on pages 14 Lines 308-316.

*6) Point 8: As the authors could not assess InR expression levels upon TraF overexpression in the PSC (using approaches that they do not specify in their response), it would be appropriate to explicitly acknowledge the possibility that pathways other than InR may contribute to sex differences in crystal cell number. Moreover, a comparison of the fold-change in crystal cell numbers upon TraF overexpression versus InR knockdown in the PSC could already provide a base for some speculations on this matter.*

As requested, we added to the text the possibility that pathways other than InR may contribute to sex differences in crystal cell number. We also mention, as the reviewer suggests that there is a very similar fold-change in crystal cell number (~1.75) upon either TraF overexpression or in InR knockdown in the PSC which argues that the InR pathway is a major contributor to the sex differences. This is found on page 21 lines 490-496; page 23 lines 559-566).

*7) Point 11: Authors may wish to comment on how the DEGs and pathways identified in their RNA-seq analysis—now expanded to additional lymph gland clusters—relate to the differential responses of male and female lymph gland populations to bacterial infection or the insulin pathway.*

Thank you for this helpful suggestion. We have added some comments about this in the discussion section (Page 25, Line 593-602). We think this addition to the text has improved the manuscript.

*8) I am not sure the authors address Point 9 (see our revision in 2025): While the increase in crystal cell number upon col>traF or col>InR RNAi is clear, this is unlikely to explain the observed increase in total nuclei in the primary lobe. Given that the number of dome+ progenitors remains unchanged, investigating the impact on other hemocyte subtypes (e.g., differentiated plasmatocytes) would help explaining how modulating sex-determination impact the lymph gland.*

We apologise for this oversight, during the preparation process of the rebuttal, where we addressed 25 separate (often multi part) reviewer queries, this point was somehow lost from the letter and overlooked. Our preliminary data suggests that this is more complicated than a simple case of an increase in plasmatocytes. We thus feel that this question is a major one and would like to tackle it with single cell sequencing, this will entail a substantial effort and constitute a key part of our future work on this topic. For the time being, we were very careful to make it explicit in the manuscript that first, the PSC only accounts for some and not all sex differences in the lymph gland, and second, that insulin signalling in the PSC only contributes to sex differences in crystal cells. This makes it clear that much work remains to uncover additional details of how sex differences in the lymph gland is established. (For example see page 18, lines 433-435 and page 21 lines 486-498)

*9) Point 11: Authors may wish to comment on how the DEGs and pathways identified in their RNA-seq analysis—now expanded to additional lymph gland clusters—relate to the differential responses of male and female lymph gland populations to bacterial infection or the insulin pathway.*

Thank you for this helpful suggestion. We have added some comments about this in the discussion section (Page 25, Line 593-602). We think this addition to the text has improved the manuscript.
