# Supplementary material for: Sex differences in the regulation and function of cellular immunity in Drosophila
Source: PLoS Genet. 2026 Jul 10;22(7):e1012151. doi: 10.1371/journal.pgen.1012151 (PMC13399539; doi:10.1371/journal.pgen.1012151)
Supplement: Peer review history

# Peer review: Decision Letter 0

**DOI:** 10.1371/journal.pgen.1012151.r001

**Authors:** Pablo Wappner

20 Aug 2025

PGENETICS-D-25-00772

Sex differences in the regulation and function of cellular immunity in *Drosophila*

PLOS Genetics

Dear Dr. Tanentzapf,

Thank you for submitting your manuscript to PLOS Genetics. After careful consideration, we feel that it has merit but does not fully meet PLOS Genetics's publication criteria as it currently stands. Therefore, we invite you to submit a revised version of the manuscript that addresses the points raised during the review process.

Please submit your revised manuscript within 60 days Oct 19 2025 11:59PM. If you will need more time than this to complete your revisions, please reply to this message or contact the journal office at plosgenetics@plos.org. When you're ready to submit your revision, log on to https://www.editorialmanager.com/pgenetics/ and select the 'Submissions Needing Revision' folder to locate your manuscript file.

Please include the following items when submitting your revised manuscript:

* A rebuttal letter that responds to each point raised by the editor and reviewer(s). You should upload this letter as a separate file labeled 'Response to Reviewers'. This file does not need to include responses to any formatting updates and technical items listed in the 'Journal Requirements' section below.

* A marked-up copy of your manuscript that highlights changes made to the original version. You should upload this as a separate file labeled 'Revised Manuscript with Track Changes'.

* An unmarked version of your revised paper without tracked changes. You should upload this as a separate file labeled 'Manuscript'.

If you would like to make changes to your financial disclosure, competing interests statement, or data availability statement, please make these updates within the submission form at the time of resubmission. Guidelines for resubmitting your figure files are available below the reviewer comments at the end of this letter.

We look forward to receiving your revised manuscript.

Kind regards,

Pablo Wappner

Section Editor

PLOS Genetics

Pablo Wappner

Section Editor

PLOS Genetics

Aimée Dudley

Editor-in-Chief

PLOS Genetics

Anne Goriely

Editor-in-Chief

PLOS Genetics

**Journal Requirements:**

1) Please ensure that the CRediT author contributions listed for every co-author are completed accurately and in full.

At this stage, the following Authors/Authors require contributions: Guy Tanentzapf. Please ensure that the full contributions of each author are acknowledged in the "Add/Edit/Remove Authors" section of our submission form.

The list of CRediT author contributions may be found here: https://journals.plos.org/plosgenetics/s/authorship#loc-author-contributions

2) Please upload all main figures as separate Figure files in .tif or .eps format. For more information about how to convert and format your figure files please see our guidelines:

https://journals.plos.org/plosgenetics/s/figures

3) Please ensure that the funders and grant numbers match between the Financial Disclosure field and the Funding Information tab in your submission form. Note that the funders must be provided in the same order in both places as well.

State what role the funders took in the study. If the funders had no role in your study, please state: "The funders had no role in study design, data collection and analysis, decision to publish, or preparation of the manuscript.".

If you did not receive any funding for this study, please simply state: u201cThe authors received no specific funding for this work.u201d

**Reviewers' comments:**

Reviewer's Responses to Questions

**Comments to the Authors:**

**Please note here if the review is uploaded as an attachment.**

Reviewer #1: The manuscript by Dvoskin et al. provides important and novel insights into how sex differences in immunity are established and how these sex differences influence the size, cellular composition, and bacterial infection response of the Drosophila hematopoietic organ. The manuscript is well written, presents a substantial amount of data (with appropriate sample size and statistical methods), and the findings are thorough and thought-provoking. This is an important piece of work that is appropriate for this journal. However, in my view, additional experiments are needed (or sections need to be rewritten) to support some of the authors’ conclusions fully.

Major comments:

1. The authors report raw PSC numbers that are significantly higher (68±22 for males and 91±24 for females) than those reported by other studies (typically 30-40 cells per lobe). Indeed, in lines 126-127, the authors define the PSC as “a group of a few dozen cells”. Why are their PSC numbers reported throughout the paper significantly higher? If this is simply a difference in analysis methods, this should be noted and explained in the text to give clarity to the reader and so as not to confuse the field.

2. The authors show that crystal cell numbers differ significantly between males and females even after normalizing to body weight. Would normalizing crystal cell numbers to the total lymph gland cell numbers still reveal a difference between sexes? Crystal cell numbers are known to be affected by the lymph gland size, and for this reason, most studies usually report crystal cell percentage rather than raw counts. This analysis could reveal whether the reported correlation between crystal cell number and lymph gland size is similar between the sexes or due to inherent differences in lymph gland size between the sexes.

3. Lines 163-167 say that the authors labeled progenitors with domeMESO GFP, but the figure and figure legend for Figure 1 indicate that the progenitors are labeled by Tep4gal4; UAS-GFP. This should be clarified and corrected in the text and/or figure legends.

4. In fact, throughout the manuscript, the authors use Tep4 and domeMESO drivers interchangeably. However, it is well established that Tep4 is expressed only in core progenitors, whereas domeMESO is expressed in both core and distal progenitors (PMID: 32243888). Several studies (including those from the authors: PMID: 34713801) have shown that genetic manipulations with these drivers can yield different results, especially as these populations are in different stages of the cell cycle and to differentially respond to signaling pathway manipulations (e.g., PMID: 38866012 ; PMID: 39466810). For this reason:

a. For Figure 1, I recommend assessing the number of domeMESO-positive progenitors (as the text mentions) to determine whether they differ significantly between males and females, similar to the analysis for Tep4 progenitors (presumably what was done in Figure 1).

b. The difference between the populations marked by Tep4 and domeMESO should be explained in the text, clearly stating that when using Tep4, these are core progenitors. In contrast, when using domeMESO, the authors can use the term 'progenitors'. And the text describing the results from these figures should be double-checked for accuracy to confirm the correct driver is referenced.

5. For scRNASeq analysis, why weren’t sex differences compared, or results shown for comparisons between proPL, IZ, and PL clusters? The authors noted (lines 200-202) that these clusters were particularly different between sexes in the UMAP, so I am curious why these were not compared. These comparisons should be included for completeness, and if no DEGs were identified, this should be reported by the authors.

a. Interestingly, NimC1 (the antigen for P1 antibody, which the authors use for Plasmatocyte staining and is a marker for PL) was identified as a top overall gene differentially expressed between sexes (in the supplemental table). The authors should consider including it in the bubble plot for Figure 2A.

6. I am not sure I agree with the author’s conclusions about the results from feminizing the progenitors in Figure 5. When comparing males and females in tep4gal4>TraF for nuclei count (Figure 5B), crystal cell count (Figure 5F) and progenitor count (Figure 5J), all of these are no longer significantly different between males and females and thus suggest that feminizing the females and males reverses the sex-bias in these numbers. Furthermore, the interaction constants for nuclei and progenitors are significant in the feminized progenitors experiments. This suggests that indeed some sex differences in the lymph gland are mediated by the progenitors. But I would agree that sex differences in crystal cell number in the lymph gland are not mediated by the Tep4+ progenitors.

a. This data may be difficult to interpret because there are smaller differences between males and females in the tep4-gal4 only controls, but this underscores the need to repeat these experiments with domeMESO-GAL4 to truly conclude that “Sex differences in the lymph gland are not mediated by progenitors” (line 330). Thus, in Figure 5, I suggest using the domeMESO-GAL4 driver to feminize progenitors in addition to Tep4, as this might yield different results

b. Note: the gal4 insertion in Tep4 (from Kyoto DGRC) may disrupt gene function (hence why it cannot go homozygous), and so the smaller differences between males and females in this background may be biologically relevant.

c. Similarly, in Figure 5, I recommend quantifying Tep4-positive core progenitor numbers, as these may show significant differences. Indeed, if that is what was used in Figure 1, there were significant differences in Tep4-positive core progenitors in wildtype and so quantifying those numbers in addition to the domemeso+ progenitors in the feminized backgrounds is essential for making conclusions about the role of progenitors in sex differences.

7. The authors state that “it should become a standard practice to consider sex as a key variable for all phenotypic characterization of the lymph gland.” But the authors also noted that controlling for body weight eliminates most raw count differences for nuclei, PSC, and progenitors (and that there were no differences for plasmatocytes). Most studies do not report raw counts but rather normalized counts to lymph gland size (raw count/total nuclei). Figure 1 should include sex-specific “normalized” values, as this would give important context to interpreting many past experiments.

a. Another important point the authors may want to point out is that earlier work using domeless-GAL4 to drive manipulations in the progenitors (or other GAL4s that are lethal in males) only looked at these manipulations in female animals, as it would be impossible to drive the GAL4 in males as it is lethal. So there could be an important and interesting missing body of data on the impact of some of the same signaling pathways mentioned in the introduction that is yet to be explored (and discrepancies between results from manipulations that use dome-GAL4 and domeMESO-GAL4, which is not lethal in males, could suggest sex-specific effects and would make it that much more essential to take sex into account when analyzing and interpreting results)

Minor Questions:

1. For the insulin pathway experiments, have the authors considered testing additional pathway members, such as expressing a constitutively active form of PI3K or knocking down the kinase Akt?

2. How do PSC feminized males respond to infection with P. carotovorum and E. coli?

Minor Comments:

1. In all figures except for the last two, the labels for nuclei, crystal cells, and progenitors should be in larger, brighter font to improve visibility.

2. In Figure 6, authors should update the labels to match the rest of the figures:

‘Hnt’ to ‘Crystal cells’

‘ToPro’ to Nuclei’

‘domeMESO-GFP’ to ‘Progenitors’

3. Line 138-139 should use the flybase accepted term for dilps (Ilp2 and Ilp6) similar to what is used in Figure 2 or both terms as is used in the discussion (lines 478-480)

4. Typos/misspellings: line 36, 168, 209,

5. Figure call on line 325 may be incorrect

6. Citations are missing after the statement “The PSC has an established role in orchestrating the behaviour of different cell types in the lymph gland” on line 353. These citations are important because the types of differences the PSC has been shown to orchestrate would have an impact on what would be expected from manipulations of sex in the PSC.

7. In line 834, ‘insulin’ should be changed to ‘insulin pathway’, since the experiments involve activating the pathway rather than insulin itself.

8. I appreciated the explanation for why the authors chose the statistical test used in Figure 3 (lines 273-279).

Reviewer #2: In this new manuscript, the authors present a careful and detailed analysis of sex differences in drosophila larval hematopoiesis, in the lymph gland. First they carefully show that overall cellularity and most cell types (but not mature plasmatocytes - Drosophila macrophages) are increased in raw number in females, relative to males. However, when corrected for body weight, only the crystal cell numbers were different between sexes. Reanalyzing previous scRNAseq data (from one of the authors), the team shows clear differences in gene expression across nearly all hematopoietic cell types, via scRNAseq; presumably some of these gene expression changes effect attributes beyond cell number. They then shift to back focusing on the sexual dimporphisms of numbers hemotapoetic cell types, with a carefully controlled analysis of genetically transforming sex, feminizing particular tissue with programmed expression of TraF, a very elegant experimental design made possible with the tools unique to the fly model.

Unfortunately, using these sex transforming tools, the sexually dimorphic gene expression not examined further, which seems like a missed opportunity.

Nonetheless, they do show that sex transformation of the PSC, the developmental/stem cell niche in the lymph gland (but not other cells in the gland nor brain nor fat body) can alter cellularity in otherwise male animals, to mimic the female numbers of total cells and crystal cells, but not others.

Some of this fact of the PSC on cellularity is controlled by Insulin signaling, as shown with PSC-specific Insulin Receptor knockdown or activation, which effects crystal numbers significantly, with a sex:genotype interaction. While the overall cellularity trends in the same direction, the effect is not as robust and lacks a sex:genotype interaction. This finding is reminiscent of the finding in Figure 1, where only the crystal cell numbers were different after correct for body weight. Perhaps the authors should focus on this difference, which seems to represent a true difference in developmental outcomes rather than a difference only in size? At a minimum, some context and connections between this data and earlier data should be included.

In the last section of the manuscript, larvae are feed bacteria, either E. coli or Ecc15. It is not convincing that this E. coli feeding is an "infection", as claimed. The details on the E. coli strain and its virulence are not provided. Ecc15 feeding of larvae is an established infection methodology with clear evidences of both pathology and immune activation. Nonetheless, E. coli feeding show a clear sexually dimorphic effect on crystal cell numbers (these same cells, again!...why always crystal cells? Anything from teh gene expression profile that might inform?),while other cell types maybe change in number, but in a similar pattern in both sexes. Ecc15 feeding showed a similar pattern, with crystal cells significantly increased only in females. The authors make a large, but confusing point, about how this trend did not reach significance in the sex:genotype interaction analysis, but do not provide even a speculation as to why this discrepancy compared to the robust statistical differences for females (***) in the Ecc feed females (only). This particular condition is particular noisy (Figure 10D); perhaps they lack of significance in teh sex:genotype is more of issue with variation rather than a lack of actual interaction? The authors should provide more explanation on this disconnect in their statistical analysis and how it relates (or not) to (potential) real biological outcomes.

Overall, this is a well executed, rigorous study, although much of the data (with the exception of the InR/PSC analysis) is observational, and as the authors write "identifies important areas for future exploration."

Minor comment:

To this reader, the description of how the scRNAseq data was "sexed" seemed a bit off; certainly required careful reading of the Methods. I feel like the analysis was to score cells for the lncRNAs ROX1/2 to define sex, and the rest of the analysis flowed from there. I.e. there is nothing obvious in movie 1, left UMAP to show two groups within each cluster....until you also show the lncRNAs on the same UMAP, middle and right. I suspect this was a eureka moment, when this split was realized (once the sex specific RNAs were re-introduced into the analysis). Nonetheless, please make sure the narrative description of the analysis is complete; to this reader it was a bit confusing in that very first paragraph.

Reviewer #3: Dvoskin et al. investigate the differences in the development of the larval lymph gland between female and male Drosophila. They report that female lymph glands are bigger than male lymph glands, which correlates with the higher body weight of females. The female lymph glands present an excess of crystal cell counts compared to males. They reanalyze lymph gland single-cell RNA sequencing data previously generated by Girard et al. to determine transcriptional differences between males and females for the subgroup of lymph gland hemocytes. Following this, they overexpress traF in neurons, fat body, hemocyte progenitors and posterior signaling center (PSC) to feminize the organs/tissues and determine their roles in the sex biases in lymph gland development. Their findings highlight the PSC, targeted via col-Gal4, and insulin signaling as modulators of lymph gland size (total cell number) and/or crystal cell abundance in males. Finally, they challenge the animals with E. coli and P. carotovorum and show that females respond more strongly to E. coli infection than males.

Overall data and statistics are robust and well-presented. So far, sex differences have not been taken systematically into account while it has become clear that they are important parameters to document and consider in most studies. Thus, the discoveries presented in the manuscript are novel and will be relevant for a broad audience.

Major comments:

1. Data on the specificity of the drivers used in this study are essential, as expression in other tissues has been previously reported (DOI: 10.1002/dvg.23600, https://doi.org/10.7554/eLife.61409 as an example). This is particularly relevant for Col-Gal4 which is expressed, as tep4-Gal4, in other tissues/cells including the posterior lobes of the lymph gland (https://doi.org/10.7554/eLife.61409), thus impacting the interpretation of the lymph gland PSC as a mediator of sex specificity in the lymph gland.

2. The authors do not document the efficiency of the genetic tools used in the experiments. The manuscript's main message is that females and males are not equivalent. Yet the authors do not show that the drivers used in their gain-of-function experiments are expressed at similar levels in both sexes and thus cannot exclude that the phenotypes may be at least in part due to different induction levels of UAS transgenes.

3. The authors use RNAi to knock down InR in the lymph gland. A single RNAi construct is used and no validation of the efficiency of the construct is presented.

4. Given that two thirds of the adult hemocytes originate from the embryo rather than from the lymph gland, and that those hemocytes can provide systemic signals, could the authors check whether the embryonically derived hemocytes also show sexual dimorphism? If so, the authors should evoke/discuss the possibility that those cells may also affect the lymph gland sex specific phenotypes and account for different effects in the adult as well.

5. The manuscript describes differential expression of sex-determination genes following segregation of male and female lymph gland cells (lines 227–230). However, despite strong enrichment of lncRNA:roX1 and lncRNA:roX2, expression of msl-2 is low and restricted to a small fraction of male cells, while Sxl and tra show only marginal sex-based differences. Providing absolute expression levels of these genes, to be compared with other clusters’ genes/markers, would help interpretation. Also, tra and sxl are expressed in both sexes, then differentially spliced in females and males. Were the female specific isoforms taken into account in their analysis?

Furthermore, the sex-biased expression of TkR99D, rdgA, Ilp6, and MCTS1 in the PSC raises interesting questions. Do these genes have known roles in crystal cell biology? Are their expression levels affected in col>traF or col>InR RNAi contexts?

6. Fig 3 panels E and F show that crystal cells show no sex difference in the driver control. Given this data, it is hard to conclude that neuronal derived feminizing signals do not act on the lymph gland. Perhaps using a different neuronal driver will help circumventing this issue and eliminating confounding results.

7. The authors utilize the overexpression of the traF gene in CNS, fat body, lymph gland progenitors and PSC to “feminize” these tissues. To support this strategy, authors may want to elaborate on the cellular/molecular impact of traF overexpression on these tissues. For instance, are there differences in size? Do they show altered expression of sex-related or sex-specific genes?

8. Along this line, since male PSC driven feminization via traF overexpression leads to a crystal cell number phenotype similar to that obtained upon inactivation of the Insulin Receptor (InR) in males, could the authors assess the levels of InR expression in the traF overexpressing male PSC? This will corroborate the statement made at line 414: ‘the insulin pathway in the PSC mediates sex differences in crystal cell number’ an in the discussion at line 456.

9. While the increase in crystal cell number upon col>traF or col>InR RNAi is clear, this is unlikely to explain the observed increase in total nuclei in the primary lobe. Given that the number of dome+ progenitors remain unchanged, investigating the impact on other hemocyte subtypes (e.g., differentiated plasmatocytes) would help explaining how modulating sex-determination impact the lymph gland.

10. How do the authors account for effects coming from different genetic backgrounds, which may confound the interpretation of some data? For example, Fig 3 panels A and B show differences in the nuclear count between control females and males. In other controls, there is no such difference, see Fig 5 panels A,B (driver control).

11. The authors state (lines 269-271) that to control for differences in the genetic backgrounds, they use two controls (driver alone, UAS transgene alone in heterozygous conditions). Yet, they introduce differences in the genetic background with domeMesoGFP. The Gal4 controls are obtained by crossing Gal4 lines with domeMesoGFP (final genotype Gal4/+; domeMesoGFP/+) and the UAS control by crossing UAS-traF; domeMesoGFP with domeMesoGFP (final genotype UAS-traF/+; domeMesoGFP). Could homozygous domeMesoGFP explain the differences seen in Fig. 3E (males), 3I (females), 4E (males), 4I (females)...? Control by crossing with domeMesoGFP is also used in InR KD/GOF experiments, which produces controls with different genetic backgrounds according to the methods section.

12. The 3D UMAP presented in Movie 1 shows a clear distinction between female and male clusters of hemocytes. The biggest sex-specific distance seems to be observed for the IZ cluster. Why was it disregarded in the analysis?

13. Line 438-439: ‘the magnitude or even the existence of sex-differences in the response to infection may vary depending on the bacterial strain used’. How did the authors control for the absolute and sex specific food intake for the two infection protocols?

14. The observed higher sensitivity of the female lymph gland to E. coli raises the question of whether this contributes to the reported lower survival of adult females upon infection (doi: 10.4161/fly.5082). Is this phenotype dependent on crystal cells, or could other lymph gland-derived or circulating immune cells be involved?

Minor comments:

- Among the identified sex-specific DEGs, authors mention orthologs of human genes involved in blood development and disease. To add weight to this observation, authors may want to provide, if available, additional data on sex-specific expression of the human orthologs and/or gender-bias in disease incidence.

- Line 209: A space is missing.

- Lines 460-461: The authors mention sex differences in the cellular immune response elicited by bacterial infection, referring to the counts of lymph gland hemocytes. In the larva, if the lymph gland is not ruptured, the cellular immune response is provided exclusively by the circulating hemocytes.

- Line 239-241: a molecular validation of some DEG genes would add value to the transcriptomic data (qPCR? In situ hybridization? Immunohistochemistry?)

- The scRNAseq data indicate a significant enrichment of NimC1 in female lymph glands compared to males (Table S1), which does not seem to coincide with the NimC1 labelling and count presented in Figure 1E, E’,J. Can the authors comment on this discrepancy?

- There are mistakes in referring to the supplementary files (line 247-254)

**********

**Have all data underlying the figures and results presented in the manuscript been provided?**

Large-scale datasets should be made available via a public repository as described in the PLOS Genetics data availability policy, and numerical data that underlies graphs or summary statistics should be provided in spreadsheet form as supporting information.

*Reviewer #1: Yes*

*Reviewer #2: Yes*

*Reviewer #3: Yes*

**********

PLOS authors have the option to publish the peer review history of their article (what does this mean?). If published, this will include your full peer review and any attached files.

If you choose “no”, your identity will remain anonymous but your review may still be made public.

**Do you want your identity to be public for this peer review?**  For information about this choice, including consent withdrawal, please see our Privacy Policy.

*Reviewer #1: No*

*Reviewer #2: No*

*Reviewer #3: No*

*[NOTE: If reviewer comments were submitted as an attachment file, they will be attached to this email and accessible via the submission site. Please log into your account, locate the manuscript record, and check for the action link "View Attachments". If this link does not appear, there are no attachment files.]*

**Figure resubmission:**

-->*While revising your submission, we strongly recommend that you use PLOS’s NAAS tool (https://ngplosjournals.pagemajik.ai/artanalysis) to test your figure files. NAAS can convert your figure files to the TIFF file type and meet basic requirements (such as print size, resolution), or provide you with a report on issues that do not meet our requirements and that NAAS cannot fix.*

*After uploading your figures to PLOS’s NAAS tool - https://ngplosjournals.pagemajik.ai/artanalysis, NAAS will process the files provided and display the results in the "Uploaded Files" section of the page as the processing is complete. If the uploaded figures meet our requirements (or NAAS is able to fix the files to meet our requirements), the figure will be marked as "fixed" above. If NAAS is unable to fix the files, a red "failed" label will appear above. When NAAS has confirmed that the figure files meet our requirements, please download the file via the download option, and include these NAAS processed figure files when submitting your revised manuscript.*

**Reproducibility:**

To enhance the reproducibility of your results, we recommend that authors of applicable studies deposit laboratory protocols in protocols.io, where a protocol can be assigned its own identifier (DOI) such that it can be cited independently in the future. Additionally, PLOS ONE offers an option to publish peer-reviewed clinical study protocols. Read more information on sharing protocols at https://plos.org/protocols?utm_medium=editorial-email&utm_source=authorletters&utm_campaign=protocols

# Author comment: Author response to Decision Letter 1

**DOI:** 10.1371/journal.pgen.1012151.r002

29 Jan 2026

Attachment Submitted filename: Response to Reviewers.docx

# Peer review: Decision Letter 1

**DOI:** 10.1371/journal.pgen.1012151.r003

**Authors:** Pablo Wappner, Pablo Wappner

23 Feb 2026

PGENETICS-D-25-00772R1

Sex differences in the regulation and function of cellular immunity in Drosophila

PLOS Genetics

Dear Dr. Tanentzapf,

Thank you for submitting your manuscript to PLOS Genetics. After careful consideration, we feel that it has merit but does not fully meet PLOS Genetics's publication criteria as it currently stands. Therefore, we invite you to submit a revised version of the manuscript that addresses the points raised during the review process. The revised manuscript was evaluated by two of the three reviewers that analyzed the original version. As you can see below, one of the reviewers was entirely satisfied with the revised manuscript, and recommended acceptance. The other reviewer finds that the manuscript has improved, but still expresses some concerns that need to be attended.

Please submit your revised manuscript by Apr 24 2026 11:59PM. If you will need more time than this to complete your revisions, please reply to this message or contact the journal office at plosgenetics@plos.org. When you're ready to submit your revision, log on to https://www.editorialmanager.com/pgenetics/ and select the 'Submissions Needing Revision' folder to locate your manuscript file.

Please include the following items when submitting your revised manuscript:

* A letter that responds to each point raised by the editor and reviewer(s). You should upload this letter as a separate file labeled 'Response to Reviewers'. This file does not need to include responses to any formatting updates and technical items listed in the 'Journal Requirements' section below.

* A marked-up copy of your manuscript that highlights changes made to the original version. You should upload this as a separate file labeled 'Revised Manuscript with Track Changes'.

* An unmarked version of your revised paper without tracked changes. You should upload this as a separate file labeled 'Manuscript'.

If you would like to make changes to your financial disclosure, competing interests statement, or data availability statement, please make these updates within the submission form at the time of resubmission. Guidelines for resubmitting your figure files are available below the reviewer comments at the end of this letter.

We look forward to receiving your revised manuscript.

Kind regards,

Pablo Wappner

Section Editor

PLOS Genetics

Aimée Dudley

Editor-in-Chief

PLOS Genetics

Anne Goriely

Editor-in-Chief

PLOS Genetics

**Journal Requirements:**

If the reviewer comments include a recommendation to cite specific previously published works, please review and evaluate these publications to determine whether they are relevant and should be cited. There is no requirement to cite these works unless the editor has indicated otherwise.

**Reviewers' comments:**

Reviewer's Responses to Questions

**Comments to the Authors:**

**Please note here if the review is uploaded as an attachment.**

*Reviewer #1: The authors have thoroughly addressed my major and minor comments, and the revisions have significantly strengthened the manuscript. I appreciate the care taken to clarify the analyses and improve the presentation, and I believe the paper is now much improved. I commend the authors for their thoughtful responses and substantial improvements to the manuscript.*

Reviewer #3: The authors have improved the manuscript by adding some experiments and revising the text. With regard to the previously raised points:

Point 1: The authors point to the PSC as a mediator of sex specificity in the lymph gland, making it essential to address concerns regarding the specificity of the drivers used, particularly col-Gal4, which is expressed not only in the primary lobe’s PSC but also in other lymph gland lobes at late larval stage. To address this, the authors assessed colocalization between col-Gal4 and Antp in the primary lobe. However, this does not resolve the issue of potential unspecific effects. While we acknowledge the challenge of specific tools and appreciate the expanded discussion, the authors could for instance compare col expression across different sites to better estimate the possible contribution of these tissues. Earlier developmental stages may be also examined, when expression from posterior lobes should have less pronounced impact.

Point 3: To address the lack of validation for the experiments performed using a sole RNAi line targeting InR, the authors cite two studies in which this RNAi line was used. However, none of these studies directly test the ability of such RNAi to decrease InR expression and only in Urwyler et al alternative strategies were used to confirm the results obtained with this RNAi line. Authors may want to cite this work only to sustain the claim that “their effectiveness at modifying InR activity is well established [80-81].”

Points 6 and 7: The authors aim to test whether sex-specific traits in the lymph gland are controlled by CNS sex by feminizing the CNS through TraF overexpression using the elav-Gal4 driver. To support their approach, they now cite studies reporting TraF-induced feminization, yet none use elav-Gal4, leaving unresolved whether this approach is sufficient to induce CNS feminization and calling for experimental validations. Feminization of cells expressing the elav driver should be validated by qPCR assays on known sex specific genes. Could the authors clarify what they mean by “we failed to set this experiment in context” or provide more fitting reference, if available?

Moreover, the elav-Gal4 driver show genetic background issues, as control males do not display significantly reduced CC and progenitor numbers in the lymph gland. Although one alternative driver was tested, additional drivers for elav or other pan-neuronal markers could have been explored. In this context, the data mentioned in the rebuttal letter using the alternative C2-Gal4 driver should have been shown and a reference for that driver as well.

The title of the paragraph ‘We find no evidence that Sex differences in the lymph gland are mediated by the CNS’ does not fit with the statement “we were unable to find conclusive evidence in data and subsequent statistical analysis for the interpretation that sex differences in the lymph gland were mediated by the CNS” (lines 315–317). Either the limitations of their approach are more clearly explicated to place the conclusion in a more rigorous context or the data should be removed.

Point 8: As the authors could not assess InR expression levels upon TraF overexpression in the PSC (using approaches that they do not specify in their response), it would be appropriate to explicitly acknowledge the possibility that pathways other than InR may contribute to sex differences in crystal cell number. Moreover, a comparison of the fold-change in crystal cell numbers upon TraF overexpression versus InR knockdown in the PSC could already provide a base for some speculations on this matter.

Point 11: Authors may wish to comment on how the DEGs and pathways identified in their RNA-seq analysis—now expanded to additional lymph gland clusters—relate to the differential responses of male and female lymph gland populations to bacterial infection or the insulin pathway.

I am not sure the authors address Point 9 (see our revision in 2025): While the increase in crystal cell number upon col>traF or col>InR RNAi is clear, this is unlikely to explain the observed increase in total nuclei in the primary lobe. Given that the number of dome+ progenitors remains unchanged, investigating the impact on other hemocyte subtypes (e.g., differentiated plasmatocytes) would help explaining how modulating sex-determination impact the lymph gland.

Minor points

- Line 590: “we” to be removed.

- It is unclear why data on collier-Gal4 are mentioned in line 179.

- Line 255: Figure 1B should be Figure 2B.

- Figure 9L’: plasmatocyte is misspelled

**********

**Have all data underlying the figures and results presented in the manuscript been provided?**

Large-scale datasets should be made available via a public repository as described in the PLOS Genetics  data availability policy, and numerical data that underlies graphs or summary statistics should be provided in spreadsheet form as supporting information.

*Reviewer #1: None*

*Reviewer #3: Yes*

**********

PLOS authors have the option to publish the peer review history of their article (what does this mean?). If published, this will include your full peer review and any attached files.

If you choose “no”, your identity will remain anonymous but your review may still be made public.

**Do you want your identity to be public for this peer review?**  For information about this choice, including consent withdrawal, please see our Privacy Policy.

*Reviewer #1: No*

*Reviewer #3: No*

***Figure resubmission:***

*While revising your submission, we strongly recommend that you use PLOS’s NAAS tool (https://ngplosjournals.pagemajik.ai/artanalysis) to test your figure files. NAAS can convert your figure files to the TIFF file type and meet basic requirements (such as print size, resolution), or provide you with a report on issues that do not meet our requirements and that NAAS cannot fix.*

*After uploading your figures to PLOS’s NAAS tool - https://ngplosjournals.pagemajik.ai/artanalysis, NAAS will process the files provided and display the results in the "Uploaded Files" section of the page as the processing is complete. If the uploaded figures meet our requirements (or NAAS is able to fix the files to meet our requirements), the figure will be marked as "fixed" above. If NAAS is unable to fix the files, a red "failed" label will appear above. When NAAS has confirmed that the figure files meet our requirements, please download the file via the download option, and include these NAAS processed figure files when submitting your revised manuscript.*

**Reproducibility:**

To enhance the reproducibility of your results, we recommend that authors of applicable studies deposit laboratory protocols in protocols.io, where a protocol can be assigned its own identifier (DOI) such that it can be cited independently in the future. Additionally, PLOS ONE offers an option to publish peer-reviewed clinical study protocols. Read more information on sharing protocols at https://plos.org/protocols?utm_medium=editorial-email&utm_source=authorletters&utm_campaign=protocols

# Author comment: Author response to Decision Letter 2

**DOI:** 10.1371/journal.pgen.1012151.r004

13 Apr 2026

Attachment Submitted filename: Response to Reviewers2.docx

# Editor report: Decision Letter 2

**DOI:** 10.1371/journal.pgen.1012151.r005

**Authors:** Pablo Wappner, Pablo Wappner, Pablo Wappner

28 Apr 2026

Dear Dr Tanentzapf,

We are pleased to inform you that your manuscript entitled "Sex differences in the regulation and function of cellular immunity in Drosophila" has been editorially accepted for publication in PLOS Genetics. Congratulations!

Before your submission can be formally accepted and sent to production you will need to complete our formatting changes, which you will receive in a follow up email. Please be aware that it may take several days for you to receive this email; during this time no action is required by you. Please note: the accept date on your published article will reflect the date of this provisional acceptance, but your manuscript will not be scheduled for publication until the required changes have been made.

Once your paper is formally accepted, an uncorrected proof of your manuscript will be published online ahead of the final version, unless you’ve already opted out via the online submission form. If, for any reason, you do not want an earlier version of your manuscript published online or are unsure if you have already indicated as such, please let the journal staff know immediately at plosgenetics@plos.org.

In the meantime, please log into Editorial Manager at https://www.editorialmanager.com/pgenetics/, click the "Update My Information" link at the top of the page, and update your user information to ensure an efficient production and billing process. Note that PLOS requires an ORCID iD for all corresponding authors. Therefore, please ensure that you have an ORCID iD and that it is validated in Editorial Manager. To do this, go to ‘Update my Information’ (in the upper left-hand corner of the main menu), and click on the Fetch/Validate link next to the ORCID field.  This will take you to the ORCID site and allow you to create a new iD or authenticate a pre-existing iD in Editorial Manager.

If you have a press-related query, or would like to know about making your underlying data available (as you will be aware, this is required for publication), please see the end of this email. If your institution or institutions have a press office, please notify them about your upcoming article at this point, to enable them to help maximise its impact. Inform journal staff as soon as possible if you are preparing a press release for your article and need a publication date.

Thank you again for supporting open-access publishing; we are looking forward to publishing your work in PLOS Genetics!

Yours sincerely,

Pablo Wappner

Section Editor

PLOS Genetics

Aimée Dudley

Editor-in-Chief

PLOS Genetics

Anne Goriely

Editor-in-Chief

PLOS Genetics

www.plosgenetics.org

BlueSky: @plos.bsky.social

----------------------------------------------------

Comments from the reviewers (if applicable):

----------------------------------------------------

**Data Deposition**

If you have submitted a Research Article or Front Matter that has associated data that are not suitable for deposition in a subject-specific public repository (such as GenBank or ArrayExpress), one way to make that data available is to deposit it in the Dryad Digital Repository. As you may recall, we ask all authors to agree to make data available; this is one way to achieve that. A full list of recommended repositories can be found on our website.

The following link will take you to the Dryad record for your article, so you won't have to re‐enter its bibliographic information, and can upload your files directly:

http://datadryad.org/submit?journalID=pgenetics&manu=PGENETICS-D-25-00772R2

More information about depositing data in Dryad is available at http://www.datadryad.org/depositing. If you experience any difficulties in submitting your data, please contact help@datadryad.org for support.

Additionally, please be aware that our data availability policy requires that all numerical data underlying display items are included with the submission, and you will need to provide this before we can formally accept your manuscript, if not already present.

----------------------------------------------------

**Press Queries**

If you or your institution will be preparing press materials for this manuscript, or if you need to know your paper's publication date for media purposes, please inform the journal staff as soon as possible so that your submission can be scheduled accordingly. Your manuscript will remain under a strict press embargo until the publication date and time. This means an early version of your manuscript will not be published ahead of your final version. PLOS Genetics may also choose to issue a press release for your article. If there's anything the journal should know or you'd like more information, please get in touch via plosgenetics@plos.org.

# Editor report: Acceptance letter

**DOI:** 10.1371/journal.pgen.1012151.r006

**Authors:** Pablo Wappner, Pablo Wappner, Pablo Wappner

PGENETICS-D-25-00772R2

Sex differences in the regulation and function of cellular immunity in *Drosophila*

Dear Dr Tanentzapf,

We are pleased to inform you that your manuscript entitled "Sex differences in the regulation and function of cellular immunity in *Drosophila* " has been formally accepted for publication in PLOS Genetics! Your manuscript is now with our production department and you will be notified of the publication date in due course.

The corresponding author will soon be receiving a typeset proof for review, to ensure errors have not been introduced during production. Please review the PDF proof of your manuscript carefully, as this is the last chance to correct any errors. Please note that major changes, or those which affect the scientific understanding of the work, will likely cause delays to the publication date of your manuscript.

Soon after your final files are uploaded, unless you have opted out or your manuscript is a front-matter piece, the early version of your manuscript will be published online. The date of the early version will be your article's publication date. The final article will be published to the same URL, and all versions of the paper will be accessible to readers.

For Research Articles, you will receive an invoice from PLOS for your publication fee after your manuscript has reached the completed accept phase. If you receive an email requesting payment before acceptance or for any other service, this may be a phishing scheme. Learn how to identify phishing emails and protect your accounts at https://explore.plos.org/phishing.

Thank you again for supporting PLOS Genetics and open-access publishing. We are looking forward to publishing your work!

With kind regards,

Sharmila Kamatchi

PLOS Genetics

On behalf of:

The PLOS Genetics Team

Carlyle House, Carlyle Road, Cambridge CB4 3DN | United Kingdom

plosgenetics@plos.org | +44 (0) 1223-442823

plosgenetics.org | Twitter: @PLOSGenetics
